# Ablative Preoperative Single-Fraction Radiation Dose Escalation Among Patients With Breast Cancer: A Phase 1 Nonrandomized Clinical Trial

**DOI:** 10.1001/jamanetworkopen.2025.43689

**Published:** 2025-11-14

**Authors:** Asal Rahimi, Marilyn Leitch, Basak Dogan, Yulun Liu, Prasanna Alluri, Mona Arbab, Xingzhe Li, David D. M. Parsons, Dong Wook Nathan Kim, Narine Wandrey, Deborah Farr, Stephen Seiler, Nisha Unni, Anvy Nguyen, Rachel Wooldridge, Tsuicheng D. Chiu, Weiguo Lu, Strahinja Stojadinovic, Justin Visak, Chika Nwachukwu, Ina Patel, Howard Morgan, Shohreh Bahrami, Maggie Stein, Heather L. McArthur, Sunati Sahoo, Robert Timmerman

**Affiliations:** 1 Department of Radiation Oncology, University Texas Southwestern Medical Center, Dallas; 2 Department of Surgical Oncology, University of Texas Southwestern Medical Center, Dallas; 3 Department of Radiology, University of Texas Southwestern Medical Center, Dallas; 4 Department of Population and Data Sciences, University of Texas Southwestern Medical Center, Dallas; 5 Department of Radiation Oncology, Vanderbilt University Medical Center, Nashville, Tennessee; 6 Department of Medical Oncology, University of Texas Southwestern Medical Center, Dallas; 7 Department of Radiation Oncology, University of California San Diego School of Medicine; 8 Department of Radiation Oncology, CARTI Cancer Center, Little Rock, Arkansas; 9 Department of Pathology, University Texas Southwestern Medical Center, Dallas

## Abstract

**Importance:**

Single-fraction preoperative ablative stereotactic partial breast irradiation (sPBI) can be safely delivered in early-stage hormone receptor–positive (HR^+^) breast cancer with delayed time to surgery and high pathologic complete response rates.

**Objectives:**

To examine the maximum tolerated dose (MTD) of sPBI and to evaluate clinical outcomes, including pathological complete response (pCR), time to surgery, and toxic effects, associated with dose escalation.

**Design, Setting, and Participants:**

This phase 1 nonrandomized clinical trial of dose escalation enrolled patients with HR^+^, *ERBB2*-negative, cN0 invasive breast cancer not requiring chemotherapy from a single academic center between December 2019 and April 2024.

**Interventions:**

Patients were treated with 30, 34, or 38 Gy using MR-guided linear accelerator (MR-LINAC), robotic radiosurgery, or cobalt stereotactic unit. Patients received endocrine therapy and delayed surgery (≤12 months).

**Main Outcomes and Measures:**

The MTD was evaluated using dose-limiting toxicity (DLT), defined as grade 3 or higher toxic effects within 90 days. Additionally, pCR, near pCR (npCR), the association of dose escalation with time to surgery, local control, surgical morbidity, and cosmesis were evaluated.

**Results:**

A total of 44 patients (median [range] age, 64.5 [44.0-77.0] years) were treated, with 14 (31.8%) receiving 30 Gy (median [IQR] follow-up, 52.0 [28.2-54.5] months), 15 (34.1%) receiving 34 Gy (median [IQR] follow-up, 40.0 [36.5-41.0] months), and 15 (31.4%) receiving 38 Gy (median [IQR] follow-up, 20.0 [17.0-22.0] months). MTD was not reached. pCR rates were 35.7% (5 patients), 46.7% (7 patients), and 66.7% (10 patients), while pCR with npCR rates were 64.3% (9 patients), 93.3% (14 patients), and 93.3% (14 patients), respectively. Local control was 100%, and surgical morbidity was 2.2% (1 of 44 patients). The mean (SD) Ki-67 was 11.3% (6.4%) at diagnosis, and on evaluable residual disease, it was 1.9% (2.0%) (*P* < .001). A time-to-surgery threshold of 277 days was the optimal cutoff for pCR (area under the receiver operating characteristic curve, 0.77; 95% CI, 0.62-0.91). In patients with surgery more than 9 months after sPBI, pCR rates were 100% (5 of 5 patients), 66.7% (4 of 6 patients), and 64.3% (9 of 14 patients) (*P* = .40), and combined pCR and npCR rates were 100%, 83.3% (5 patients), and 92.9% (13 patients) (*P* = .69) for the 30, 34, and 38 Gy groups, respectively. For all patients who received surgery more than 9 months after sPBI, the pCR rate was 72.0% (18 of 25 patients). Longer time to surgery was associated with pCR (odds ratio, 1.02; 95% CI, 1.01-1.03; *P* = .005), while higher radiation dose was not. Acute toxic effects included 32 grade 1, 3 grade 2 (breast pain and dermatitis), and 1 late grade 3 (wound dehiscence in patient with uncontrolled diabetes) events. Cosmesis remained stable at 36 months.

**Conclusions and Relevance:**

In this nonrandomized clinical trial, the rate of toxic effects was low, and treatments were tolerable up to 38 Gy. Delaying surgery more than 9 months after sPBI with endocrine therapy was associated with increased pCR and npCR (>90%), while higher doses were not. These findings support further investigation of sPBI as a potential nonsurgical approach for selected patients with early-stage HR^+^ breast cancers.

**Trial Registration:**

ClinicalTrials.gov Identifier: NCT04040569

## Key Points

**Question:**

Can single-fraction preoperative ablative stereotactic partial breast irradiation be safely delivered in doses of 30, 34, and 38 Gy in early-stage hormone receptor–positive breast cancer?

**Findings:**

In this phase 1 nonrandomized clinical trial with 44 patients, the maximum tolerated dose was not reached up to 38 Gy. High pathologic complete response rates combined with near complete response rates (up to 93.3%) were observed with prolonged time to surgery (>9 months).

**Meaning:**

These findings suggest that stereotactic partial breast irradiation should be further investigated as a potential nonsurgical approach for selected early-stage hormone receptor–positive breast cancers.

## Teaser

This nonrandomized clinical trial uses a dose escalation framework to assess the maximum tolerated dose of preoperative ablative stereotactic partial breast irradiation and its associations with oncologic outcomes among patients with breast cancer.

## Introduction

Given excellent outcomes for luminal A breast cancer, deescalation of therapy can include reduced fractions^1^ or eliminating a mainstay of treatment, either radiation,^2,3^ endocrine therapy,^4,5^ or surgery.^6^ The optimal dose fractionation, and its association with pathologic complete response (pCR) rates, need to be determined prior to further trials with omission of surgery.

Preoperative radiation can reduce treatment volumes, assess biological response to radiation, and facilitate partial breast irradiation with oncoplastic surgery. A drawback is lack of untreated pathologic specimens, similar to after neoadjuvant chemotherapy. Delivering treatments in a single fraction rather than multiple fractions is more convenient to patients.

Studies of neoadjuvant chemotherapy or endocrine therapy in hormone receptor–positive (HR^+^) breast cancers have found pCR rates in the 1% to 17% range.^7,8^ As technology improves, stereotactic partial breast irradiation (sPBI) techniques and adaptive radiation give practitioners tools to immobilize the breast with geometric precision and sharper dose fall off,^9,10^ while safely delivering higher doses.^11^ Our previous phase 1 study using adjuvant sPBI, showed safety of up to 30 Gy in a single fraction.^12^ This was the basis of conducting an ablative trial with starting doses at 30 Gy.

Finding the correct single-fraction ablative dose poses a challenge, as the linear quadratic equation may not accurately estimate single-fraction doses. We used the universal survival curve to estimate the single-fraction equivalent of 70 Gy in 35 fractions, the dose routinely used for gross disease.^13^ This was calculated to be 38 Gy in 1 fraction using MCF-7 cells. To our knowledge, we are the first group to study the safety of these doses, starting at the inception of our trial in 2019. This trial sets the foundation for definitive ablative single-fraction radiation in early stage HR^+^ breast cancer by exploring the radiation dose required for high pCR rates, which can lead to trials assessing omission of surgery in excellent responders.

## Methods

### Study Design and Treatments

This is a phase 1 nonrandomized dose escalation trial of preoperative ablative sPBI for patients with early-stage luminal A HR^+^ breast cancer with delayed time to surgery, as safety for doses beyond 30 Gy in a single fraction have not been studied, to our knowledge, to date in breast cancer. Patients were treated in 3 dose cohorts of single-fraction 30, 34, or 38 Gy in groups of 7 to 15 patients, then received endocrine therapy 2 weeks after sPBI. Investigators adhered to Good Clinical Practice and ethical principles from the Declaration of Helsinki. Protocol was approved by the University of Texas Southwestern Medical Center institutional review board, and written informed consent was obtained. The trial protocol appears in Supplement 1. The reporting of this study followed the Transparent Reporting of Evaluations With Nonrandomized Designs (TREND) reporting guideline.

The study was designed to end if the rate of dose-limiting toxicity (DLT) within 90 days from the start of treatment was 33% or higher. A DLT is a grade 3 toxic effect deemed definitely related to treatment in the following categories: skin, rib (fracture), pulmonary (radiation pneumonitis), neurological (injury to nerves), or any grade 4 or 5 toxic effect deemed definitely attributed to therapy. Starting dose was 30 Gy. If none of the first 7 patients, 2 or fewer of first 9 patients, 3 or fewer of the first 12 patients, or 4 or fewer of the first 15 patients with 90-day follow-up experienced a DLT, then the dose would escalate to the next level. If 3 or more of the first 9 patients, 4 or more of the first 12 patients, or 5 or more of the first 15 patients experienced a DLT, then the maximum tolerated dose (MTD) would be considered exceeded. The MTD was defined as the immediately previous lower dose tolerated. The phase 1 portion of this trial was considered complete when either of the following events occur: (1) MTD for a cohort reached or (2) dose of 38 Gy, which investigators determined to be likely efficacious in controlling gross disease, is achieved and tolerated. Safety and toxic effects were assessed according to National Cancer Institute Common Toxicity Criteria for Adverse Events (CTCAE) version 5 for acute (<90 days) and late toxic effects directly related to treatment at 3, 6, 12, 24, and 36 months after radiation. Annual interim reports of adverse events were evaluated by a data safety monitoring committee to assess safety per the statistical plan.

### End Points

The primary end point was MTD or dose of 38 Gy in 1 fraction, whichever comes first. Secondary end points include pCR rates, association of prolonging time from radiation to surgery with pCR rates, local control (36 months from date of radiation to diagnosis of recurrence), acute toxic effects (within 3 months), late toxic effects (within 36 months), rate of surgical morbidity (within 36 months), patient- and physician-assessed cosmesis (36 months), and distant disease–free survival (date of registration to first distant disease up to 36 months). Cosmesis was obtained at baseline and 36 months using Harvard cosmesis scale (eAppendices 7 and 8 in Supplement 2). Photographs were obtained.

pCR was defined as Miller-Payne score (MPS) of 5 or residual cancer burden (RCB) score of 0; both scales were calculated for each patient. Near pCR (npCR), was defined as MPS 4 (corresponds to more than 90% reduction in tumor cellularity) or RCB I (also takes lymph nodes into account).^14,15^ Tertiary end points included radiomics on magnetic resonance imaging (MRI) to project pCR rates and translational correlates to estimate response rates.

**Figure 1.**
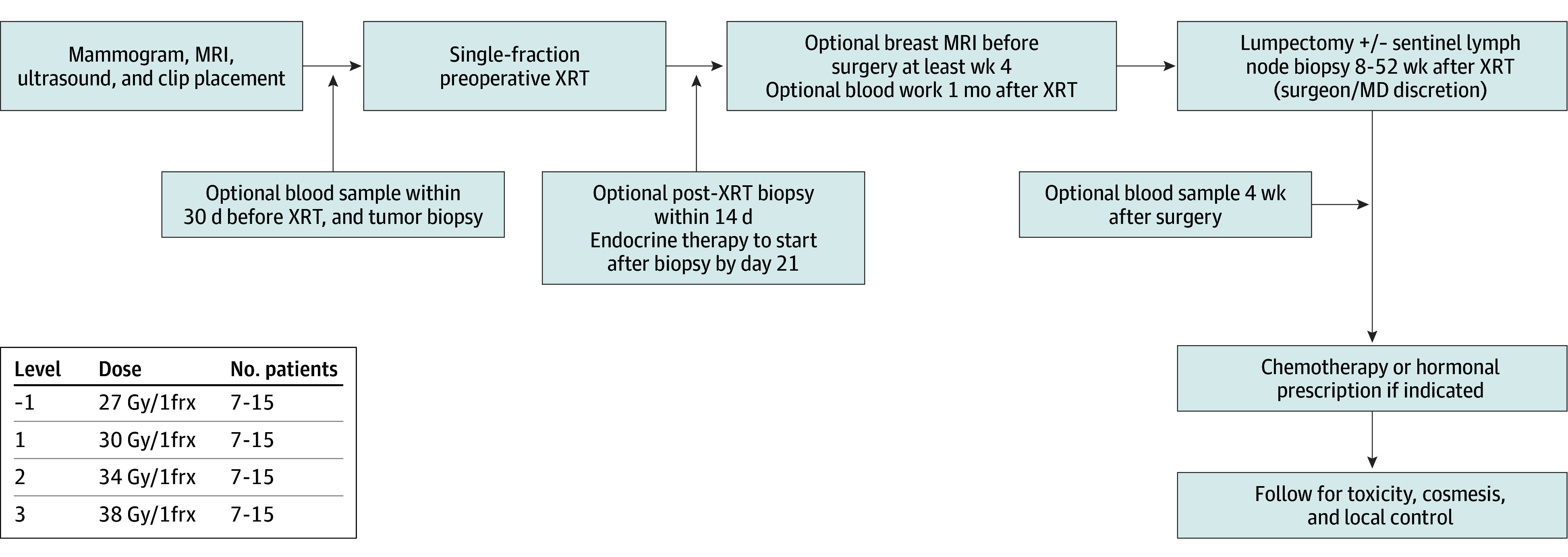
Study Schema MRI indicates magnetic resonance imaging; XRT, radiation therapy.

Eligible patients were aged 18 years or older with unifocal estrogen or progesterone receptor–positive and ERRB2-negative (formerly *HER2*/neu) invasive epithelial breast cancer (cT1 to cT2N0, according to the American Joint Committee on Cancer Staging Manual, 8th edition), greater than 5 mm away from the skin surface. Patients were excluded if they received neoadjuvant endocrine therapy, chemotherapy, or prior radiation in ipsilateral breast or had lupus or scleroderma. An oncotype was ordered at discretion of medical oncology (Figure 1).

### Radiation Treatment Dosage and Constraints

Patients were treated on Gammapod, Cyberknife, or MR-LINAC. Gross tumor volume (GTV) based on computed tomography simulation with intravenous contrast, biopsy clips, and diagnostic MRI volumes. Between 1 and 6 clips were placed to delineate the tumor. Breast radiologists helped with tumor delineation in many cases. Clinical target volume (CTV) was equal to GTV as stereotactic techniques used. The planning target volume was equal to CTV with a 5-mm expansion, cropped 5 mm away from skin and out of the chest wall. Doses were prescribed, so 95% of the PTV received a minimum of 27 Gy, and 99% of GTV received a minimum of 93% prescription. Skin constraints were prioritized (skin tolerance maximum dose, 27.5 Gy; <10 cc of skin receiving 25 Gy). Maximum allowable hot spot was less than 130% of the prescription dose (eAppendix 1 in Supplement 2).

### Assessments

#### Imaging Assessment of Response

All patients underwent mammography and breast ultrasound (US) at baseline and, if warranted, contrast enhanced dynamic breast MRI. Post-sPBI imaging follow-up with mammography and US were performed at 4 to 6 months and a preoperative MRI at 6 to 9 months as well as annually. The MRI protocol appears in eAppendix 2 in Supplement 2. Complete imaging response rate was defined as absence of enhancement on post-sPBI MRI.

#### Translational Correlates

Optional blood samples were collected at baseline, 1 month after sPBI, and at surgery. Optional tumor biopsies were performed at baseline, 2 weeks after sPBI, and at time of surgery if the tumor was evident. These findings will be reported in a separate article.

### Statistical Analysis

All statistical analyses conducted using R version 4.4.2 (R Foundation for Statistical Computing), including the survival package for Kaplan-Meier analysis and the pROC package for receiver operating characteristic (ROC) curve analysis. Paired *t* tests or Wilcoxon signed-rank tests evaluated changes in Ki-67 levels before and after radiation. Logistic regression analyses assessed the association of time to surgery and tumor size with MPS and pCR. ROC analysis, including area under the ROC (AUC), determined optimal thresholds and diagnostic accuracy for projecting responses. The Kaplan-Meier curve with log-rank test was conducted to compare pCR probabilities over time among dose cohorts, and McNemar and exact binomial tests assessed patient- and physician-rated cosmetic outcomes. All statistical tests were 2-sided, and *P* < .05 was considered statistically significant.

## Results

From December 2019 to April 2024, 44 patients were treated. A total of 14 patients (31.8%) were enrolled into the 30 Gy cohort, while 15 patients each (35.1%) were enrolled into the 34 and 38 Gy cohorts. The median (IQR) follow-up time was 52.0 (28.2-54.5), 40.0 (36.5-41.0), and 20.0 (17.0-22.0) months for the 30, 34, and 38 Gy groups, respectively. Median (range) age at enrollment was 64.5 (44.0-77.0) years (Table 1). All patients started endocrine therapy after sPBI, and 31 (70.5%) had oncotype before surgery. Two of 44 patients (4.5%) had pCR to primary tumor but were node-positive at surgery and received whole breast and regional nodal irradiation to 45 Gy over 25 fractions as well as chemotherapy.

**Table 1.** Patient and Tumor Characteristics

Characteristic	Patients, No. (%)			
	30 Gy (n = 14)	34 Gy (n = 15)	38 Gy (n = 15)	Total (N = 44)
Age, median (range), y	62.5 (44-77)	67 (45-76)	63 (47-77)	64.5 (44-77)
Median follow-up time, mo	52.0 (28.2-54.5)	40.0 (36.5-41.0)	20.0 (17.0-22.0)	32.5 (20.8-42.2)
cT1N0	13 (92.9)	14 (93.3)	14 (93.3)	41 (93.2)
cT2N0	1 (7.1)	1 (6.7)	1 (6.7)	3 (6.8)
Grade at diagnosis				
1	5 (35.7)	9 (60.0)	9 (60.0)	23 (52.3)
2	8 (57.1)	6 (40.0)	5 (33.3)	19 (43.2)
3	1 (7.1)	0	1 (6.7)	2 (4.5)
Oncotype performed	8 (57.1)	10 (66.7)	13 (86.7)	31 (70.5)
Endocrine therapy	14 (100)	15 (100)	15 (100)	44 (100)
GTV/CTV volume, median (range), cc	2.40 (0.73-13.94)	3.07 (1.12-7.48)	3.26 (0.94-7.74)	3.04 (0.73-13.94)
PTV volume, median (range), cc	23.01 (4.90-51.70)	16.8 (6.22-43.02)	11.48 (0.68-25.71)	16.70 (0.68-51.70)
Treated on MR LINAC	2 (14.3)	4 (26.7)	2 (13.3)	8 (18.2)
Treated on breast cobalt stereotactic unit, No. (%)	9 (64.3)	10 (66.7)	11 (73.4)	30 (68.2)
Treated on robotic radiosurgery unit, No. (%)	3 (21.4)	1 (6.6)	2 (13.3)	6 (13.6)

Abbreviations: CTV, clinical target volume; GTV, gross tumor volume; PTV, planning target volume.

### DLT, MTD, and Toxic Effects

Acute toxic effects for the 30 Gy cohort were 12 grade 1 events and 3 grade 2 events; for the 34 Gy cohort, they were 7 grade 1 events; while for the 38 Gy cohort, there were 13 grade 1 events. Late toxic effects were 1 grade 2 (breast pain) and 1 grade 3 dehiscence in a patient with uncontrolled diabetes, both in the 30 Gy cohort (Table 2). Highest grade toxic effects were queried for each patient. The MTD was not reached up to 38 Gy. Only 1 of 44 patients (2.2%) had surgical morbidity. This patient had a wound dehiscence after surgery and slow wound healing that persisted up to 6 months in the setting of uncontrolled diabetes.

**Table 2.** Acute and Late Toxic Effects for Dose Cohorts 30, 34, and 38 Gy

Outcomes	Toxic effects, No. (description)		
	Grade 1	Grade 2	Grade 3
**30 Gy cohort**			
Acute toxic effect	12 (dermatitis, pain, fatigue)	3 (dermatitis)	0
Late toxic effect	0	1 (breast pain)	1 (wound dehiscence)
**34 Gy cohort **			
Acute toxic effects	7 (dermatitis, pain, skin changes)	0	0
Late toxic effects	0	0	0
**38 Gy cohort **			
Acute toxic effects	13 (dermatitis, pain, hot flashes, hyperpigmentation)	0	0
Late toxic effects	0	0	0

### Secondary Analysis

#### pCR Rate, Distant Disease–Free Survival, Local Recurrence, and Ki-67 Levels

The overall median (range) time to surgery was 9.8 (2.9-12.0) months; for the 30, 34, and 38 Gy groups, it was 5.9 (2.8-11.7), 7.3 (5.3-12.0), and 11.4 (8.4-12.0) months, respectively. In the 30 Gy cohort, the pCR rate was 35.7% (5 patients), and npCR with pCR was 64.3% (9 patients). The 34 Gy cohort had a 46.7% pCR (7 patients) and 93.3% pCR with npCR (14 patients). The 38 Gy cohort had a 66.7% pCR (10 patients) and 93.3% npCR with pCR (14 patients) (Table 3). Patients enrolled later in the study had longer time to surgery based on evident patterns by the investigators of higher pCR rates.

**Table 3.** pCR and Time to Surgery for Each Dose Cohort

Outcome	Patients, No./total No. (%)			
	30 Gy (n = 14)	34 Gy (n = 15)	38 Gy (n = 15)	All cohorts (n = 44)
Time to surgery, median (range), mo	5.9 (2.8-11.7)	7.3 (5.3-12.0)	11.4 (8.4-12.0)	9.8 (2.8-12)
pCR	5/14 (35.7)	7/15 (46.7)	10/15 (66.7)	22/44 (50.0)
npCR and pCR	9/14 (64.3)	14/15 (93.3)	14/15 (93.3)	37/44 (84.1)
Patients with >9 mo to surgery after XRT				
pCR	5/5 (100.0)	4/6 (66.7)	9/14 (64.3)	18/25 (72.0)
npCR and pCR	5/5 (100.0)	5/6 (83.3)	13/14 (92.9)	23/25 (92.0)
Ki-67 <3% after surgery	9/9 (100.0)	7/8 (87.5)	4/4 (100.0)	20/21 (95.2)

Abbreviations: npCR, near pathological complete response; pCR, pathological complete response; XRT, radiation therapy.

When evaluating patients who had surgery more than 9 months after radiation, 100% (5 of 5 patients), 66.7% (4 of 6 patients) and 64.3% (9 of 14 patients) achieved pCR (*P* = .40) when treated with 30 Gy, 34 Gy, and 38 Gy, respectively. Likewise, 100%, 83.3% (5 patients), and 92.9% (13 patients) achieved pCR with npCR (*P* = .69) when treated with 30 Gy, 34 Gy, and 38 Gy, respectively.

The mean (SD) Ki-67 at diagnosis for all patients was 11.3% (6.4%), and on evaluable residual disease, it was 1.9% (2%) (*P* < .001). Of the 15 patients with an npCR, 53.3% had 3 mm or less of residual disease; 20 of 21 patients with residual disease (95.2%) had a Ki-67 level of less than 3% after surgery and sPBI (eAppendix 3 in Supplement 2). Local control and distant disease–free survival were 100% for all groups.

#### Association of Time to Surgery and Size of Tumor With pCR

Logistic regression analysis showed longer intervals before surgery were significantly associated with better responses (npCR: odds ratio [OR], 1.02; 95% CI, 1.00-1.03; *P* = .01; pCR: OR, 1.02; 95% CI, 1.01-1.03; *P* = .002). Tumor size was not associated with response (npCR: OR, 1.01; 95% CI, 0.88-1.16; *P* = .88; pCR: OR, 1.02; 95% CI, 0.93-1.12; *P* = .69) (eAppendix 4 in Supplement 2).

ROC curve analysis identified an optimal cutoff of day 155 (ie, 5.2 months) for achieving npCR with an AUC of 0.81 (95% CI, 0.56-1.00), a sensitivity of 0.97, a specificity of 0.71, a positive predictive value (PPV) of 0.95, and a negative predictive value (NPV) of 0.83. Day 277 (9.23 months) was identified as the optimal cutoff for achieving pCR (AUC, 0.77; 95% CI, 0.62-0.91; sensitivity, 0.82; specificity, 0.73; PPV, 0.75; and NPV, 0.80) (eAppendix 5 in Supplement 2).

Tumor size alone showed a limited ability to identify patients who would achieve pCR (AUC, 0.54; 95% CI, 0.37-0.72; sensitivity, 0.59; PPV, 0.59). However, combining tumor size with time to surgery improved the ability to project pCR, with an AUC of 0.77 (95% CI, 0.63-0.93), a sensitivity of 0.91, and a specificity of 0.64. These findings suggest that time to surgery was more strongly associated with treatment response than tumor size and integrating both variables would further enhance modeling of pCR (eAppendix 6 in Supplement 2).

#### Radiation Dose vs Time to Surgery

**Figure 2.**
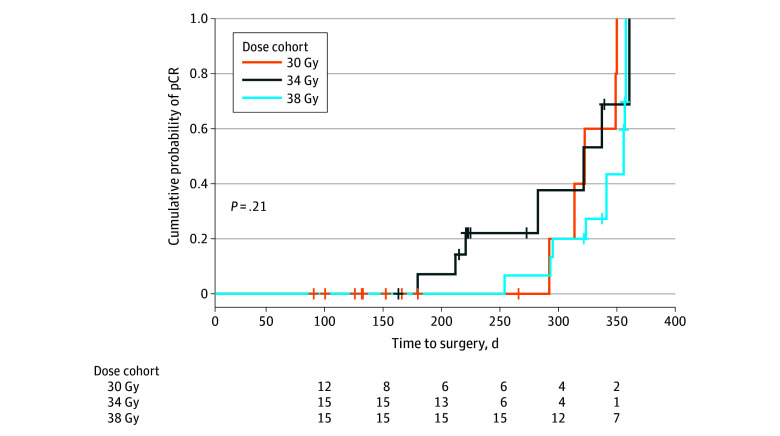
Cumulative Probability of Pathological Complete Response (pCR) With Extended Time to Surgery, Stratified by Dose Cohort

A Kaplan-Meier curve comparing time to surgery and probability of achieving pCR across radiation doses (30, 34, or 38 Gy) indicated no significant differences among dose groups (log rank *P* = .21) (Figure 2). A multiple logistic regression analysis found that time to surgery was significantly associated with higher likelihood of pCR (OR = 1.02; 95% CI, 1.01-1.03; *P* = .005), indicating each additional day before surgery increased the odds of pCR by approximately 1.7%. However, neither the 34 Gy cohort (OR, 1.09; 95% CI, 0.18-6.63; *P* = .93) nor the 38 Gy cohort (OR, 0.64; 95% CI, 0.08-4.99; *P* = .67) differed significantly from the 30 Gy cohort reference group regarding pCR.

#### Cosmesis

Patient and physician cosmesis was obtained at baseline and 36 months using the 4-point Harvard cosmesis scale (eAppendices 7 and 8 in Supplement 2). For the 30 Gy cohort, patient-rated cosmesis was excellent or good in 6 of 7 patients (85.7%) at baseline and 5 patients (71.4%) at 36 months, while physician-rated excellent or good cosmesis decreased from 7 patients (100%) to 6 patients (85.7%). In the 34 Gy cohort, patient-rated excellent or good cosmesis decreased from 8 of 8 patients (100%) to 5 patients (62.5%), whereas physician-rated excellent or good cosmesis remained stable at 8 patients (100%). Changes in patient-rated cosmesis were not statistically significant (30 Gy: *P* > .99; 34 Gy: *P* = .25). Physician-rated cosmesis showed no significant change in either cohort (both *P* > .99) (eAppendix 9 in Supplement 2). Findings indicate a nonsignificant decline in patient-rated cosmesis for the 34 Gy cohort, with stable physician ratings across both cohorts.

### Tertiary End Point: MRI and Response Assessment

Mean longest enhancement on pretreatment MRI was 1.3 cm (SD, 0.64 cm; 95% CI, 1.16-1.57 cm); at posttreatment MRI, it was 0.7 cm (SD, 0.9 cm; 95% CI, 0.3-1.0 cm) (*P* < .001). Of 22 patients who achieved pCR, 3 had no posttherapy MRI, 8 of the remaining 19 (42.1%) showed evidence of residual enhancement on the tumor bed. MRI was available in 34 of 37 patients with combined pCR and npCR; 18 patients (53.3%) had residual enhancement, while 16 (46.7%) had no enhancement in the tumor bed (OR, 0.93; 95% CI, 0.45-1.92). Conversely, only 4 of 7 patients (57.1%) with significant residual tumor (MPS of 3) showed residual enhancement at tumor bed.

## Discussion

Our primary end point of MTD in a single fraction was not met. Doses up to 38 Gy were deemed safe, with low rates of toxic effects. The most severe acute toxic effect was grade 2 breast pain or dermatitis, while the most severe late toxic effect was grade 3 dehiscence/slow-healing wound after surgery in a patient with uncontrolled diabetes. As the volumes of preoperative breast sPBI are at least 10 to 20 times smaller than a typical adjuvant partial breast plan^9^ and 100 times smaller than a whole breast plan, breast tissue can tolerate these high doses to small volumes, which is likely why higher dose cohorts did not have higher rates of toxic effects, particularly with a single fraction. Irradiated tissue is removed surgically months after the acute inflammation phase, which may have also helped with low late toxic effect rates and low surgical morbidity (2.2%). These high single-fraction doses will be useful for studies in which definitive radiation can be tested and other studies will need to test single- vs multifraction regimens on pCR rates.

Historical data for pCR after preoperative partial breast irradiation for HR^+^ breast cancer ranges from 0% to 42%.^16,17,18,19,20,21,22,23,24^ Our data differs from other studies in that our time to surgery was much longer, with a maximum of 12 months and median time to surgery 9.8 months in the overall cohort (11.4 months for 38 Gy cohort). Additionally, patients in this study received doses up to 38 Gy in a single fraction, which to our knowledge, is the highest dose reported to date. Our study is similar to the ABLATIVE trial,^25^ in that increasing time to surgery was associated with increased pCR rates. Increasing time to surgery from 6 to 8 months increased pCR rates from 33% to 48%,^25^ whereas in our study, we had pCR rates of 72% and 92% when combining npCR with pCR, among patients who underwent surgery 9 to 12 months after sPBI. The same group is now conducting the ABLATIVE-2 trial (NCT05350722), postponing surgery up to 12 months.

Our pCR rates for patients that had surgery more than 9 months after radiation were 100% in the 30 Gy arm vs no pCRs with less than 9 months to surgery. Our study has shown that surgery after more than 9 months was associated with pCR, and the association was stronger than tumor size or dose of greater 30 Gy. Our 34 Gy cohort also had increasing pCR rates with increasing time to surgery. In our 38 Gy cohort, most patients (14 of 15) had surgery between 9 and 12 months after sPBI, and this cohort had the highest overall pCR rate (66.7%) and npCR with pCR rate (93.3%). As we were able to elicit pCR rates in the 30 Gy cohort with time to surgery of more than 9 months, this suggests that time to surgery is likely more important than dose delivered and could lead to incomplete biological response at earlier time points. It is also important to note that for 95.3% of patients with npCR, Ki-67 was less than 3%, substantially lower than at diagnosis, showing that tumor cells are likely in the senescence phase and needed more time to complete their biological apoptosis. Our study also suggests that radiation-induced cancer death can take at least 1 year in the setting of endocrine therapy. Since most endocrine therapy is cytostatic, it is possible that these cells were further delayed to cycle into cell death by the endocrine therapy. To our knowledge, this is one of the first reports showing the importance of delayed time to surgery after breast sPBI to elicit a high level of pCR combined with npCR.^26,27^

We identified a significant reduction in the tumor size on post-treatment MRI (1.3 cm vs 0.67 cm; *P* < .001); however, we did not detect an association between residual enhancement and pCR status. Among patients achieving pCR, residual enhancement was observed in 42.1%, suggesting challenges in predicting pCR on the basis of imaging. Additionally, in patients with pCR and npCR, residual enhancement was present in 59.3%, highlighting the need to consider enhancement variability in evaluating posttherapy outcomes. Others have shown breast MRI to have a PPV of 67% to 88% (NPV, 76% to 85%).^14,18^ Only 57% of patients with MPS of 3 exhibited residual enhancement, underscoring MRI’s sensitivity limitation for detecting more extensive residual disease. This finding raises the need to investigate additional imaging modalities to characterize residual tumors. The NRG-BR005 trial also evaluated breast imaging and biopsy to predict pCR,^28^ which will be important as we move into more studies evaluating the role for radiation and omission of surgery.^6^

Finally, 2 patients (4.5%) were found to have axillary disease at surgery. Optimally, knowing the pathologic nodal status prior to preoperative radiation would strengthen this treatment paradigm, especially if we omit surgery for selected patients after ablative radiation. Better imaging modalities and nonoperative sentinel lymph node biopsy may be the answer to this. We are currently investigating this in our phase 2 ablative single-fraction preoperative radiation trial (NCT06444269).

### Limitations

This study has limitations. As a phase 1 trial assessing safety of dose escalation, it was not powered to differentiate all the secondary end points, and further studies will need to verify these results. Not all patients in the 38 Gy cohort have met the 36-month follow-up, so late toxic effects, cosmesis, and local control may be underascertained.

## Conclusions

This phase 1 study reaffirms preoperative ablative sPBI in a single fraction of 30- to 38 Gy as a safe regimen for early-stage HR^+^ breast cancer with low toxic effects and good cosmesis. Longer elapsed time from preoperative ablative sPBI with hormonal therapy to surgery (>9 months) was associated with higher pCR and npCR rates, suggesting potential tumor eradication with radiation and endocrine therapy alone. Incomplete biological response was seen at earlier time points. Longer than 9 months to surgery was more associated with pCR than doses greater than 30 Gy. These findings pave the way for possible nonsurgical treatment of selected patients with early stage HR^+^ breast cancer in the future.

## Notes

Supplement 1.
Trial Protocol

Supplement 2. **eAppendix 1. **Dosimetric Constraints for Single-Fraction SABR **eAppendix 2.** Imaging Assessment of Response **eAppendix 3. **Ki-67 of Biopsy Specimens at Diagnosis and After Preoperative Radiation and Delayed Surgery **eAppendix 4.** Univariate and Multiple Logistic Regression Results Evaluating the Association of Time to Surgery and Tumor Size With Dichotomized Miller Payne Scores **eAppendix 5.** Receiver Operating Characteristic (ROC) Curves for Miller Payne Scores **eAppendix 6.** ROC Analysis and Accuracy for pCR **eAppendix 7.** Patient Cosmesis **eAppendix 8.** Physician Cosmesis **eAppendix 9.** Patient and Physician Cosmesis Results

Supplement 3.
Data Sharing Statement
