# Supplementary material for: Ablative Preoperative Single-Fraction Radiation Dose Escalation Among Patients With Breast Cancer: A Phase 1 Nonrandomized Clinical Trial
Source: JAMA Netw Open. 2025 Nov 14;8(11):e2543689. doi: 10.1001/jamanetworkopen.2025.43689 (PMC12619099; doi:10.1001/jamanetworkopen.2025.43689)
Supplement: Supplement 1. — Trial Protocol [file jamanetwopen-e2543689-s001.pdf]

STU 2019-1183

A Phase I Dose Escalation Study of Single Fraction Pre-operative Partial Breast (S-PBI) for Early Stage Breast Cancer

Principal Investigator: Asal Rahimi, MD
Department of Radiation Oncology
University of Texas Southwestern Medical Center
2201 Inwood Road
Dallas, TX 75390
214-645-8556
Asal.rahimi@utsouthwestern.edu

Co-Principal Investigators or Lead Sub-Investigator(s):

Radiation Oncology
Prasanna G. Alluri, MD, PhD (Clinical sample procurement and translational studies)

Surgical Oncology
Ann Marilyn Leitch MD (Lead)

Medical Oncology
Heather McArthur, MD

Radiology
Basak Dogan MD (lead PHHS)

Pathology
Sunati Sahoo MD (Lead)

Biostatistician: Statistics
Yulun Liu PhD
UTSW
214-648-7438
Yulun.Liu@utsouthwestern.edu

Study Drug/Treatment: Preoperative radiation for breast cancer

Funding Source: Departmental Support

NCT Number: NCT04040569

Initial version: **[V1.0 7/24/2019]**
Amended: **[V2.0 03/16/2020]**
[V3.0 10/26/2020]
[V4.0 11/01/2021]
[V5.0 8/03/2022]
[V5.1 01/10/2023]
[V6.0 08/30/2023]

UT Southwestern Medical Center (UTSW)
Department of Radiation Oncology
Attn: Clinical Research
2280 Inwood Rd.
Dallas, Texas 75390-9179

CONFIDENTIAL
This material is the property of the UTSW Simmons Comprehensive Cancer Center.
Do not disclose or use except as authorized in writing by the study sponsor.
Template Updated: 12/2012; 9/2013; 8/2014; 4/2015; 10/2015; 11/2016; 12/2018; 07/2020; 07/2022

Signature Page

The signature below constitutes the approval of this protocol and the attachments, and provides the necessary assurances that this trial will be conducted according to all stipulations of the protocol, including all statements regarding confidentiality, and according to local legal and regulatory requirements and applicable U.S. federal regulations and ICH guidelines.

Amendment/Version #______6.0______________________

PROTOCOL NUMBER: STU 2019-1183

A Phase I Dose Escalation Study of Single Fraction Pre-operative Partial Breast (S-PBI) for Early Stage Breast Cancer

Principal Investigator (PI) Name: _____________________________

PI Signature: _____________________________

Date: ____________________

CONFIDENTIAL
This material is the property of the UTSW Simmons Comprehensive Cancer Center.
Do not disclose or use except as authorized in writing by the study sponsor.
Template Updated: 12/2012; 9/2013; 8/2014; 4/2015; 10/2015; 11/2016; 12/2018; 07/2020; 07/2022

TABLE OF CONTENTS

LIST OF ABBREVIATIONS **1**

STUDY SCHEMA **2**

STUDY SUMMARY **3**

1.0 BACKGROUND AND RATIONALE **4**

1.1 Disease Background and Rationale 4

1.2 Study Therapy Background and Associated Known Toxicities 4

1.3 Study Rationale 7

2.0 STUDY OBJECTIVES **10**

2.1 Primary Objectives 10

2.2 Secondary Objectives 10

2.3 Exploratory Objectives 10

2.4 Endpoints 11

3.0 SUBJECT ELIGIBILITY **11**

3.1 Inclusion Criteria 11

3.2 Exclusion Criteria 12

4.0 TREATMENT PLAN **12**

4.1 Treatment Dosage and Administration 12

4.2 Toxicities and Dosing Delays/Dose Modifications 21

4.3 Concomitant Medications/Treatments 21

4.4 Duration of Therapy 22

4.5 Duration of Follow Up 22

4.6 Removal of Patients from Protocol Therapy 22

4.7 Subject Replacement 22

5.0 STUDY PROCEDURES **23**

5.1 Screening/Baseline Procedures 23
5.2 Procedures During Treatment 25
5.3 Follow-up Procedures 26
5.4 Time and Events Table 27
5.5 Removal of Subjects from Study 28

6.0 MEASUREMENT OF EFFECT 28
6.1 Antitumor Effect-Solid Tumors 28
6.2 Criteria for Cosmetic Outcome Photography 30
6.3 Safety/Tolerability 31

7.0 ADVERSE EVENTS 31
7.1 Experimental Therapy 31
7.2 Adverse Event Monitoring 32
7.3 Steps to Characterize a Serious Adverse Event for Reporting to the SCCC DSMC 34
7.4 Stopping Rules 37

8.0 MISCELLANEOUS INFORMATION 37
8.1 Lumpectomy Guidelines 37

9.0 CORRELATIVES/SPECIAL STUDIES 37
9.1 Optional Sample Collection Guidelines 38
9.2 Specimen Banking 38

10.0 STATISTICAL CONSIDERATIONS 39
10.1 Study Design/Study Endpoints 39
10.2 Sample Size and Accrual 40
10.3 Data Analyses 40

11.0 STUDY MANAGEMENT 41
11.1 Conflict of Interest 41
11.2 Institutional Review Board (IRB) Approval and Consent 41

11.3 Registration/Randomization Procedures 41

11.4 Data Management and Monitoring/Auditing 42

11.5 Adherence to the Protocol 42

11.6 Amendments to the Protocol 44

11.7 Record Retention 44

11.8 Obligations of Investigators 44

12.0 REFERENCES 45

13.0 APPENDICES 50

LIST OF ABBREVIATIONS

AE	Adverse Event
BCS	Breast Conservation Surgery
CR	Complete Response
CT	Computed Tomography
CTCAE	Common Terminology Criteria for Adverse Events
CTV	Clinical Target Volume
DLT	Dose Limiting Toxicity
DOT	Disease Oriented Team
DSMC	Data and Safety Monitoring Committee
FDA	Food and Drug Administration
GCP	Good Clinical Practice
GTV	Gross Target or Tumor Volume
HRPP	Human Research Protections Program
IIT	Investigator-Initiated Trial
IHC	Immunohistochemistry
IV (or iv)	Intravenously
MRI	Magnetic Resonance Imaging
MTD	Maximum Tolerated Dose
OS	Overall Survival
pCR	Pathologic Complete Response
PD	Progressive Disease
PFS	Progression Free Survival
p.o.	peros/by mouth/orally
PR	Partial Response
PTV	Planning Target Volume
SAE	Serious Adverse Event
SCCC	Simmons Comprehensive Cancer Center
WBI	Whole Breast Irradiation

STUDY SCHEMA

Phase I Study of Pre-Operative Stereotactic Partial Breast Irradiation Therapy for Early Stage Breast Cancer

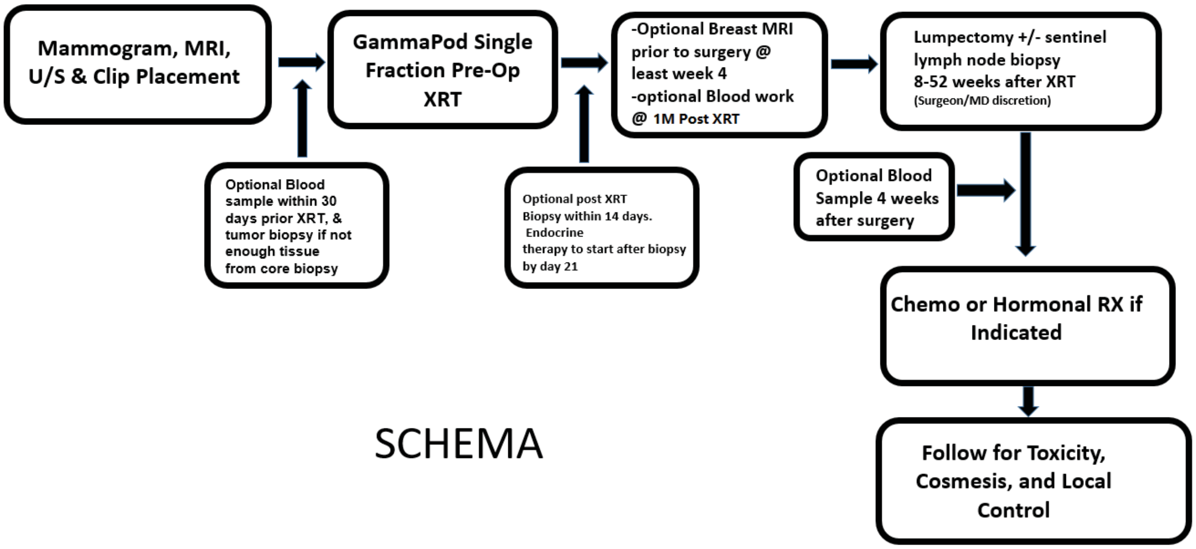

**Of note, after pre-operative radiation and prior to surgery, patients should start endocrine therapy within 21 days after pre-operative radiation but it is encouraged to start the day after post-radiation biopsy.

Prescription will be 30 Gy to the Planning Target Volume (PTV) for all dose cohorts, and either 30 Gy, 34 Gy, or 38 Gy to the GTV/CTV per appropriate dose cohort.

Number of patients= between 7-60 for phase I (depending on tolerance)
All patients in each dose cohort will be treated as a single group for dose escalation. The starting dose for the dose escalation portion will be 30 Gy. Subsequent cohorts of patients will receive an additional 4 Gy per treatment. If significant toxicity is encountered at the starting dose, a de-escalation will occur (step -1) to 26.5 Gy. As we are currently concluding an adjuvant single fraction phase I protocol (ClinicalTrials.gov Identifier: NCT02685332) to 30 Gy in a single fraction for early stage breast cancer, if we meet our endpoints of this study, we will start our dose escalation at 34 Gy instead of the 30 Gy, as safety of the 30 Gy arm will have already been established.

Level	Dose	No. of Patients
-1	26.5 Gy	7-15
1	30 Gy	7-15
2	34 Gy	7-15
3	38 Gy	7-15

- Prescription will be a minimum of 27 Gy to the PTV for all dose cohorts, and either 30 Gy, 34 Gy, or 38 Gy to the GTV/CTV per appropriate dose cohort. See section 4.1.5

Minimum waiting periods will be assigned between each dose cohort to evaluate toxicity. The phase I study will be completed when dose limiting toxicity is reached or when delivery of a pre-determined highest dose of radiation (38 Gy) that has been deemed likely to be efficacious for treatment of early stage breast cancer is attained.

At the time of writing this protocol, the current single fraction trial being tested in the adjuvant setting for early stage breast cancer is 30 Gy in 1 fraction at UTSW (NCT02685332). If we meet our accrual goal and waiting period without a dose limiting toxicity by the time this protocol is ready to go live, we will start our dose for this trial at 34 Gy.

STUDY SUMMARY

Title	A Phase I Dose Escalation Study of Single Fraction Pre-operative Partial Breast (S-PBI) for Early Stage Breast Cancer
Short Title	Single Fraction Pre-op SBRT Dose Escalation Study for Breast Cancer
Protocol Number	STU-2019-1183
Phase	1
Methodology	Prospective Dose Escalation
Study Duration	5 years
Study Center(s)	University of Texas Southwestern Medical Center
Objectives	Determine the Maximum tolerated dose of single fraction pre-op breast SBRT
Number of Subjects	Between 7-60 patients (depending on tolerance)
Diagnosis and Main Inclusion Criteria	Invasive epithelial (ductal, medullary, lobular, papillary, mucinous (colloid), or tubular histologies of the breast 3 cm or less T1-T2cN0 whom have not undergone surgery or neoadjuvant systemic therapy.
Study Product(s), Dose, Route, Regimen	Radiation, Stereotactic Body Radiation Therapy<br>1 fraction SPBI, dose escalation 3.5-4 Gy per cohort<br>30Gy-→38Gy (3 dose cohorts, potentially 2 if 30 Gy dose is proven safe on current ongoing trial before the start of this trial)
Duration of administration	One day
Reference therapy	Postoperative Single fraction Radiation Therapy
Statistical Methodology	For each dose level cohort, a total of 7-15 patients will be enrolled. The dose-limiting toxicity (DLT) and the maximum tolerable dosage (MTD) will be determined based on this Phase I design. Exact binomial method will be used to calculate the response rate, toxicity and the corresponding 95% confidence interval. Kaplan-Meier method will used to estimate the distant disease-free interval.

1.0 BACKGROUND AND RATIONALE

1.1 Disease Background and Rationale

Introduction

Breast conservation surgery (BCS) with whole breast irradiation (WBI) results in equivalent survival to mastectomy in early stage breast cancer patients (Fisher NEJM 2002; van Dongen JNCI 2000, Veronesi NEJM 2002). Although BCS with adjuvant WBI is more desirable for many women with breast cancer, the duration of treatment, which can range from 3-6.5 weeks, presents a hurdle for some patients. In particular, transportation difficulties, financially strains and advanced age may present barriers to conventional whole breast irradiation. In fact, a recent analysis of SEER data has shown that approximately 18% of patients who underwent lumpectomy between 2004 and 2009 did not receive adjuvant radiation (Yeboa Am J Clin Oncol 2016). Though there has been recent debate on the necessity of adjuvant radiation in the elderly (Hughes JCO 2013; Kunkler Lancet Oncol 2015), it is important to note that adjuvant WBI has been shown to both improve local control (Fisher NEJM 2002) and overall survival (EBCTCG Trialist's Group Lancet 2011), emphasizing its importance in appropriate cohorts electing to undergo BCS. To overcome the limitation of long duration of treatment of conventional whole breast irradiation, significant efforts have recently been focused on developing hypofractionated WBI (START Trialist's Group Lancet 2008a and 2008b; Whelan NEJM 2010). Such regimens (40Gy/15Fx and 42.5Gy/16Fx with or without a boost) have been accepted as equivalent to conventional WBI regimens for large groups of patients. Although these regimens have reduced the burden of treatment duration of 6-6.5 weeks, current hypofractionated whole breast irradiation regimens of 3-4 weeks (Smith Pract Radiat Oncol 2018) continue to present a barrier receipt of adjuvant radiation therapy following BCS to some patients.

1.2 Study Therapy Background and Associated Known Toxicities

Adjuvant Partial Breast Irradiation (PBI)

Partial breast irradiation (PBI) offers promising results with several regimens employed over the course of approximately 1 week reducing treatment time further; however, there is still debate in regards to the optimum regimen and modality. Interest in PBI arose after noting that most local recurrences tend to occur within the same quadrant in early trials (Fisher Semin Surg Oncol 1992) and that malignant cells tend to be within 1 cm of the resection margin in a pathologic analysis (Vicini IJROBP 2004). There was a question if the target volume could be reduced to this high-risk region around the lumpectomy cavity, which could allow further hypofractionation given reduced volume of normal tissue irradiated. Several PBI methods have thus been investigated, including interstitial, intracavitary, intraoperative (IORT), and 3-dimensional (3D) conformal radiotherapy (CRT).

Interstitial brachytherapy involves the insertion of several temporary catheters into the lumpectomy site with the objective of delivering radiation locally via HDR to achieve local control. This method was evaluated in GEC-ESTRO, a phase III non-inferiority trial, where interstitial brachytherapy was found to have a similarly low incidence of ipsilateral breast tumor recurrence (IBTR) when compared to conventionally fractionated WBI at 5 years (1.4% vs 0.92%), meeting non-inferiority criteria (Strnad Lancet 2016). A smaller study in Hungary corroborated these results (Polgar IJROBP 2007). Intracavitary brachytherapy has also been explored, given it is technically easier to perform. It involves the insertion of a balloon-based or strut-based catheter into the lumpectomy bed with the subsequent delivery of radiation via HDR. This intracavitary method has been shown in several non-randomized experiences to achieve adequate local control (Yashar Brachytherapy 2016; Vargo IJROBP 2014).

However, both interstitial and intracavitary brachytherapy are limited by operator experience, risk of infection and wound healing, and inconvenience of having a foreign object inserted for >1 week. Consequently, there has been interest in exploring APBI via external beam techniques, given these techniques would be more readily available and potentially avoid the need for an additional procedure and foreign body insertion. External beam techniques which have been evaluated include 3D-CRT (Olivotto JCO 2013; Whelan SABCS Symposium 2018), mini-tangents (Coles Lancet 2017), five-fraction IMRT (Livi Eur J Cancer 2015), and stereotactic PBI (S-PBI). 3D-CRT has been investigated in the RAPID Trial and NSABP 39/RTOG 0413. In the RAPID Trial, 2135 women with node-negative early stage breast cancer were randomized to APBI 38.5Gy/10Fx BID or WBI (predominantly 42.5Gy/16Fx). Cosmesis was noted to be worse at five years (nurse reported fair/poor ratings 32.8% vs 13.4%) in the APBI group (Olivotto JCO 2013); however, IBTR was similar and met the non-inferiority criteria at 8-year follow presented at SABCS 2018 (Whelan SABCS Symposium 2018). Most recently, the 10 year results of NSABP 39/RTOG 0413 was presented at SABCS 2018 showing that patients randomized to PBI (brachytherapy 34Gy/10Fx BID or 3D-CRT 38.5Gy/10Fx BID) did not meet equivalence criteria when compared to WBI. However, the difference was small with 10 year IBTR-free survival being 95.2% (vs 95.9%) and 10 year RFI was 91.9% (vs 93.4%), but PBI had higher grade 3 toxicity 9.6% (vs 7.1%) (Vicini SABCS Symposium 2018).

Notably, there has also been interest in IORT given the potential to reduce the burden of radiation to 1 day, which has been assessed in the ELIOT (Veronesi Lancet Oncol 2013) and TARGIT-A trials. However, this modality has not been widely adopted due to lack of accessibility and note of a small but significant IBTR detriment with IORT. These single-fraction experiences have provided a foundation for subsequent stereotactic partial breast trials (which will be discussed in subsequent sections).

Adjuvant Stereotactic Partial Breast Irradiation (S-PBI)
Although various PBI trials have demonstrated adequate local control, the largest trial with 10-year follow-up, NSABP 39/RTOG 0413 (Vicini SABCS Symposium 2018), has suggested a minor detriment in IBTR and RFI when comparing PBI and WBI, with higher rates of grade 3 toxicity. In congruence with these higher rates of toxicity, the RAPID trial also reported worse cosmesis with 3D-CRT PBI (Olivotto JCO 2013). These discrepancies in tumor control and toxicity raised the question if 34-38.5Gy/10Fx BID is the most adequate dose/regimen selected. Noting that stereotactic PBI (S-PBI) could offer the potential for smaller target volumes given its use of real-time imaging and respiratory tracking to reduce treatment setup uncertainties and the need for a PTV, S-PBI has been recently investigated as these smaller target volumes may allow for further dose escalation.

UTSW has been one of the pioneers in the S-PBI techinique. Some adjuvant S-PBI retrospective reports published 30Gy/5Fx as a feasible dose (Vermeulen Front Oncol 2011; Vermeulen Transl Cancer Res 2014; Obayomi-Davies Front Oncol 2016). Specifically, 21 patients were treated with 25-36Gy/5-10Fx at the Swedish Medical Center, 26 patients treated with 30Gy/5Fx at Winthrop University (Vermeulen Transl Cancer Res 2014), and an additional 10 patients were treated with 30Gy/5Fx at Georgetown (Obayomi-Davies Front Oncol 2016). All of the patients in these cohorts reported good or excellent cosmetic outcomes, and none reported grade 3 or higher toxicity. These experiences suggested that 30Gy/5Fx was a safe and feasible dose, which led to a subsequent dose escalation trial at the University of Texas Southwestern (UTSW) noting that 40Gy/5Fx was biologically equivalent to the conventional WBI dose of 50Gy/25Fx with a boost of 10Gy/5Fx on the universal survival curve. Seventy-seven patients with 75 analyzable over 5 dose cohorts; 30Gy, 32.5Gy, 35Gy, 37.5Gy, and 40Gy in 5 fractions, were included in this phase I dose escalation study that started in 2010. At a median follow-up of 26.1 months, ten patients had developed fat necrosis which was associated with larger PTV volumes, with a cutoff point of 124 cm^3 or higher noted to have a higher probability of being associated with fat necrosis across dose levels. Only 1 dose-limiting toxicity was encountered and MTD was not reached, with most patients reporting excellent or good cosmesis (Rahimi IJROBP 2017). Following the report of the

STU2019-1183, Rahimi, FormA-Protocol, Mod_15, 11-01-23 (7)

safety and feasibility of adjuvant S-PBI 40Gy/5Fx, UTSW has begun investigating single-fraction adjuvant S-PBI in a dose escalation study, with the results eagerly awaited.

Oncoplastic Reconstruction and Breast Reduction with BCS: An Obstacle to PBI
Although there has been significant advancement in PBI, APBI, and S-PBI, there has been a recent rise in the incidence of oncoplastic reconstructions which poses an obstacle to the delineation of the lumpectomy cavity (Pezner Am J Clin Oncol 2013), which results in larger target volumes than previously encountered, and surgical clips that are not in just one quadrant of the breast, making targeting the lumpectomy cavity more difficult. Given that larger PTV volumes have been associated with a higher incidence of fat necrosis (Rahimi IJROBP 2017), this reconstructive technique may pose an issue in relation to adjuvant S-PBI toxicity and delineation of the cavity for any partial breast technique or boost. This technique has been especially prevalent at our institution, but has also been reported to be increasing nationally in a recent ACS-NSQIP database analysis of surgical trends that demonstrated an increase from 4% to 9% of oncoplastic reconstruction in those receiving BCS between 2005 to 2016 (Jonczyk Breast Cancer Res Treat 2019). However, it should be noted that BCS + oncoplastic reconstruction has been associated with lower re-excision rates than BCS alone (RR=0.66) in a meta-analysis of 3789 patients (Chen J Breast Cancer 2018) and excellent/good cosmesis in approximately 86% of women in a systematic review of 1962 patients (De La Cruz Ann Surg Oncol 2016). Although data for oncoplastic reconstruction is non-randomized, results are promising, making it important to adapt to this surgical trend in breast oncologic care.

Preoperative Stereotactic Partial Breast Irradiation (preoperative S-PBI)
Preoperative stereotactic breast radiation may be beneficial as it offers the ability to target smaller treatment volumes than what has been achievable with adjuvant PBI (Nichols IJROBP 2010), track radiobiological response to radiation at time of surgical pathology, and allow the removal of all irradiated tissue to potentially minimize late effects. Note that non-stereotactic preoperative PBI is also being investigated (Nichols IJROBP 2017; van der Leij Radiother Oncol 2015), but the traget volume expansions tend to be larger compared to S-PBI given technical differences in treatment modalities, and the pCR rates tend to be poor, with 15% achieving pCR in 27 patients treated with preoperative 38.5Gy/10Fx BID at the University of Maryland (Nichols IJROBP 2017).

The first report of preoperative S-PBI was at the Centre Antoine Lacassagne in France, where Bondiau et al conducted a phase I dose-escalation study of 19.5Gy in 3 fractions and 22.5Gy in 3 fractions in 6 patients with initially inoperable breast tumors. All these patients received neoadjuvant chemotherapy concurrently with 3 cycles of docetaxel 100mg/m^2 and then 3 cycles of fluorouracil, epirubicin, and cyclophosphamide, with radiation given during the second cycle of docetaxel. All patients subsequently had BCS and adjuvant WBI of 50Gy in 25 fractions. Two patients had clinical complete responses (cCR) and four had clinical partial responses (PR). Mean tolerated dose (MTD) was not reached (Bondiau IJROBP 2009). This led to an expanded phase I dose escalation study by Bondiau et al including 19.5, 22.5, 25.5, 28.5, and 31.5Gy in 3 fraction cohorts with 25 patients enrolled. The same chemotherapy regimen was utilized with preoperative S-PBI again being delivered during the second cycle of docetaxel. At a median follow-up time of 30 months, there were 36% pathologic complete responses (pCR), noting 67% pCR in the 25.5Gy cohort. MTD was not reached, noting that one patient experienced grade 3 dermatologic dose-limiting toxicity (Bondiau IJROBP 2013). This study demonstrated safety up to 31.5 Gy in 3 fractions of preoperative S-PBI in the setting of concurrent neoadjuvant chemotherapy.

More recently, a group at Duke investigated single fraction preoperative S-PBI. They initially performed a dosimetric study of 17 patients demonstrating dosimetric feasibility of CyberKnife-based single-fraction preoperative S-PBI in patients with tumors <1.0 cm (Palta IJROBP 2012). This led to a phase I dose escalation trial, where thirty-two women with clinically-node negative, <2.0 cm in diameter, low to intermediate grade DCIS or invasive breast cancer were included. All patients were ER-positive or PR-positive and HER2-negative. These patients were divided into 3 dose cohorts, with 8 patients receiving 15 Gy, 8 receiving 18 Gy, and 16 receiving 21 Gy in 1

fraction. Contouring was done predominantly with prone CT and MRI simulation, with a CTV of 1.5 cm and a PTV of 0.3 cm minus 0.5 cm of subcutaneous tissue, achieving a median PTV volume of 63 mL (range: 31-97). During the analysis, they noted that not all lesions on CT were readily visible and that there were differences in size between CT and MRI scans. They concluded that MRI is the preferred evaluation method for preoperative breast radiation secondary to usefulness in target delineation and elimination of inappropriate patients (Blitzblau Pract Radiat Oncol 2015). At a median follow-up of 23 months, no dose-limiting toxicity was encountered. Cosmesis was excellent or good in all patients receiving preoperative radiation alone, while it was fair/poor in the 3 patients that received both preoperative and adjuvant radiation (Horton IJROBP 2015). This phase I dose escalation trial demonstrated the safety of 21 Gy in 1 fraction of preoperative S-PBI.

1.3 Study Rationale

Given interest in preoperative S-PBI, there have been several recently initiated preoperative S-PBI trials that have opened and are ongoing (see Table 1). However, these ongoing preoperative trials are predominantly using doses seen in the adjuvant setting as established with IORT, primarily 21Gy/1Fx, although a 3-fraction and 5-fraction regimen are also currently being investigated at Moffit (NCT03137693) and the Juravinski Cancer Center (NCT02065960), respectively. Pathologic complete response rates with three-fraction preoperative S-PBI have been reported to be 36%, with the highest pCR rate of 67% in the 25.5Gy/3Fx cohort, though this cohort was small with only 6 patients (Bondiau IJROBP 2013), suggesting room for improvement. At ESTRO in 4-2019, the results from the Netherlands (clinical trials.gov: NCT0231661) ABLATIVE trial treating patients with Estrogen positive her2- tumors and 20 Gy preoperative radiation yielded a path CR of 33% at 6 months and 41% at 8 months with mild treatment toxicity (ESTRO abstract OC-0591 Vasmel et al.)

As predicted by the universal survival curve, 38Gy/1Fx would be equivalent to 70Gy/35Fx, a dose that is typically used for gross disease (Park IJROBP 2008). Given that these early studies have reported minimal toxicity with the addition of preoperative S-PBI, this offers promise that further dose escalation to achieve a radioablative dose adequate for gross disease may be feasible.

Therefore, the purpose of this phase I trial is to evaluate dose-limiting toxicity while dose escalating single-fraction preoperative S-PBI to a presumed radioablative dose over 3 cohorts, starting with 30Gy in 1 fraction and advancing to 34Gy and 38Gy in 1 fraction. This would be accomplished on the MR Linac, CyberKnife, or GammaPod. The GammaPod is a novel device dedicated to S-PBI utilizing a Cobalt-60 source (Yu Med Phys 2013), which offers a highly reproducible prone setup with a mean of 1.8mm of mismatch reported in 15 patients at the University of Maryland on consecutive scans (Yu JCO 2011). Implications of this research are far reaching, including determination of the maximally tolerated dose for preoperative S-PBI, pathologic complete response rates of early stage breast cancer to a predicted radioablative dose, radiographic markers of treatment response (radiomics), and translational research assessing mechanisms of immune and radiation response.

Tables

Institution	Principal Investigator	Study Design	Study Start Date	Anticipated Accrual
**Juravinski Cancer Center**<br><br>**ARTEMIS Trial**<br><br>(NCT02065960)	Julie Arsenault, MD<br>Do-Hoon Kim, MD<br>Timothy Whelan, MSc	Phase I trial of preoperative 40Gy/5Fx S-PBI	February 2014	32
**Laurentian University, Jewish General Hospital**<br><br>**SIGNAL Trial**<br><br>(NCT02212860)	Muriel Brackstone, MD PhD	Phase II trial of preoperative 21Gy/1Fx S-PBI	March 2015	120
**Duke University**<br><br>(NCT02482376)	Rachel Blitzblau, MD PhD	Phase II trial of preoperative 21Gy/1Fx S-PBI	October 2015	100
**H Lee Moffit Cancer Center**<br><br>(NCT03137693)	Michael Montejo, MD	Phase II trial of preoperative 3-fraction S-PBI	April 2017	40
**Sidney Kimmel Comprehensive Cancer Center at Johns Hopkins**<br><br>(NCT03043794)	Jean Wright, MD	Phase II trial of preoperative S-PBI of 21Gy/1Fx	August 2017	40
**Azienda Opsedaliero-Universitaria Careggi**<br><br>(NCT03520894)	Lorenzo Livi	Single-arm phase II trial of 21Gy/1Fx preoperative S-PBI	Not yet recruiting	25

Table 1. Ongoing preoperative stereotactic partial breast irradiation (preoperative S-PBI) trials.

STU2019-1183, Rahimi, FormA-Protocol, Mod_15, 11-01-23 (7)

Institution	Principal Investigator	Study Design	Study Start Date	Anticipated Accrual
**Georgetown University**<br><br>(NCT02365714)	Sonali Rudra, MD	Phase I-II trial of adjuvant 30Gy/5Fx S-PBI	February 2015	Enrolled 2 (Status: Terminated due to slow accrual)
**Georgetown University**<br><br>(NCT02457117)	Olusola Obayomi-Davies, MD	Multi-institutional registry of adjuvant 30Gy/5Fx S-PBI	May 2015	200
**University of Texas Southwestern**<br><br>(NCT02685332)	Asal Rahimi, MD	Phase I dose-escalation trial of Adjuvant S-PBI (22.5, 25, 27.5, and 30Gy in 1 fraction)	March 2016	75
**Yonsei University**<br><br>(NCT03568981)	Yong Bae Kim, MD PhD	Observational cohort of adjuvant 5-fraction S-PBI and WBI	April 2018	200
**University of Alabama**<br><br>**RAD 1802**<br><br>(NCT03643861)	Drexell Hunter Boggs, MD	Trial of adjuvant 30Gy/5Fx via LINAC based S-PBI	Not yet recruiting	20
**University of Texas Southwestern, University of Maryland**<br><br>(NCT03581136)	Asal Rahimi, MD	Phase II trial of adjuvant 40Gy/5Fx preoperative S-PBI via GammaPod to CTV (1cm expansion) and 30Gy/5Fx to PTV (0.3cm expansion)	March 2019	40

Table 2. Ongoing adjuvant stereotactic partial breast irradiation (adjuvant S-PBI) trials.

2.0 STUDY OBJECTIVES

2.1 Primary Objectives

2.1.1 The primary objective is to escalate the dose of 1 fraction stereotactic partial breast radiotherapy utilizing the MR Linac, Gammapod or Cyberknife system to an ablative dose in the pre-operative setting to the primary tumor without exceeding the maximum tolerated dose in patients with early stage breast cancer.

2.2 Secondary Objectives

2.2.1 Local Control

2.2.2 Acute Toxicity (90 Days)

2.2.3 Late Toxicity (24 months)

2.2.4 Rates of Surgical Morbidity

2.2.5 Pathologic Complete Response Rates

2.2.6 Patient and Physician Cosmesis Outcomes

2.2.7 Distant Disease-Free Survival

2.3 Exploratory Objectives

2.3.1 <u>Radiomics on MRI</u>
Through extracting and analyzing a large number of features from medical imaging, radiomics has shown promising results in treatment outcome prediction for many diseases including breast cancer (45-50). UTSW physics group has developed several new radiomic approaches and radiomic features, such as a multi-objective radiomics model (51) and a new radiomic "Shell" feature (52). As an exploratory end point for this trial, we will explore the application radiomics using pre-treatment MRI, treatment parameters and clinical characteristics as input to predict pathological response of radiation therapy (XRT) based on pathology report of surgical tissues and local recurrence.

2.3.2 <u>Optional Translational correlates using blood samples and tissue samples from core biopsies and surgical tissue post-radiation</u>

- Primary tumor biopsies (pending volume of tissue)
  - Expression of PD-L1, PD-L2, PD1, CD3, CD68, CD8, Ki67.
  - RNAseq analyses including RNA profiling.
  - Whole exome sequencing to determine mutational burden and neoantigen detection.
  - T-cell receptor sequencing to determine intratumoral TCR diversity.
  - IHC to quantify immune populations.
- Blood (both Serum and PBMC)
  - T-cell receptor sequencing to determine tumor-specific TCR diversity, expansion and contraction.
  - Analysis of antibody response (both quantitative antibody titer and qualitative antibody binding and functionality).
  - Study of T-cell composition and functioning.
  - Identification of circulating antigen, DNA or tumor cells.

STU2019-1183, Rahimi, FormA-Protocol, Mod_15, 11-01-23 (7)

- o Analysis of cytokine response.
- o Analysis of blood cell composition using flow cytometry or mass cytometry.
- o Analysis of inflammatory status

2.4 Endpoints

The primary endpoint of the phase I portion is to either reach the maximum tolerated dose (MTD) or a dose of 38 Gy (whichever comes first) by escalating the dose of S-PBI toward the tumorcidal dose of 38 Gy in 1 fraction. Patients will be treated in cohorts of seven to fifteen. Toxicity will be graded using the NCI Common Toxicity Criteria for Adverse Events (CTCAE) v. 5.0. ***A dose- limiting toxicity (DLT) is a grade 3 toxicity deemed definitely related to treatment in the following categories: skin, rib bone (fracture), pulmonary (radiation pneumonitis), or neurological (injury to intercostal or brachial plexus nerves) or any grade 4 or 5 toxicity deemed definitely attributed to the therapy.*** All reported DLTs will be verified by study chair, data and safety monitoring committee, and, as appropriate, independent review before final determination that a DLT has in fact occurred. Doses will be escalated an additional 3.5-4 Gy per treatment. The phase I portion of the study will be completed when either of the following events occur: 1) the MTD for a cohort is reached or 2) when the highest protocol dose of 38 Gy, which the investigators have determined to be likely efficacious in controlling gross disease, is achieved and tolerated.

3.0 SUBJECT ELIGIBILITY

Eligibility waivers are not recommended; however, if warranted, prior approvals are required per Section 11.6.1. Subjects must meet all of the inclusion and exclusion criteria to be registered to the study. Study treatment may not begin until a subject is registered. Once registered, a subject is still required to meet all inclusion and exclusion criteria on the first day of treatment, prior to treatment.

3.1 Inclusion Criteria

1. Invasive epithelial (ductal, medullary, lobular, papillary, mucinous (colloid), or tubular) histologies of the breast 3 cm or less(T1-T2cN0) in women who have not undergone surgery or neoadjuvant endocrine or chemotherapy for current breast cancer diagnosis
2. Tumor must not involve the overlying skin based on imaging evaluation and/or clinical exam
3. Age >/= 18 years old and female
4. Greatest Tumor dimension is 3cm or less based on US. MRI measurements can be included only if performed BEFORE the biopsy
5. Tumor must be unifocal
6. The tumor must be visible on CT scan and/or preferably marked with clip(s) in tumor
7. Patients must undergo an MRI for work up to aid in tumor delineation and to rule out additional foci of disease. If additional foci of disease are present, they need to have a negative biopsy to proceed with treatment. If patient cannot have MRI, contrast enhanced digital mammography (CEDM) is allowed in place of MRI.
8. Clinically and radiographically node negative on ultrasound of the axilla or MRI
9. Estrogen receptor positive or Progesterone receptor positive and Her2neu negative
10. Ability to understand and the willingness to sign a written informed consent.
11. Women of child-bearing potential must agree to use adequate contraception (hormonal or barrier method of birth control) prior to the start of study and for the duration of radiation therapy. Should a woman become

STU2019-1183, Rahimi, FormA-Protocol, Mod_15, 11-01-23 (7)

pregnant or suspect she is pregnant while participating in this study, she should inform her treating physician immediately

A female of child-bearing potential is any woman (regardless of sexual orientation, having undergone a tubal ligation, or remaining celibate by choice) who meets the following criteria:

- Has not undergone a hysterectomy or bilateral oophorectomy; or
- Has not been naturally postmenopausal for at least 12 consecutive months (i.e., has had menses at any time in the preceding 12 consecutive months

3.2 Exclusion Criteria

1. Multi-centric disease
2. Prior RT to the involved breast
3. Tumor size >3cm
4. Uncontrolled intercurrent illness including, but not limited to, ongoing or active infection, symptomatic congestive heart failure, unstable angina pectoris, cardiac arrhythmia, or psychiatric illness/social situations that, in the opinion of the investigator, would limit compliance with study requirements
5. Patients who are pregnant or lactating due to the potential exposure to the fetus to radiation therapy and unknown effects of radiation therapy to lactating females
6. Patients unable to have an MRI or contrast enhanced digital mammography (CEDM)
7. Prior ipsilateral breast cancer
8. Tumor less than 5mm from the skin surface on clinical exam and/or radiographic imaging
9. Patients with active Lupus or scleroderma

4.0 TREATMENT PLAN

4.1 Treatment Dosage and Administration

The term “stereotactic” for the purposes of this protocol implies the targeting, planning, and directing of therapy using beams of radiation along any trajectory in 3-D space guided by one or several fiducials of known 3-D coordinates. This differs from conventional radiation therapy in which therapy is directed toward skin marks or bony landmarks and assumed to correlate to the actual tumor target based on a historical simulation. It should be understood that SBRT has become a treatment that is well beyond just stereotactic targeting. Indeed SBRT is mostly about high dose per fraction, accounting properly for errors including motion, careful construction of dosimetry that compacts high dose into the tumor and not normal tissues, and extra careful treatment conduct. This protocol will require treatments to be conducted with the use of the MR Linac, Cyberknife system® utilizing its techniques of continuous image guidance and targeting corrections for quality assurance during treatment, or the Gammapod which utilizes a vacuum assisted breast cup for immobilization.

<u>Technical Factors</u>
The Cyberknife system® with its 6 MV linear accelerator will be required for radiation delivery by the protocol, MR Linac, or the Gammapod which delivers radiation with cobalt sources. Other treatment delivery options will not be considered.

4.1.1 All patients in each dose cohort will be treated as a single group for dose escalation. The starting dose for the dose escalation portion will be 30 **Gy. Subsequent cohorts of patients will receive an additional 3.5-4 Gy per treatment. If significant toxicity is encountered at the starting dose, a de-**

escalation will occur (step -1) to 26.5 Gy. If at the time of this trial opening, we have completed our ongoing adjuvant single fraction stereotactic partial breast irradiation trial dose cohort 30 Gy, and have deemed its safe, we will skip the 30 Gy arm and start at 34 Gy for this trial. Based on modeling from the universal survival curve, 38 Gy in 1 fraction is roughly biologically equivalent to the dose of 70 Gy which is the dose commonly used to treat gross disease.

Level	Dose	No. of Patients
-1	26.5 Gy	7-15
1	30 Gy	7-15
2	34 Gy	7-15
3	38 Gy	7-15

Prescription will be minimum of 27 Gy to the PTV for all dose cohorts, and either 30 Gy, 34 Gy, or 38 Gy to the GTV/CTV per appropriate dose cohort.

Minimum waiting periods will be assigned between each dose cohort to evaluate toxicity. The phase I study will be completed when dose limiting toxicity is reached or when the highest protocol dose of 38 Gy, which the investigators have determined to be likely efficacious in controlling gross disease, is achieved and tolerated.

At the time of writing this protocol, the current single fraction trial being tested in the adjuvant setting for early stage breast cancer is 30 Gy in 1 fraction at UTSW (clinical trials.gov number NCT02685332). If we meet our accrual goal and waiting period without a dose limiting toxicity by the time this protocol is ready to go live, we will start our dose for this trial at 34 Gy rather than 30 Gy.

Treatment can be done on MR Linac, the Gammapod or Cyberknife.

<u>Dose Escalation</u>
Dose escalation on the phase I portion of this study should not occur until a sufficient waiting period has occurred after patients have been treated. A period of 90 days must pass in order to assess toxicity. If 90 days have elapsed without DLT in each of the first seven (7) patients enrolled to a specific dose level, then dose escalation to the next level may proceed. Patients will continue to be enrolled to each dose level (up to a maximum of 15 patients) with ongoing assessment of those reaching 90 day follow-up so long as either criteria for defining the MTD or criteria for further dose escalation is not reached. If fifteen patients are enrolled to a given dose level yet criteria for adequate follow-up are not reached in a representative sample of patients, further enrollment to the protocol will be suspended until adequate follow-up is reached. The phase I portion of this study will be completed when either of the following events occurs: 1) the MTD for a given cohort is reached or 2) when the highest protocol dose level is completed and tolerated

4.1.2 <u>Cyberknife localization, simulation, immobilization</u>

Patients will be positioned supine in a stable position capable of allowing accurate reproducibility of the target position from treatment to treatment. Positions uncomfortable for the patient should be avoided so as to prevent uncontrolled movement during treatments. A variety of immobilization systems may be utilized including Vac Loc® bags, stereotactic frames that surround the patient on three

sides, and large rigid pillows. All positioning systems must be validated and accredited by the Study Committee (Principal Investigator and Institutional PIs) prior to enrolling or treating patients on this trial. Patient immobilization must be reliable enough to insure that the Clinical Target Volume (CTV) does not deviate beyond the confines of the Planning Treatment Volume (PTV)

Daily localization will also be achieved using a more direct method of localization of the target than conventional treatment (i.e., one that uses skin and boney landmarks solely as a surrogate to the target position). Fiducials are used help ensure accurate daily target localization prior to treatment. Special considerations must be made to account for the effect of internal organ motion (i.e., breathing, etc.) on target positioning and reproducibility. This will be achieved using the fiducial tracking, Excite® tracking, and/or Synchrony® real-time tracking associated with the Cyberknife® per established system standards. Paired low-energy X-ray sources generate orthogonal X-ray images to determine the location of fiducial markers or bony landmarks continuously throughout treatment

Computed Tomography will be the primary image platform for targeting and treatment planning. The planning CT scans must allow simultaneous view of the patient anatomy and fiducials used for targeting. Treatment planning images should be performed in the treatment position using the methods described above for patient immobilization. Axial acquisitions with gantry 0 degrees will be required with spacing ≤ 3.0 mm between scans. The CT scan should start at or above the mandible and extend several cm below the inframammary fold (including the entire lung). Images will be transferred to the treatment planning computers via direct lines, disc, or tape.

4.1.3 <u>Gammapod breast cup placement, simulation, immobilization</u>

The breast immobilization cup device **(BCID)** will be supplied by Xcision. With the breast hanging prone (patient bent over at the waist), the patient's breast will be measured to determine the size of the inner cup. Once the appropriate cup site has been selected any gaps > 1 cm will be filled with a silicone filler attached to the inner cup. The inner cup will then be fitted into the appropriate pink silicone flange as chosen by the physician. The appropriate outer cup with built in fiducial system will be selected (S, M, or L) and attached to the inner cup and flange. Medical adhesive spray will be applied to the flange.

With the immobilization device complete, the breast will then be placed into the the cup. A slight negative pressure will then be applied, fixed at approximately 150 mm Hg. The breast will then be immobilized in the cup. If the seal cannot be created with the vacuum pump, additional adhesive may be utilized and or tape applied. If the seal cannot still not be maintained, then the inner/outer cup sizes and/or silicone filler may be changed. This may occur several times until either the physician deems the attempts futile, the patient's skin becomes irritated from the adhesive and/or patient preference.

<u>CT-based treatment planning for the GammaPod Treatment</u>
Once the breast is properly immobilized, the patient will step onto the image loader and be rotated into the prone position. The breast cup will then be locked to the image loader table. Patients will then undergo CT simulation in the prone position. The CT scan will begin at the thyroid notch and extend to below the infra-mammary fold (including the entire lung) with a slice thickness of 1.0 mm throughout the breast.

If at the time of CT simulation the tumor is found to be located > 5 mm above the table top (as seen in Figure 1), If the tumor is located above this area, the patient

STU2019-1183, Rahimi, FormA-Protocol, Mod_15, 11-01-23 (7)

will be deemed ineligible for the gammapod per treating physician and should proceed with cyberknife or MR LINAC treatment if possible. (Description of Gammapod breast cup placement, simulation, immobilization and figure below from Dr. Liz Nichols from University of Maryland)

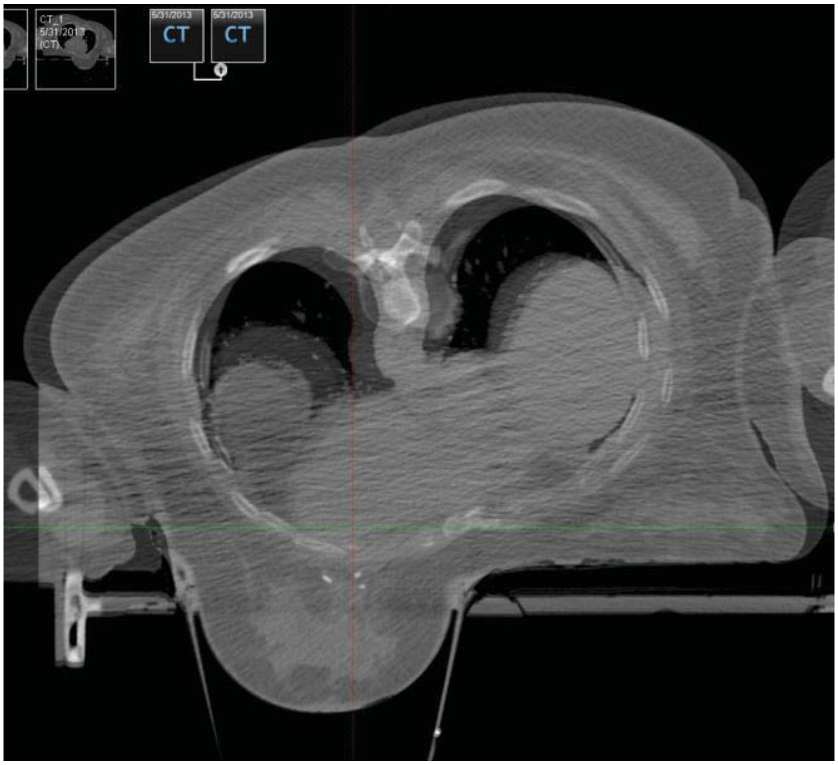

Figure 1: the line where you see artifact indicates the line from which tumor should not extend > 5 mm.

MR-LINAC Simulation and Immobilization

The MR-LINAC allows practitioners to obtain MRI images at simulation and use them during planning and delivery. Contrast will be given and functional imaging will also be available. Patients will be simulated in supine position, and breast immobilization may be used per treating physician.

4.1.4 GTV, CTV, PTV Target Volumes for both Cyberknife and Gammapod

Target Volumes for Treatment

Gross Target volume (GTV): this will be defined as the tumor as delineated on CT images and the location of the biopsy clip. Care should be taken by the radiation oncologist to change the windowing to optimize the contouring windows. Information from the breast MRI should also be utilized to assist in contouring. If the RO is unsure of the contours, it is recommended that the institutional breast radiologist be available for discussion/assistance.

Clinical Target volume (CTV): the GTV will be equal to CTV as we are using a SBRT technique and the surrounding tissue will inherently be getting high doses of radiation from the dose fall off.

Planning Target volume (PTV): the PTV will be a recommended concentric expansion of the CTV of 3-10 mm excluded from the skin and chest wall. As imaging techniques improve and/or dose is escalated, the PTV margin may be reduced at the discretion of the treating physician. The PTV margin will take into account set up error, and imaging modality used for planning (ie; CT scan versus MRI for planning)

GTV, CTV, and PTV for MR LINAC treatments

Gross Tumor Volume (GTV)- this will be defined as the tumor delineated on the MR images/CEM and/or contrast enhanced CT scan

Clinical Target Volume (CTV)- the GTV will be equal to CTV as we are using a SBRT technique and the surrounding tissue will inherently be getting high doses of radiation from the dose fall off.

Planning Target Volume (PTV): The PTV will be a concentric expansion of the CTV of anywhere between 3mm-10mm but recommended to be 5mm excluded from the skin and chest wall.

4.1.5 Radiation Planning and Target Goals for MR LINAC, Gammapod and Cyberknife:

- 95% of the PTV should receive a minimum of 27 Gy and 99% of the target volume (GTV) receives a minimum of 93% of the prescription dose. Minor deviation if 90% of PTV receives 27 Gy. All efforts should be made to meet the skin constraint even if PTV coverage will be compromised. In this situation, would prioritize skin, brachial plexus, and spinal cord constraint over PTV coverage and this is acceptable and not a deviation, and will not be deemed a protocol violation if PTV coverage not met in order to meet these OAR constraints. Efforts should be made to keep the PTV volume coverage to a minimum dose of 27 Gy to 95% of volume. In the situation when the GTV is close to skin, prioritize the skin constraint over GTV coverage.
- Max hot spot < 130% of the prescription dose is recommended
- Prescription will be either 30 Gy, 34 Gy, or 38 Gy to the GTV/CTV per appropriate dose cohort.

Critical Organ Dose-Volume Limits

The following table lists maximum dose limits to a point or volume within several critical organs. These are absolute limits, and treatment delivery that exceeds these limits will constitute a major protocol violation

In order to verify each of these limits, the organs must be contoured such that appropriate dose volume histograms can be generated. Instruction for the contouring of these organs will follow below. When all constraints cannot be met the following priority should be met:

Priority 1: Spinal cord, brachial plexus, skin, trachea, large and small bronchus
Priority 2: Cover PTV
Priority 3: Heart, rib, thyroid, lung

**Serial Tissue**	Volume	**Volume Max (Gy)**	**Max Point Dose (Gy)****	**Endpoint (≥Grade 3)**
Spinal Cord and medulla	<0.35 cc	10 Gy	14 Gy	myelitis
Brachial Plexus	<3 cc	13.6 Gy	16.4 Gy	neuropathy

Heart	<15 cc	16 Gy	22 Gy	pericarditis
Trachea and Large Bronchus* (Ck only)	<4 cc	17.4 Gy	20.2 Gy	stenosis/fistula
Bronchus-smaller airways (CK only)	<0.5 cc	12.4 Gy	13.3 Gy	stenosis with atelectasis
Rib	<5 cc	28 Gy	33 Gy	Pain or fracture
Skin	<10 cc	25.5 Gy	27.5 Gy	ulceration
**Parallel Tissue**	Critical Volume (cc)	**Critical Volume Dose Max (Gy)**		**Endpoint (≥Grade 3)**
Thyroid gland (CK only)	5 cc	15 Gy		Hypothyroid
Ipsilateral Breast	<40% of whole breast reference volume		>/= 50% prescribed dose	Cosmesis
Ipsilateral Breast	<20% of whole breast reference volume		>/= 100% prescribed dose	Cosmesis
Mean Heart dose				Please record value
Lung (Right & Left) CK only	1500 cc	7 Gy		Basic Lung Function
Lung (Right & Left) CK only<br>Chest Wall	1000 cc<br><br><30cc	7.6 Gy<br><br>30 Gy	V-8Gy <37%	Pneumonitis<br><br>Chest wall pain

***Avoid circumferential irradiation**
**** "point" defined as 0.035cc or less**

<u>Dosimetry Compliance</u>
Exceeding dosimetric limits by more than 2.5% constitutes a minor protocol violation. Exceeding dosimetric limits by more than 5% constitutes a major protocol violation.

4.1.6 <u>Cyberknife and MR LINAC Treatment Planning Goals</u>

1) <u>Normalization</u>
The treatment plan should be normalized such that 100% corresponds to the maximum point dose in the irradiation volume and is required to be within the CTV volume.

2) <u>Prescription Isodose Surface Coverage</u>
The ideal prescription isodose surface will be chosen such that 95% of the target volume (PTV) is covered by at least 27Gy and 99% of the target volume (GTV) receives a minimum of 93% of the prescription dose. Minor deviation prescription isodose coverage in the instance that other normal structure constraints need to be met, is 90% of the target volume (PTV) covered by 27Gy. All efforts should be made to meet the skin constraint even if PTV coverage will be compromised. In this situation, would prioritize skin, brachial plexus, and spinal cord constraint over PTV coverage and this is acceptable, and will not be deemed a protocol violation if PTV coverage not met in order to meet these OAR constraints. Efforts should be made to keep the PTV volume coverage to a minimum of dose of 27 Gy to 95% of volume.

In the situation when the GTV is close to skin, prioritize the skin constraint over GTV coverage.

3) Target Dose Heterogeneity
The prescription isodose surface selected in number 2 (above) must be ≥ 50% of the dose at the maximal dose point or normalization point.

4) High Dose Spillage
   a) *Location*
   Any dose greater than 105% of the prescription dose should occur primarily within the CTV itself and not within the normal tissues outside of the CTV. Therefore, the cumulative volume of all tissue outside of the CTV receiving a dose greater than 105% of prescription dose should be no more than 15% of the CTV volume.
   b) Volume
   Conformality of CTV coverage will be judged such that the ratio of the volume of the prescription isodose meeting criteria 1) through 4) to the volume of the CTV is ideally less than 1.3.

5) Respect all critical organ dose-volume limits listed in Section below

6) Reduction of dose to ipsilateral breast: Based on the previously mentioned studies, cosmesis is dependent on dose to uninvolved ipsilateral normal breast. Thus, there will be strict guidelines reducing the dose to the whole breast volume so that <40% of the whole breast reference volume receives ≥ 50% of the prescribed dose and < 20% of the whole breast reference volume receives ≥ 100% of the prescribed dose.

7) Reduction of dose to the contralateral breast: The contralateral breast should be contoured and should receive <3% of the prescribed dose to any point.

4.1.7 Contouring of normal Structures for Cyberknife and MR LINAC

Contouring of Normal Tissue Structures

Heart
The heart should be contoured beginning just below the level in which the pulmonary trunk branches into the left and right pulmonary arteries (PA). Above the PA, none of the heart's 4 chambers are present. All the mediastinal tissue below this level should be contoured, including the great vessels (ascending and descending aorta, inferior vena cava). The heart should be contoured on every contiguous slice thereafter to its inferior most extent near the diaphragm. If one can identify the esophagus, this structure should be excluded. One need not include pericardial fat, if present. Contouring along the pericardium itself, when visible, is appropriate.

Lungs
Both the right and left lungs should be contoured as separate structures. Contouring should be carried out using pulmonary windows.

Skin
The skin will be defined as the outer 0.5 cm of the body surface. As such it is a rind of uniform thickness (0.5 cm) which envelopes the entire body in the axial planes. The cranial and caudal surface of the superior and inferior limits of the

planning CT should not be contoured as skin unless skin is actually present in these locations (e.g., the scalp on the top of the head).

Ipsilateral and contralateral breast
Each breast is contoured separately. The entire breast mound should be contoured including glandular tissue, intervening fibrous and adipose tissues, subcutaneous fat, and overlying skin and nipple to begin medially adjacent to the sternum, laterally at the mid axillary line or reflection of breast (whichever is more extreme), superiorly below the clavicle or reflection of breast (whichever is more extreme), inferiorly at the inframammory fold or reflection of breast (whichever is more extreme), and centrally at the chest wall/breast interface (not including pectoralis or other deep muscles).

Brachial Plexus
The defined ipsilateral brachial plexus originates from the spinal nerves exiting the neuroforamina on the involved side from around C5 to T2. However, for the purposes of this protocol, only the major trunks of the brachial plexus will be contoured using the subclavian and axillary vessels as a surrogate for identifying the location of the brachial plexus. This neurovascular complex will be contoured starting proximally at the bifurcation of the brachiocephalic trunk into the jugular/subclavian veins (or carotid/subclavian arteries) and following along the route of the subclavian vein to the axillary vein ending after the neurovascular structures cross the second rib.

Ribs
Only ribs within 4 cm of the treatment volume need to be contoured.

4.1.8 Contouring of normal Structures for Gammapod

Organs at Risk: Organ at risk doses are unable to be calculated in real-time in the treatment planning system for Gammapod. As such, certain isodose lines will be displayed. The physician and physicist will review these isodose lines and their proximity to specific organs including but not limited to the lung, heart, chest wall and skin. Following completion of treatment, dose-volume histogram analysis will occur. The same structures as above in the section 4.1.7 can be done after treatment. If a dose constraint (listed below) is not met, this will be recorded as a deviation. The lung constraints may not be able to be obtained, as for Gammapod treatment it is hard to get the entire lung in view. All plans will undergo central review.

However, during treatment in real time the following can serve as a guide for evaluation of a Gammapod plan in real time.

Heart: the heart will be contoured following treatment as in section 4.1.7. During the treatment planning process, the isodose line representing the 31% isodose line should be displayed. This isodose line should not touch the surface of the heart/pericardium. While in many circumstances in a dose escalation study, the OAR doses would increase with each dose cohort,we will also calculate and record the mean heart dose as this parameter has been found to have significance to the risk of heart disease for women with breast cancer.

Ipsilateral lung: the ipsilateral lung will be contoured following treatment and will include the entire lung within the CT scan. Due to the focused nature of the treatment and the requirement to perform CT simulation with 1 mm slices, the entire lung from apex through the diaphragm. During the treatment planning process, the isodose line representing the 31% prescription isodose line should be displayed. This isodose line should not touch the surface of the lung. As we

will not specifically be scanning through the entire lung, a normal lung DVH cannot be calculated.

Ipsilateral chest wall: the ipsilateral chest wall will include the pectoralis muscles, intercostal muscles and the ribs. As with the other OARs, this will not be contoured during the treatment planning process but will be evaluated afterwards. During the treatment planning process, the 30% isodose line will be displayed. This should not touch the surface of the muscle/rib. In the post-treatment evaluation, the dose constraint of 30 Gy < 30 cm^3 should be evaluated and recorded. This metric has been shown to be an important metric for lung SBRT and correlates with a risk of chest wall pain. Chest wall pain is not common during breast cancer radiation and treatment. As such we want to adopt a similar treatment parameter.

Skin: the skin will be defined as the first 5 mm of skin from the surface into the breast. The TPS will auto contour this from the surface of the inner cup. However, the physician and physicist will need to review this and edit it as the system does not have the ability to auto-contour the silicone bolus, thus this will have to be edited as appropriate. The max dose to the skin surface (true surface) is calculated in real time from the TPS.

4.1.9 Radiation Therapy Quality Assurance Reviews

The principal Investigator or her designee along with a medical physicist, will perform an RT Quality Assurance Review after complete data for the first 20 cases enrolled has been received at the University of Texas Southwestern. They will perform the next review after complete data for the next 20 cases enrolled has been received at the University of Texas Southwestern. The final cases will be reviewed within 3 months after this study has reached the target accrual or as soon as complete data for all cases enrolled has been received, whichever occurs first.

4.1.10 Potential Risks

Potential Risks/Discomfort: The first risk of participation in the study is that patients may be inappropriately selected for this treatment modality since surgery is not performed first. The primary breast tumor could be larger (or smaller) than what the pre-treatment imaging shows. The patient could have lymph node involvement not detected in their work up but only detected at the time of surgery. To decrease this risk, patients will undergo rigorous screening and work up. Only patients with small, ER+ tumors will be enrolled in the trial. These have a lower risk of lymph node involvement. In the event that the patient's stage or tumor size is higher or larger than what is expected at the time of pathology, further treatment parameters have been recommended within the protocol to ensure that patients receive appropriate treatment.

The second risk in this study is a risk of increased surgical morbidity. Pre-operative partial breast trials to date show rates of surgical morbidity that are equivalent or in line with the standard of care. There have been minimal wound healing complications. Seromas have been noted, however, the incidence of these in the currently available literature is no different than standard series.

Finally, there is a potential risk of a worse cosmetic outcome in the short or long term. While there are no convincing data to suggest such an outcome based on the currently available literature, there has not been 10 year follow up with this treatment paradigm.

STU2019-1183, Rahimi, FormA-Protocol, Mod_15, 11-01-23 (7)

4.2 Toxicities and Dosing Delays/Dose Modifications

Any subject who receives treatment on this protocol will be evaluable for toxicity. Each patient will be assessed for the development of toxicity according to the Time and Events table in Section 5.4. Toxicity will be assessed according to the NCI Common Toxicity Criteria for Adverse Events (CTCAE), version 5.0. Dose adjustments should be made according to the system showing the greatest degree of toxicity.

4.3 Concomitant Medications/Treatments

4.3.1 Systemic and Adjuvant Surgery

<u>Systemic Therapy</u>
Use of endocrine therapy after pre-operative radiation should be started within 21 days after pre-operative radiation, but it is encouraged to start the day after the post-radiation biopsy. Typically, for estrogen/progesterone positive patients, many will start endocrine therapy as they are waiting for surgery. Oncotype or Mammaprint studies should be done on the biopsy specimen, if possible, prior to delivery of radiation if there is adequate tissue. If not, patient may require another biopsy to obtain this tissue and this will be at the discretion of the medical oncologist

<u>Surgery:</u> Surgery can be done anytime from 8 to 52 weeks at the discretion of the surgeon. Sentinel lymph node biopsy can be done at time of lumpectomy or if patient prefers mastectomy.

Sentinel lymph node mapping: in the previous University of Maryland pre-operative study there were no instances of failed sentinel lymph node mapping on the 25 patients treated on the trial. Based on this experience, we have no reason to think that there should be an increase in failed mappings with the pre-operative radiation treatment. A peri-tumoral, intra-dermal or peri-areolar injection are allowed based on the discretion of the breast surgeon.

Sentinel lymph node mapping exclusions: In the elderly population (defined differently in by different trials and also based on medical comorbidities), a sentinel lymph node procedure is often not performed on low-risk patients. In this trial, we will not require women 70+ to have a sentinel lymph node mapping if it is deemed unnecessary by their treatment team. This must be documented in the patient's medical record.

<u>Clinical Scenarios requiring Guidance:</u>
<u>Positive margin:</u> Patients should undergo re-excision per normal re-excision parameters

<u>Positive sentinel lymph node (macrometastasis/micrometastasis):</u> per Z11 if a patient has 1-2 positive sentinel LN, no further axillary dissection is required. If 3 or more sentinel LN are positive, axillary dissection should be considered unless the overall percentage of positive nodes is <20%. If a patient has a positive sentinel LN, whole breast radiation therapy should be delivered following surgical resection. In this scenario, this study will recommend conventional fractionation to the breast with an option to also treat the regional lymphatcs as opposed to a hypofractionated 'short course' whole breast treatment alone. The rationale for this is that previous groups have identified a poorer cosmetic outcome when combining whole breast radiation therapy with pre-operative treatment. Therefore, we feel that it would be prudent to use conventional fractionated regimen when combining with pre-op radiation. A dose of 45 Gy in 1.8 Gy

fractions will be used for whole breast +/- a supraclavicular field for radiation therapy.

Isolated tumor cells in sentinel lymph node: no further axillary surgery. This specific scenario will be documented and it will be up to the discretion of the treating physician if whole breast radiation is required as above.

Microcsopic ECE: if a node has microscopic ECE this will be treated as per bullet point 2 above. Macroscopic ECE should not be seen as these patients would be ineligible for treatment.

Patient refuses surgery: there may be instances where patients refuse surgical resection after receiving pre-op radiation. This is not the intent of the trial, however, the decision to undergo surgery is at the patient's discretion. In this scenario, the clinical team should document attempts to discuss with the patient. If ultimately the patient elects to not undergo surgery, the PI should be notified and a note to file will be placed in the patient's permanent medical record documenting attempts to discuss this with the patient and documenting the patient's refusal. This should also be sent to the UTSW radiation oncology study team.

4.3.2 Medications

Premedication

Analgesic premedication, such as Tylenol, and anti-anxiety medications such as Ativan, may be administered prior to therapy and as needed every 6 hours, to avoid patient discomfort.

Supportive Medicines

Consider Biafin during treatment period to reduce skin irritation. Consider analgesics such as Tylenol, Ibuprofen, or other NSAIDs for pain resulting from inflammation.

4.4 Duration of Therapy

Patients will receive one fraction of radiation.

4.5 Duration of Follow Up

Subjects will be followed for **5 years**. Subjects removed from treatment for unacceptable adverse events will be followed until resolution or stabilization of the adverse event. Patients will be followed 1, 3, 6, 12, 24, and 36 months after radiation. See Section 5.4 Time and Events Table for follow-up visit windows.

4.6 Removal of Subjects from Protocol Therapy

Subjects will be removed from therapy when any of the criteria listed in Section 5.5 apply. Notify the Principal Investigator, and document the reason for treatment discontinuation and the date of discontinuation. The subject should be followed-up per protocol.

4.7 Subject Replacement

If patient does not receive single fraction radiation therapy, that patient can be replaced in the study.

5.0 STUDY PROCEDURES

5.1 Screening/Baseline Procedures

Assessments performed exclusively to determine eligibility for this study will be done only after obtaining informed consent. Assessments performed for clinical indications (not exclusively to determine study eligibility) may be used for baseline values even if the studies were done before informed consent was obtained.

All screening procedures must be performed within 180 days prior to registration into the study unless otherwise stated. The screening procedures include:

5.1.1 Informed Consent

5.1.2 Medical history

Complete medical and surgical history, breast and lymph node examination and palpable size of tumor. Any ongoing signs/symptoms present during Screening should be assessed and graded per CTCAE, and followed as per section 7

5.1.3 Demographics

Age, gender, race, ethnicity

5.1.4 Review subject eligibility criteria

5.1.5 Review previous and concomitant medications

5.1.6 Physical exam including vital signs

Vital signs (temperature, pulse, respirations, blood pressure)

5.1.7 Performance status

Performance status evaluated prior to study entry according to Appendix I

5.1.8 Adverse event assessment

Baseline adverse events will be assessed. See section 7 for Adverse Event monitoring and reporting.

5.1.9 Breast Assessment/Examination

5.1.10 Breast Imaging

Mammogram and Ultrasound of breast and axilla prior to treatment

5.1.11 Blood draw and tumor biopsy at baseline and surgical specimen for correlative studies

Peripheral blood samples will be collected within 30 days prior to radiation therapy treatment, 4 weeks following completion of the radiation therapy delivery, and 4 weeks after surgery. Samples will be stored per human use and institutional protocols and guidelines. At the present time, we do not have funding for further analyses of these samples. However, we do anticipate receiving funding to undertake such correlative studies. UTSW has agreed to store and house blood and tissue samples.

We will also attempt to obtain tissue from the tumor biopsies per Section 5.4 of Time and Events table. The tumor samples from biopsies prior to radiation treatment and within 14 days after radiation treatment are optional. The tumor

sample collected at time of surgery is mandatory and will be collected for each patient. See Section 9.0 for correlative study details.

5.1.12 Photographs

Photographs of the patient's breasts should be taken prior to radiation therapy. The first photo should be a close-up encompassing only the breast to be treated at a 45 degree oblique angle with arms elevated over the patient's head. The second photo should be a straight frontal view of both breasts taken in either a standing or seated position with the patient's hands on her hips, excluding her face. Label each slide or photograph with the date and the patient case number. See section 6.2 for photo evaluation criteria.

5.1.13 Pregnancy test (for women of childbearing potential)

See section 3.1.11 for definition.

5.1.14 Breast MRI

Baseline Breast MRI will be required to determine eligibility, however if patient is unable to have an MRI, contrast enhanced digital mammography (CEDM) is allowed in place of the MRI. This is per standard of care for a patient with a new diagnosis. **An additional optional breast MRI is highly recommended and may be completed at least 4 weeks after radiation.**

The research being done with serial MRI scans is to determine whether they can help assess response in women receiving Gammapod, MR Linac, or Cyberknife therapy to monitor treatment changes prior to surgery. **MRI scans will be performed at two time points before and after radiotherapy to determine treatment response.** Each imaging exam will last approximately one hour. It is recommended but not mandatory. Breast MRI can be performed on either a 1.5T or 3.0 T scanner; it is preferred that all MRI exams for a given participant be performed using the same MRI field strength.

If possible, the MRI scanner make/model (*e.g.,* GE, Siemens, Philips, Toshiba), breast coil, contrast agent, and image acquisition protocol should also remain consistent for each MRI exam a participant receives at UTSW. This is not mandatory but highly recommended as some patients may get MRI at outside facilities.

Prior to each MRI exam, the participant will be asked to complete or update a metal screening form. An intravenous catheter will be inserted in the participant's arm or hand prior to the start of imaging. The imaging exam will be performed with the participant lying on her stomach.

The recommended but not mandatory image acquisition protocol will include a localization scan, a T2-weighted imaging sequence, a diffusion-weighted imaging sequence, and a T1-weighted contrast-enhanced imaging series**.** For contrast-enhanced imaging, Gadavist will be administered intravenously at a dose of 0.1 mmol/kg body weight followed by a saline flush. Contrast injection will begin simultaneously with the start of data acquisition.

MRI Time Points

Two MRIs will be performed for each participant at the following points:

- MRI1: (baseline) prior to the start of neoadjuvant treatment. Any additional lesions identified on MRI will be addressed per standard of patient care. If patient cannot have MRI, contrast enhanced digital mammography(CEDM) is allowed in place of MRI.

STU2019-1183, Rahimi, FormA-Protocol, Mod_15, 11-01-23 (7)

- MRI2 (optional but highly recommended): (*post Gammapod, MR Linac, or Cyberknife*) After MRI1, before surgical excision. The purpose of this optional scan is to test response to radiotherapy.

MRI post processing
Subtraction series of all dynamic post contrast sequences will be obtained. Maximum Intensity Projection (MIP), and ADC maps of the Diffusion weighted series will be obtained. DWI difference will be computed between baseline and post radiation therapy.

5.1.15 *Patient and Physician Cosmesis Forms (Appendix III and IV)*

Both patients and physicians will fill out patient cosmesis forms at baseline and per Section 5.4 time and events table.

5.1.16 Oncotype Testing

It is recommended that Oncotype or mammoprint testing be performed or considered on the biopsy specimen as the response of the tumor to radiation may alter Oncotype or Mammoprint testing results.

5.2 Procedures During Treatment

5.2.1 Baseline/Prior to Radiation Therapy

- Breast Assessment/Examination
- Breast Imaging (see section 5.1.10 for details)
- Breast MRI or contrast enhanced digital mammography(CEDM) (see section 5.1.14 for details)
- History and Physical Exam (including Performance Status)
- Baseline adverse event assessment
- Baseline breast digital pictures
- Patient and Physician Cosmesis Forms
- Optional blood samples for correlative studies within 30 days prior to radiation
- Tumor sample from diagnostic biopsy
- Optional Tumor Sample within 30 days prior to radiation.
- Pregnancy Test (if applicable)

5.2.2 Within 14 days (+2 days) after Radiation

- Optional tumor sample

5.2.2. 1 month after Radiation

- History and Physical Exam
- Breast Assessment/Examination
- Toxicity/Adverse event evaluation
- Optional Blood Samples
- Optional Breast MRI (at least 4 weeks after XRT and before surgery)

5.2.3 Surgical Procedure

Tumor Sample for Research at time of surgery

5.2.4 1 month after Surgery

Optional Blood Samples

5.2.5 3 months after Radiation

Toxicity/Adverse event evaluation (via phone call by study team member)

5.2.6 6 Months after Radiation

- History and Physical Exam
- Breast Assessment/Examination
- Toxicity/Adverse event evaluation

5.2.7 12 months after Radiation

- History and Physical Exam
- Breast Assessment/Examination
- Toxicity/Adverse event evaluation
- Breast Imaging

5.2.8 24 months after Radiation

- History and Physical Exam
- Breast Assessment/Examination
- Toxicity/Adverse event evaluation
- Breast Imaging

5.2.9 36 months after Radiation

- History and Physical Exam
- Breast Assessment/Examination
- Toxicity/Adverse event evaluation
- Breast Imaging
- Breast Digital Picture (see section 5.1.12 for details)
- Patient and Physician Cosmesis forms

5.3 Follow-up Procedures

Subject will be followed at 1 month post-radiation and 3, 6, 12, 24, and 36 months after completion of (or early withdrawal from) study treatment. Time points should follow the visit windows listed under Section 5.4 Time and Events table.

5.4 Time and Events Table

	Pre-XRT	Within 14 days after XRT	1 month after XRT[1]	Time of surgery	1 month after surgery[1]	3 months after XRT[1]	6 months after XRT[1]	12 month after XRT[2]	24 months after XRT[2]	36 months after XRT[2]
**Procedures**										
Informed Consent	X									
History and PE	X		X				X	X	X	X
Breast Assessment/ Examination	X		X				X	X	X	X
Performance Status	X									
Pregnancy Test	X									
Baseline AE Evaluation	X									
Toxicity/AE Evaluations (including DLT)			X			X[5]	X	X	X	X
Breast Imaging (Mammogram/ US, and or Ultrasound)	X							X	X	X
Breast MRI	X[4]		X[4]							
Breast Digital Picture	X[9]									X
Patient Cosmesis Forms (Appendix III)	X[10]									X
Physician Cosmesis Forms (Appendix IV)	X[10]									X
Optional Blood Samples	X[3]		X		X					
Tumor Sample	X[7]			X						
Optional Tumor Sample	X[8]	X[6]								

1 Follow-ups can be done within +/- 21 days of time points above and can be done via telehealth
2 Follow-ups can be done within +/- 60 days of time points above and can be done via telehealth
3 To be completed within 30 days prior to radiation
4 Baseline MRI or contrast enhanced digital mammography(CEDM) is required, Post-radiation MRI is optional but highly recommended and if done, post radiation MRI must be completed at least four weeks after XRT and before surgery.
5 Completed via phone evaluation by study team member
6 Optional tumor sample collection can be done within +2 days of time point above
7 Archival or diagnostic tumor samples
8 Optional tumor sample to be completed within 30 days prior to radiation
9 Should be taken prior to radiation therapy
10 Patient and Physician cosmesis can be completed any time after consent but prior to radiation treatment

5.5 Removal of Subjects from Study

Subjects can be taken off the study treatment and/or study at any time at their own request, or they may be withdrawn at the discretion of the investigator for safety, behavioral or administrative reasons. The reason(s) for discontinuation will be documented and may include:

5.5.1 Subject voluntarily withdraws from treatment (follow-up permitted);

5.5.2 Subject withdraws consent (termination of treatment and follow-up);

5.5.3 Subject is unable to comply with protocol requirements;

5.5.4 Subject demonstrates disease progression (unless continued treatment with study drug/treatment is deemed appropriate at the discretion of the investigator);

5.5.5 Subject experiences toxicity that makes continuation in the protocol unsafe;

5.5.6 Treating physician determines continuation on the study would not be in the subject’s best interest;

5.5.7 Subject becomes pregnant (pregnancy to be reported along same timelines as a serious adverse event);

5.5.8 Development of second malignancy (except for basal cell carcinoma or squamous cell carcinoma of the skin) that requires treatment, which would interfere with this study;

5.5.9 Lost to follow-up. If a research subject cannot be located to document survival after a period of 2 years, the subject may be considered “lost to follow-up.” All attempts to contact the subject during the two years must be documented

6.0 MEASUREMENT OF EFFECT

6.1 Antitumor Effect - Solid Tumors

6.1.1 Definitions

Evaluable for toxicity. All subjects will be evaluable for toxicity from the time of their first treatment with study therapy.

Evaluable for objective response. Only those subjects who have measurable disease present at baseline, have received radiation, and have had their disease re-evaluated will be considered evaluable for response. These subjects will have their response classified according to the definitions stated below. (Note: Subjects who exhibit objective disease progression prior to the end of cycle 1 will also be considered evaluable.)

Diagnosis of Breast Cancer Recurrence and Other Cancer Events

Ipsilateral in-breast recurrence: The definition of treatment failure is histologic evidence of recurrent carcinoma, either invasive or non-invasive (except LCIS) in the ipsilateral breast or chest wall. Clinical evidence of carcinoma by physical examination and/or mammograms will not be construed as evidence of treatment failure without biopsy proof but will be considered as suspicious for recurrence. Ipsilateral breast recurrences will be considered local (infield) if they occur within the prescription isodose volume; they will be considered peripheral if they occur between the prescription isodose volume and a volume 2 cm outside of the prescription isodose volume. Ipsilateral recurrences will be considered non-contiguous or extrafield if they are beyond the peripheral volume described above.

Regional recurrence:
Defined as the development of tumor in the ipsilateral mammary, ipsilateral supraclavicular, ipsilateral infraclavicular and/or ipsilateral axillary nodes, as well as the soft tissue of the ipsilateral axilla after operation.

Distant recurrence: Defined as evidence of tumor in any area of the body diagnosed with positive cytology, histologic biopsy, or radiologic evidence of metastatic disease.

Distant Disease-Free Interval: The distant disease-free interval will be measured from the date of registration until the date of first diagnosis of distant disease.

Recurrence free survival: Time from registration to first diagnosis a local, regional, or distant recurrence.

Overall Survival: The survival time will be measured from the date of accession to the date of death. All patients will be followed for survival. Every effort should be made to document the cause of death.

Disease-Specific Survival: will be measured from the date of study entry to the date of death due to breast cancer. The following will be considered as failure events in assessing disease specific survival:

Death certified as due to breast cancer.
Death from other causes with active malignancy.

Death due to complications of treatment, irrespective of the status of malignancy.
Death from other causes with previously documented relapse as defined above but inactive at the time of death will not be considered in disease-specific survival, but will be analyzed separately.

6.1.2 Methods for Pre-operative Evaluation of Measurable Disease

All measurements should be taken and recorded in metric notation using a ruler or calipers. All baseline evaluations should be performed as closely as possible to the beginning of treatment and not more than Response Criteria on MRI Response assessment to stereotactic radiotherapy will be performed using contrast-enhanced dynamic breast MRI (DCE-MRI) imaging.

Recommended MRI technique: (may not be possible for all MRI's as some may be done at ouside facilities)

Recomended MRI studies will be performed with the patients lying prone in a scanner using a dedicated multi-channel breast array coil (MRI Devices Corporation, Pewaukee, WI). The breast MRI protocol is attached and will comprise pre-and post contrast dynamic 3-dimensional fast spoiled-gradient echo image sets, as well as diffusion weighted image series (DWI). Images will be obtained during and following rapid intravenous bolus infusion of gadolinium contrast at a rate of 3 mL/sec with a power injector (Spectris Solaris MR Injector; Medrad, Warrendale, PA) with delayed postcontrast 3-dimensional fast spoiled-gradient echo images with fat suppression in both axial and sagittal planes obtained.

Imaging response assessment
i. The precontrast dynamic image set will be subtracted from the postcontrast sets, and time-intensity curves will be obtained from contrast-enhanced lesions with a hand-drawn region of interest (ROI) encompassing the lesion, with a

STU2019-1183, Rahimi, FormA-Protocol, Mod_15, 11-01-23 (7)

minimum ROI size of 3 × 3 pixels. The time-intensity curve demonstrating maximal change will be selected as representative for a lesion to perform pharmokokinetic analysis as needed. Further same measurements will be performed from peritumoral stroma (within 2 cm of the tumor margins) to measure the radiation effect on stroma.

ii. *Volume changes:* Tumor enhancement volume (signal enhancement ratio) prior to and after therapy will be derived using dedicated software (DynaCAD, Philips) and post-therapy enhancement volume change (%) will be computed. In addition, percentage change in tumor size will be assessed using the following formula for ellipsoidal or spherical volume derived from mid-dynamic sequence subtraction series:
V (cm3) = (4/3 π) × length/2 × width/2 × height/2

iii. *Pharmacokinetic Analysis*: In the subgroup of patients who undergo baseline and follow up MRI imaging in our institution, A 3-parameter, 2-compartment general kinetic model will be used to derive PK parameters (Ktrans, kep, and ve). A Weinmann arterial input function will be used as the population model. Parameters Ktrans (min−1), kep (min−1), and ve (unitless) will be computed from the change in signal intensity data, which is converted to gadolinium concentration pixel-by-pixel within a defined ROI. The mean values of these parameters will be compared pre-and post therapy.

iv. *Kinetic changes:* Model-independent analysis will be performed by directly measuring of the slopes of signal intensity wash-in and wash-out curves (maximum slope, sec-1), contrast enhancement ratio, and IAUC90.

v. *Diffusion Weighted Imaging when performed- changes* within the tumor, both DWI signal and ADC values will be measured and compared to pre-treatment values as applied.

<u>MRI Analysis</u>
A univariate logistic regression model will be used to estimate the odds of response (complete or partial), estimating the odds ratio for each potential imaging parameter with a 95% confidence interval. Logistic regression analysis will be performed to assess the relationship between imaging response parameters (volume, kinetic and DWI) and pathologic (complete or partial) response."

6.1.3 Progression-Free Survival

Progression-free survival (PFS) is defined as the duration of time from start of treatment to time of progression.

6.2 Criteria for Cosmetic Outcome Photography

Photographs of both breasts will be taken and cosmesis will be graded by the patient, and the radiation oncologist, at twelve months from the start of therapy and at yearly intervals thereafter for up to 5 years after treatment. In addition, an independent panel established at University of Texas Southwestern Medical Center will evaluate cosmesis at the end of study based on photography. See Appendix III and IV for physician and patient cosmesis forms.

<u>Excellent</u>: When compared to the untreated breast, there is minimal or no difference in the sizes, shape, or texture of the treated breast. There may be mild thickening or scar tissue within the breast or skin, but not enough to change the appearance.

STU2019-1183, Rahimi, FormA-Protocol, Mod_15, 11-01-23 (7)

Good: There is mild asymmetry in the size or shape of the treated breast as compared to the normal breast. The thickening or scar tissue within the breast causes only mild change in the shape.

Fair: There is obvious difference in the size and shape of the treated breast. This change involves ¼ or less of the breast.

Poor: Marked change in the appearance of the treated breast involving more than ¼ of breast tissue

6.3 Safety/tolerability

Analyses will be performed for all subjects having received at least one fraction of radiation therapy. The study will use the CTCAE version 5.0 for reporting of adverse events.

https://ctep.cancer.gov/protocolDevelopment/electronic_applications/ctc.htm

7.0 ADVERSE EVENTS

7.1 Experimental Therapy

7.1.1 Adverse Reactions

Rib Fracture

The consequences of rib fracture should all be graded according to the Common Terminology Criteria For Adverse Events (CTCAE).

Skin

Monitored treatment related toxicity associated with skin function will include fibrosis, rash (desquamation), blistering, ulceration, and telangiectasia. The consequences of skin toxicity should all be graded according to the Common Terminology Criteria For Adverse Events (CTCAE).

Radiation Pneumonitis

Radiation pneumonitis is unlikely to be seen in patients treated on this protocol. Radiation pneumonitis is a subacute (weeks to months from treatment) inflammation of the end bronchioles and alveoli. Note: It is very important that a Radiation Oncologist participate in the care of the patient, as the clinical picture may be very similar to acute bacterial pneumonia, with fatigue, fever, shortness of breath, nonproductive cough, and a pulmonary infiltrate on chest x-ray.

Patients reporting symptoms as above will be promptly evaluated and treated. Mild radiation pneumonitis may be treated with nonsteroidal anti-inflammatory agents or steroid inhalers. More significant pneumonitis will be treated with systemic steroids, bronchodilators, and pulmonary toilet. Supra- and concurrent infections should be treated with antibiotics. Consideration of prophylaxis of opportunistic infections should be considered in immunocompromised patients.

Radiation pneumonitis will be graded according to its constituent effects including fever, dyspnea, chest pain, and cough according to the Common Terminology Criteria For Adverse Events (CTCAE) version 5.

Neurological (intercostal or brachial plexus nerves) or any grade 4 or 5 toxicity deemed definitely attributed to the therapy.

STU2019-1183, Rahimi, FormA-Protocol, Mod_15, 11-01-23 (7)

Other Toxicities
Other treatment related toxicity attributed to the therapy will be captured, recorded and the consequences of should all be graded according to the Common Terminology Criteria For Adverse Events (CTCAE) version 5.

7.2 Adverse Event Monitoring

Adverse event data collection and reporting, which are required as part of every clinical trial, are done to ensure the safety of subjects enrolled in the studies as well as those who will enroll in future studies. Adverse events are assessed in a routine manner at scheduled times during a trial. Additionally, certain adverse events must be reported in an expedited manner to allow for optimal monitoring of subject safety and care.

All subjects experiencing an adverse event, regardless of its relationship to study therapy, will be monitored until:

- the adverse event resolves or the symptoms or signs that constitute the adverse event return to baseline or is stable in the opinion of the investigator;
- there is a satisfactory explanation other than the study therapy for the changes observed; or
- death.

Note: Adverse events that occur from consent to the end of the acute adverse events reporting period as defined in section 7.2.1 will be documented. Any event that occurs during the late adverse event period as defined in section 7.2.1 and is attributed (possibly, probably, or definitely) to the agent(s) must also be documented and reported if it meets Serious Adverse Event criteria (See Section 7.3).

7.2.1 Definitions

An adverse event is defined as any untoward or unfavorable medical occurrence in a human research study participant, including any abnormal sign (for example, abnormal physical exam, imaging finding or clinically significant laboratory finding), symptom, clinical event, or disease, temporally associated with the subject's participation in the research, whether or not it is considered related to the subject's participation in the research.

Adverse events encompass clinical, physical and psychological harms. Adverse events occur most commonly in the context of biomedical research, although on occasion, they can occur in the context of social and behavioral research. Adverse events may be expected or unexpected.

Acute Adverse Events

Adverse events occurring in the time period from the delivery of radiation, through *90* days post treatment will be considered acute adverse events. *Adverse events will only be captured from medical oncology, radiation oncology, and surgical oncology clinic notes.*

A dose- limiting toxicity (DLT) is a grade 3 toxicity deemed definitely related to treatment in the following categories: skin, rib bone (fracture), pulmonary (radiation pneumonitis), or neurological (intercostal or brachial plexus nerves) or any grade 4 or 5 toxicity deemed definitely attributed to the therapy.

The following acute (<90 days) and late (>90 days – 36 months) toxicities definitely related to protocol treatment, as defined in CTCAE v5.0 will be reported

Grade 4 or higher toxicity in the below categories:
- Rib fracture
- Skin
- Radiation pneumonitis
- Neurologic
- Fat necrosis

Late Adverse Events

Adverse events occurring in the time period from the end of acute monitoring (90 days), to 36 months/3 years post treatment, will be defined as late adverse events. After the 90 day acute period, we will only follow for grade 2 or higher toxicity: skin, rib/bone, bone (fracture), pulmonary (radiation pneumonitis), or neurological (intercostal or brachial plexus nerves) or any grade 4 or 5 toxicity deemed definitely attributed to the therapy. ***Adverse events will only be captured from medical oncology, radiation oncology, and surgical oncology clinic notes.***

A dose- limiting toxicity (DLT) is a grade 3 toxicity deemed definitely related to treatment in the following categories: skin, rib bone (fracture), pulmonary (radiation pneumonitis), or neurological (intercostal or brachial plexus nerves) or any grade 4 or 5 toxicity deemed definitely attributed to the therapy.

Severity

Adverse events will be graded by a numerical score according to the defined NCI Common Terminology Criteria for Adverse Events (NCI CTCAE) Version 5.0. Adverse events not specifically defined in the NCI CTCAE will be scored on the Adverse Event log according to the general guidelines provided by the NCI CTCAE and as outlined below.

- Grade 1: Mild
- Grade 2: Moderate
- Grade 3: Severe or medically significant but not immediately life threatening
- Grade 4: Life threatening consequences
- Grade 5: Death related to the adverse event

Serious Adverse Events

OHRP and UTSW HRPP define serious adverse events as those events, occurring at any dose, which meets any of the following criteria:
- Results in death
- is life-threatening (places the subject at immediate risk of death from the event as it occurred);
- Results in inpatient hospitalization[1,2] or prolongation of existing hospitalization;
- Results in persistent or significant disability/incapacity;
- Results in a congenital anomaly/birth defect; or
- Based upon appropriate medical judgment, may jeopardize the subject's health and may require medical or surgical intervention to prevent one of the other outcomes listed in this definition.

Note: A "Serious adverse event" is by definition an event that meets ***any*** of the above criteria. Serious adverse events may or may not be related to the research project. A

serious adverse event determination does not require the event to be related to the research. That is, both events completely unrelated to the condition under study and events that are expected in the context of the condition under study may be serious adverse events, independent of relatedness to the study itself. As examples, a car accident requiring >24 hour inpatient admission to the hospital would be a serious adverse event for any research participant; likewise, in a study investigating end-stage cancer care, any hospitalization or death which occurs during the protocol-specified period of monitoring for adverse and serious adverse events would be a serious adverse event, even if the event observed is a primary clinical endpoint of the study.

[1]Pre-planned hospitalizations or elective surgeries are not considered SAEs. Note: If events occur during a pre-planned hospitalization or surgery, that prolong the existing hospitalization, those events should be evaluated and potentially reported as SAEs.

[2] NCI defines hospitalization for expedited AE reporting purposes as an inpatient hospital stay equal to or greater than 24 hours. Hospitalization is used as an indicator of the seriousness of the adverse event and should only be used for situations where the AE truly fits this definition and NOT for hospitalizations associated with less serious events. For example: a hospital visit where a patient is admitted for observation or minor treatment (e.g. hydration) and released in less than 24 hours. Furthermore, hospitalization for pharmacokinetic sampling is not an AE and therefore is not to be reported either as a routine AE or in an expedited report.

7.2.2 Unanticipated Problems Involving Risks to Subjects or Others (UPIRSOs):

The phrase “unanticipated problems involving risks to subjects or others” is found, but not defined in the HHS regulations at 45 CFR 46, and the FDA regulations at 21 CFR 56.108(b)(1) and 21 CFR 312.66. Guidance from the regulatory agencies considers unanticipated problems to include any incident, experience, or outcome that meets ALL three (3) of the following criteria:

- Unexpected in terms of nature, severity or frequency given (a) the research procedures that are described in the protocol-related documents, such as the IRB-approved research protocol and informed consent document; and (b) the characteristics of the subject population being studied;

AND

- Related or possibly related to participation in the research (possibly related means there is a reasonable possibility that the incident, experience, or outcome may have been caused by the procedures involved in the research);

AND

- Suggests that the research places subjects or others at greater risk of harm (including physical, psychological, economic, or social harm) than was previously known or recognized. Note: According to OHRP, if the adverse event is serious, it would always suggest a greater risk of harm.

Follow-up

All adverse events will be followed up according to good medical practices.

7.3 Steps to Characterize a Serious Adverse Event for Reporting to the SCCC DSMC

Step 1: Identify the type of adverse event using the NCI Common Terminology Criteria for Adverse Events (CTCAE v5.0).

Step 2: Grade the adverse event using the NCI CTCAE v5.0

Step 3: Determine whether the adverse event is related to the protocol therapy. Attribution categories are as follows:
- Definite – The AE *is clearly related* to the study treatment.
- Probable – The AE *is likely related* to the study treatment.
- Possible – The AE *may be related* to the study treatment.
- Unlikely – The AE *may NOT be related* to the study treatment.
- Unrelated – The AE *is clearly NOT related* to the study treatment.

Note: This includes all events that occur within 30 days of the last dose of protocol treatment to the end of the acute adverse events reporting period as defined in section 7.2.1). Any event that occurs during the late adverse event period as defined in section 7.2.1) and is attributed (possibly, probably, or definitely) to the agent(s) must also be reported as indicated in the sections below.

Step 4: Determine the expectedness of the adverse event. Expected events are those that have been previously identified as resulting from administration of the treatment. An adverse event is considered unexpected, for expedited reporting purposes only, when either the type of event or the severity of the event is not listed in:

- the current known adverse events listed in the Agent Information Section of this protocol (if applicable);
- the drug package insert (if applicable);
- the current Investigator's Brochure (if applicable)
- the Study Agent(s)/Therapy(ies) Background and Associated Known Toxicities section of this protocol

7.3.1 Reporting SAEs and UPIRSOs to the Simmons Comprehensive Cancer Center (SCCC) Data Safety Monitoring Committee (DSMC)

SAEs and UPIRSOs at all sites, which occur in research subjects on protocols for which the SCCC is the DSMC of record require reporting to the DSMC regardless of whether IRB reporting is required. All SAEs occurring during the protocol-specified monitoring period and all UPIRSOs should be submitted to the SCCC DSMC within 5 business days of the study team members awareness of the event(s). In addition, for participating centers other than UTSW, local IRB guidance should be followed for local reporting of serious adverse events or unanticipated problems.

The UTSW study PI is responsible for ensuring SAEs/UPIRSOs are submitted to the SCCC DSMC Coordinator. This may be facilitated by the IIT project manager, study team, sub-site or other designee. Electronic versions of the eIRB Reportable Event report; FDA Form #3500A forms, or other sponsor forms, if applicable; and/or any other supporting documentation available should be submitted to the DSMC.

UT Southwestern and affiliates will submit documentation via the SAE submission portal (InfoReady). The DSMC Coordinator will route the form to the DSMC Chair who determines if immediate action is required. Follow-up eIRB reports, and all subsequent SAE or UPIRSO documentation that is available are also submitted to the DSMC Chair who determines if further action is required via the same process. *(See Appendix V of the SCCC DSMC Plan for instructions on how to submit SAEs through the portal.*

If the event occurs on a multi-institutional clinical trial coordinated by the UTSW Simmons Comprehensive Cancer Center, the IIT Project Manager or designee ensures that all participating sites are notified of the event and resulting action, according to FDA guidance for expedited reporting. DSMC Chair reviews all SAEs and UPIRSOs upon receipt from the DSMC Coordinator. The DSMC Chair determines whether action is required and either takes action immediately, convenes a special DSMC session

(physical or electronic), or defers the action until a regularly scheduled DSMC meeting.

Telephone reports to:<br>(Investigator/study team: Insert names and phone numbers for required notifications)<br><br>UTSW Radiation Oncology Clinical Research Manager or Study Coordinator<br>Email: Sarah.Hardee@UTSouthwestern.edu<br>Phone: 214-648-1836<br>Fax: 214-645-0780
Written reports to:<br>Written reports to: UTSW Radiation Oncology Clinical Research Manager or Study Coordinator<br>Email: Sarah.Hardee@UTSouthwestern.edu<br>Fax: 214-645-0780<br><br>UTSW SCCC Data Safety Monitoring Committee<br>Website for entering SAEs:<br>Email: https://utsouthwestern.infoready4.com/<br><br>UTSW Institutional Review Board (IRB)<br>Submit a Reportable Event via eIRB with a copy of the final sponsor report as attached supporting documentation

Reporting Unanticipated Problems Involving Risks to Subjects or Others (UPIRSOs) to the UTSW HRPP

UTSW reportable event guidance applies to all research conducted by or on behalf of UT Southwestern, its affiliates, and investigators, sites, or institutions relying on the UT Southwestern IRB. Additional reporting requirements apply for research relying on a non-UT Southwestern IRB.

According to UTSW HRPP policy, UPIRSOs are incidents, experiences, outcomes, etc. that meet **ALL three (3)** of the following criteria:

1. Unexpected in nature, frequency, or severity (i.e., generally not expected in a subject's underlying condition or not expected as a risk of the study; therefore, not included in the investigator's brochure, protocol, or informed consent document),AND
2. Probably or definitely related to participation in the research, AND
3. Suggests that the research places subjects or others at a greater risk of harm (including physical, psychological, economic, or social harm) than was previously known or recognized. Note: According to OHRP, if the adverse event is serious, it would always suggest a greater risk of harm.

UPIRSOs must be promptly reported to the UTSW HRPP within 5 working days of PI awareness.

Events NOT meeting UPIRSO criteria:

Events that do NOT meet UPIRSO criteria should be tracked, evaluated, summarized, and submitted to the UTSW HRPP/IRB at continuing review.

For more information on UTSW HRPP/IRB reportable event policy, see

STU2019-1183, Rahimi, FormA-Protocol, Mod_15, 11-01-23 (7)

policy_9.5reportable.pdf (utsouthwestern.edu)

7.4 Stopping Rules

See statistical section 10.1 Phase I dose escalation and waiting periods.

8.0 MISCELLANEOUS INFORMATION

8.1 Lumpectomy Guidelines

These guidelines are based on NSABP experience and implementation in previous trials with experience resulting in improved cosmesis. These guidelines are suggestions and are not mandatory for patient entry or protocol compliance.

Lumpectomy should be performed 8 to 52 weeks after XRT in a manner that achieves good cosmesis with adequate resection of the tumor with negative margins. Examples of practices leading to good cosmesis are: use of curvilinear incisions for lesions located in the upper half of the breast, use of radial incisions in the lower half of the breast, avoidance of drains in the lumpectomy cavity, separate incisions for the lumpectomy and axillary dissection.

The initial biopsy should be performed as if it was a lumpectomy; i.e., precautions should be taken to ensure that the margins of the resected tissue are grossly free of tumor thus avoiding a re-excision of breast tissue if biopsy is positive for cancer and the final margins are histologically negative. Margins are considered negative if there is no invasive or non-invasive tumor within 2 mm of the inked margin.

Hormonal Therapy

Endocrine therapy after pre-operative radiation should be started within 21 days after pre-operative radiation, but it is encouraged to start the day after post-radiation biopsy.

9.0 CORRELATIVES/SPECIAL STUDIES

Submission of samples for correlative studies is optional.

The goal of the planned laboratory correlative studies is to:

Exploratory endpoints:

- Primary tumor biopsies (pending volume of tissue)
  - Expression of PD-L1, PD-L2, PD1, CD3, CD68, CD8, Ki67.
  - RNAseq analyses including RNA profiling.
  - Whole exome sequencing to determine mutational burden and neoantigen detection.
  - T-cell receptor sequencing to determine intratumoral TCR diversity.
  - IHC to quantify immune populations.
- Blood (both Serum and PBMC)
  - T-cell receptor sequencing to determine tumor-specific TCR diversity, expansion and contraction.
  - Analysis of antibody response (both quantitative antibody titer and qualitative antibody binding and functionality).
  - Study of T-cell composition and functioning.
  - Identification of circulating antigen, DNA or tumor cells.
  - Analysis of cytokine response.
  - Analysis of blood cell composition using flow cytometry or mass cytometry.
  - Analysis of inflammatory status

STU2019-1183, Rahimi, FormA-Protocol, Mod_15, 11-01-23 (7)

9.1 Optional Sample Collection Guidelines

Samples will be labeled with the subject's de-identified study number and collection date.

Whole blood and serum sample: Patient's whole blood will be collected in four 10 mL Lavender Top EDTA tubes at baseline within 30 days prior to radiotherapy, 4 weeks after radiotherapy, and 4 weeks after surgery. In addition, 10 mL will be collected in anti-coagulant-free tubes (Red top) for the collection of serum. Approximately 50 mL of blood will be collected at each visit. The blood will immediately be processed (within 2 hours) by centrifugation (1000g, 15min, 4°C), collecting the supernatant and freezing at -80°C in 5 aliquots for future experiments. The pellet will be re-suspended in PBS and PBMC will be isolated using standard protocol. Briefly, the cell suspension will be carefully placed on 10mL polystyrene tube containing 1ml ficoll and centrifuged (400g, 30min, RT). Collect the PBMC region from the ficoll and washed 3x with PBS. Count and freeze cells in 5 aliquots with 10%DMSO 90%FBS in -80oC.

Optional Blood samples at each time point of 30 days prior to XRT, 4 weeks after XRT, and 4 weeks after surgery (see Section 5.4 for sample collection windows):

- 4 10 mL Lavender top tubes (whole blood)
- 1 10 mL Red top tube (serum)

Tumor Sample: An image-guided biopsy of tumor lesion consisting of 4-5 18G needle cores is recommended at the time of tissue diagnosis. If the patient participated in protocols such as the Pathology Tissue Repository Protocol or procedures and the tissue confirming breast cancer diagnosis is in storage and available at UTSW Medical Center or an outside institution, the study team may request a tissue sample. The study team will also request frozen and formalin-fixed paraffin embedded (FFPE) samples from surgical specimen. These samples may be used for generation of tumor lysates (which can be used as source of antigens for immunoassays). The samples may also be used for a variety of assays, including but not limited to ELISA, Elispot assay, ^{3}H-thymidine Proliferation Assay, Chromium Release Cytotoxicity Assays, Flow cytometric analysis (FACS), Immunohistochemical staining (IHC), Serum Cytokine Analysis and Western-blot/Immuno-blot analysis. DNA and RNA sequencing may be performed and de-identified sequence may be submitted to publicly accessible databases as required by journal or institutional requirements

9.2 Specimen Banking

Subject samples collected for this study will be retained at the UTSW Radiation Oncology biorepository. Specimens will be stored indefinitely or until they are used up. If future use is denied or withdrawn by the subject, best efforts will be made to stop any additional studies and to destroy the specimens.

Prasanna Alluri MD/PhD or Raquibal Hannan, MD, PhD will be responsible for reviewing and approving requests for clinical specimen from potential research collaborators outside of UTSW. Collaborators will be required to complete an agreement (a Material Transfer Agreement or recharge agreement) that states specimens will only be released for use in disclosed research. Any data obtained from the use of clinical specimen will be the property of UTSW for publication and any licensing agreement will be strictly adhered to.

The specimens, DNA, and their derivatives may have significant therapeutic or commercial value. The Informed Consent form contains this information and informs the subject that there is the potential for financial gain by UTSW the investigator or a collaborating researcher or entity.

STU2019-1183, Rahimi, FormA-Protocol, Mod_15, 11-01-23 (7)

The following information obtained from the subject's medical record may be provided to research collaborators when specimens are made available:

- Diagnosis
- Collection time in relation to study treatment
- Clinical outcome – if available
- Demographic data

10.0 STATISTICAL CONSIDERATIONS

10.1 Study Design/Study Endpoints

Primary Endpoint

The primary endpoint of the phase I portion is to either reach the maximum tolerated dose (MTD) or a dose of 38 Gy total (whichever comes first) by escalating the dose of SBRT toward the tumorcidal dose of 38 Gy in fraction. Patients will be treated in cohorts of seven to fifteen. Toxicity will be graded using the NCI Common Toxicity Criteria for Adverse Events (CTCAE) v. 5.0. ***A dose- limiting toxicity (DLT) is a grade 3 toxicity deemed definitely related to treatment in the following categories: skin, rib bone (fracture), pulmonary (radiation pneumonitis), or neurological (intercostal or brachial plexus nerves) or any grade 4 or 5 toxicity deemed definitely attributed to the therapy.*** All reported DLTs will be verified by study chair, data and safety monitoring committee, and, as appropriate, independent review before final determination that a DLT has in fact occurred. Doses will be escalated an additional 3.5-4 Gy per treatment. The phase I portion of the study will be completed when either of the following events occur: 1) the MTD for a cohort is reached or 2) when delivery of a pre-determined highest dose of radiation (38 Gy) that has been deemed likely to be efficacious for treatment of early stage breast cancer is attained.

Secondary Endpoints

Secondary endpoints for the phase I portion of this study include cosmetic outcome as evaluated by patient and physician. In addition, the following endpoints will be evaluated as secondary endpoints:

- Local Control
- Acute Toxicity (90 Days)
- Late Toxicity (24 months)
- Rates of Surgical Morbidity
- Pathologic Complete Response Rates
- Patient and Physician Cosmesis Outcomes
- Distant Disease-Free Survival

A "Therapeutic" Phase I Trial

Most (not all) oncology-related drug discovery phase I trials are non-therapeutic. In fact, the drug doses being studied has often never been used in humans for the stated indication. Therefore, the dose used may be wholly ineffective at providing efficacy to the patient. Second, the typical drug discovery phase I trial focuses primarily on toxicity dose response, with no formal plan to evaluate efficacy. In this context, it is assumed that the optimal dose will be the maximum tolerated dose. Neither assumption is true for the radiotherapy used in this phase I trial. Since radiotherapy is well known to provide efficacy,

even the starting dose of this trial, this phase I trial is a therapeutic trial. Furthermore, the maximum tolerated dose may or may not be the most ideal dose balancing benefit and toxicity with the widest therapeutic window. While the primary endpoint and statistical design focuses on toxicity, efficacy information regarding tumor response/control will be collected (see below).

Phase I Dose Escalation
The phase I study is designed to end if the rate of DLTs within 90 days from the start of treatment is 33% or higher. For each dose level cohorts, a total of seven to fifteen patients will be enrolled. If zero out of the first seven with 90 day follow-up, two or fewer out of the first nine with 90 day follow-up, three or fewer out of the first twelve with 90 day follow-up, or four or fewer out of the first fifteen of patients with 90 day follow-up experience a DLT as defined above, then the dose will escalate to the next dose level. If three or more of the first nine patients, four or more of the first twelve, or five or more of the first fifteen patients experience a DLT, then the MTD will be considered to have been exceeded. The MTD will be defined as the immediately previous lower dose level tolerated.

Phase I Waiting Periods
Dose escalation on the phase I portion of this study should not occur until a sufficient waiting period has occurred after patients have been treated. A period of 90 days must pass in order to assess toxicity. If 90 days have transpired without DLT in each of the first seven (7) patients enrolled to a specific dose level, then dose escalation to the next level may proceed. Patients will continue to be enrolled to each dose level (up to a maximum of 15 patients) with ongoing assessment of those reaching 90 day follow-up so long as either criteria for defining the MTD or criteria for further dose escalation is not reached. If fifteen patients are enrolled to a given dose level yet criteria for adequate follow-up are not reached in a representative sample of patients, further enrollment to the protocol will be suspended until adequate follow-up is reached.

10.2 Sample Size and Accrual

While accrual realities may limit total enrollment to only 7 patients per dose level, the investigators will attempt to enroll up to 15 patients per dose level in an effort to both improve the toxicity determination as well as afford some evaluation of efficacy. The sample size of the phase I component of this study will not exceed 60 patients.

10.3 Data Analyses

Data Analysis Plans

Interim Reports: Interim reports will be prepared every twelve months from time of enrollment of first patient until enrollment is complete. In general, the interim reports will contain information about patient accrual rate with projected completion dates of the trial, status of QA review and compliance rate of treatment per protocol, and the frequencies and severity of toxicity.

The dose-limiting toxicity (DLT) and the maximum tolerable dosage (MTD) will be determined based on this Phase I design. The assessment of safety will be mainly based on the frequency of adverse events. The incidence of AEs together with the 95% confidence interval will be reported. The overall complete response rate and corresponding 95% confidence interval will be presented. Various survival rates such as local control, recurrence free survival, and distant disease-free interval will be computed using Kaplan-Meier curves along with the 95% confidence interval. Exact binomial method will be used to calculate the response rate, toxicity and the corresponding 95% confidence interval.

- Kaplan-Meier method will used to estimate the distant disease-free interval.

11.0 STUDY MANAGEMENT

11.1 Conflict of Interest

Any investigator who has a conflict of interest with this study (patent ownership, royalties, or financial gain greater than the minimum allowable by their institution, etc.) must have the conflict reviewed by the UTSW COI Committee and IRB according to UTSW Policy on Conflicts of Interest. All investigators will follow the University conflict of interest policy.

11.2 Institutional Review Board (IRB) Approval and Consent

It is expected that the IRB of record will have the proper representation and function in accordance with federally mandated regulations. The IRB of record must approve the consent form and protocol.

In obtaining and documenting informed consent, the investigator should comply with the applicable regulatory requirement(s), and should adhere to Good Clinical Practice (GCP) and to ethical principles that have their origin in the Declaration of Helsinki.

Before recruitment and enrollment onto this study, the subject will be given a full explanation of the study and will be given the opportunity to review the consent form. Each consent form must include all the relevant elements currently required by the FDA Regulations and local or state regulations. Once this essential information has been provided to the subject and the investigator is assured that the subject understands the implications of participating in the study, the subject will be asked to give consent to participate in the study by signing an IRB-approved consent form.

Prior to a patient's participation in the trial, the written informed consent form should be signed and personally dated by the subject and by the person who conducted the informed consent discussion.

11.3 Registration/Randomization Procedures

All subjects must be registered with the Radiation Oncology CRO before enrollment to study. Prior to registration, eligibility criteria must be confirmed with the Radiation Oncology CRO Study Coordinator.

New subjects will receive a number beginning with 001 upon study consent such that the first subject consented is numbered 001, the second subject consented receives the number 002, etc.

Upon confirmation of eligibility and enrollment as per the afore-mentioned instructions, the subject will be assigned a secondary number in the order of enrollment. For example, subject 001 will become 001-01 upon enrollment. If subject 002 screen fails, and subject 003 is the next subject enrolled, subject 003 will become 003-02 and so-on.

Each newly consented subject should be numbered using the schema provided above. Upon registration, the registrar will assign the additional registration/randomization code according to the numbering schema outlined above, which should then be entered as the patient study id in Velos upon updating the status to enrolled.

The numbering schema should clearly identify the site number; the sequential number of the subject consented as well as the status of the subjects enrolled so that the number of subjects consented versus the number of subjects actually enrolled may be easily identified.

11.4 Data Management and Monitoring/Auditing

11.4.1 Data Management

REDCap is the UTSW SCCC institutional choice for the electronic data capture of case report forms for SCCC Investigator Initiated Trials. REDCap will be used for electronic case report forms in accordance with Simmons Comprehensive Cancer Center requirements, as appropriate for the project.

11.4.2 Trial Monitoring

Trial monitoring will be conducted no less than annually and refers to a regular interval review of trial related activity and documentation performed by the DOT, which includes but is not limited to accuracy of case report forms, protocol compliance, timeless and accuracy of Velos entries and AE/SAE management and reporting. Documentation of trial monitoring will be maintained along with other protocol related documents and will be reviewed during internal audit.

11.4.3 Trial Audits

Toxicity reviews will be performed annually from the day of the first patient enrollment, and dose escalation and/or de-escalation will be performed at the time of dose escalation and/or de-escalation on this study. The report will be stored in the digital regulatory repository.

The UTSW Simmons Comprehensive Cancer Center (SCCC) Data Safety Monitoring Committee (DSMC) is responsible for monitoring data quality and patient safety for all UTSW SCCC clinical trials. As part of that responsibility, the SCCC DSMC reviews all serious adverse events and UPIRSOs in real time as they are reported and reviews adverse events on a quarterly basis. The quality assurance activity for the Clinical Research Office provides for periodic auditing of clinical research documents to ensure data integrity and regulatory compliance. A copy of the DSMC plan is available upon request.

The SCCC DSMC meets quarterly and conducts annual comprehensive reviews of ongoing clinical trials, for which it serves as the DSMC of record. The Quality Assurance Coordinator (QAC) works as part of the DSMC to conduct regular audits based on the level of risk. Audit findings are reviewed at the next available DSMC meeting. In this way, frequency of DSMC monitoring is dependent upon the level of risk. Risk level is determined by the DSMC Chair and a number of factors such as the phase of the study; the type of investigational agent, device or intervention being studied; and monitoring required to ensure the safety of study subjects based on the associated risks of the study. Protocol-specific DSMC plans must be consistent with these principles.

11.5 Adherence to the Protocol

Except for an emergency situation, in which proper care for the protection, safety, and well-being of the study subject requires alternative treatment, the study shall be conducted exactly as described in the approved protocol.

Any deviation from the protocol requirements, whether pre-approved or unexpected, are to be recorded/logged as a protocol deviation and reported per institutional policy.

STU2019-1183, Rahimi, FormA-Protocol, Mod_15, 11-01-23 (7)

11.5.1 Exceptions (also called single-subject exceptions or single-subject waivers): include any departure from IRB-approved research that is *not due to an emergency* and is:

- intentional on part of the investigator; and/or
- in the investigator's control; and/or
- not intended as a systemic change (e.g., single-subject exceptions to eligibility [inclusion/exclusion] criteria)

➢ **Reporting requirement***: Exceptions are non-emergency deviations that require ***prospective*** IRB approval before being implemented. Call the IRB if your request is urgent. If IRB approval is not obtained beforehand, this constitutes a major deviation. For eligibility waivers, studies which utilize the SCCC-DSMC as the DSMC of record must also obtain approval from the DSMC prior to submitting to IRB for approval.

11.5.2 Emergency Deviations: include any departure from IRB-approved research that is necessary to:

- avoid immediate apparent harm, and/or
- protect the life or physical well-being of subjects or others

➢ **Reporting requirement***: Emergency deviations must be promptly reported to the IRB within 5 working days of occurrence.

11.5.3 **Serious Noncompliance (formerly called major deviations or violations): include any departure from IRB-approved research that**:

- increase risk of harm to subjects; and/or
  adversely affects the rights, safety, or welfare of subjects (any of which may also be an unanticipated problem); and/or
- adversely affects the integrity of the data and research (i.e., substantially compromises the integrity, reliability, or validity of the research)

➢ **Reporting requirement***: Serious noncompliance must be promptly reported to the IRB within 5 working days of discovery.

11.5.4 **Continuing Noncompliance:** includes a pattern of repeated noncompliance (in one or more protocols simultaneously, or over a period of time) which continues **after** initial discovery, including inadequate efforts to take or implement corrective or preventive action within a reasonable time frame.

➢ **Reporting requirement*:** Continuing Noncompliance must be promptly reported to the IRB within 5 working days of discovery.

11.5.5 **Noncompliance (that is neither serious nor continuing; formerly called minor deviations) any departure from IRB-approved research that:**

- Does not meet the definition of serious noncompliance or continuing noncompliance

  ➢ **Reporting requirement***: Noncompliance that is neither serious nor continuing should be tracked and summarized at the next IRB continuing review, or the notice of study closure- whichever comes first.

*Reporting Requirements reflect UTSW HRPP/IRB guidelines; participating sites should follow the reporting guidelines for their IRB of record.

11.6 Amendments to the Protocol

Should amendments to the protocol be required, the amendments will be originated and documented by the Principal Investigator. A summary of changes document outlining proposed changes as well as rationale for changes, when appropriate, is highly recommended. When an amendment to the protocol substantially alters the study design or the potential risk to the patient, a revised consent form might be required.

The written amendment, and if required the amended consent form, must be sent to the IRB for approval prior to implementation.

11.7 Record Retention

Study documentation includes all Case Report Forms, data correction forms or queries, source documents, Sponsor-Investigator correspondence, monitoring logs/letters, and regulatory and essential documents (e.g., protocol and amendments, IRB correspondence and approval, signed patient consent forms).

Source documents include all recordings of observations or notations of clinical activities and all reports and records necessary for the evaluation and reconstruction of the clinical research study.

Government agency regulations and directives require that the study investigator retain all study documentation pertaining to the conduct of a clinical trial. In the case of a study with a drug seeking regulatory approval and marketing, these documents shall be retained for at least two years after the last approval of marketing application in an International Conference on Harmonization (ICH) region. In all other cases, study documents should be kept on file until three years after the completion and final study report of this investigational study.

11.8 Obligations of Investigators

The Principal Investigator is responsible for the conduct of the clinical trial at the site in accordance with Title 21 of the Code of Federal Regulations and/or the Declaration of Helsinki. The Principal Investigator is responsible for personally overseeing the treatment of all study patients. The Principal Investigator must assure that all study site personnel, including sub-investigators and other study staff members, adhere to the study protocol and all FDA/GCP/NCI regulations and guidelines regarding clinical trials both during and after study completion.

The Principal Investigator at each institution or site will be responsible for assuring that all the required data will be collected and entered onto the Case Report Forms. Periodically, monitoring visits may be conducted and the Principal Investigator will provide access to his/her original records to permit verification of proper entry of data. At the completion of the study, all case report forms will be reviewed by the Principal Investigator and will require his/her final signature to verify the accuracy of the data.

STU2019-1183, Rahimi, FormA-Protocol, Mod_15, 11-01-23 (7)

12 REFERENCES

1. Fisher B, Anderson S, Bryant J, Margolese RG, Deutsch M, Fisher ER, Jeong JH, Wolmark N. Twenty-year follow-up of a randomized trial comparing total mastectomy, lumpectomy, and lumpectomy plus irradiation for the treatment of invasive breast cancer. N Engl J Med. 2002 Oct 17;347(16):1233-41. PubMed PMID: 12393820
2. van Dongen JA, Voogd AC, Fentiman IS, Legrand C, Sylvester RJ, Tong D, van der Schueren E, Helle PA, van Zijl K, Bartelink H. Long-term results of a randomized trial comparing breast-conserving therapy with mastectomy: European Organization for Research and Treatment of Cancer 10801 trial. J Natl Cancer Inst. 2000 Jul 19;92(14):1143-50. PubMed PMID: 10904087.
3. Veronesi U, Cascinelli N, Mariani L, Greco M, Saccozzi R, Luini A, Aguilar M, Marubini E. Twenty-year follow-up of a randomized study comparing breast-conserving surgery with radical mastectomy for early breast cancer. N Engl J Med. 2002 Oct 17;347(16):1227-32. PubMed PMID: 12393819.
4. Yeboa DN, Xu X, Jones BA, Soulos P, Gross C, Yu JB. Trend in Age and Racial Disparities in the Receipt of Postlumpectomy Radiation Therapy for Stage I Breast Cancer: 2004-2009. Am J Clin Oncol. 2016 Dec;39(6):568-574. PubMed PMID: 24879475.
5. Hughes KS, Schnaper LA, Bellon JR, Cirrincione CT, Berry DA, McCormick B, Muss HB, Smith BL, Hudis CA, Winer EP, Wood WC. Lumpectomy plus tamoxifen with or without irradiation in women age 70 years or older with early breast cancer: long-term follow-up of CALGB 9343. J Clin Oncol. 2013 Jul 1;31(19):2382-7. doi: 10.1200/JCO.2012.45.2615. Epub 2013 May 20. PubMed PMID: 23690420; PubMed Central PMCID: PMC3691356.
6. Kunkler IH, Williams LJ, Jack WJ, Cameron DA, Dixon JM; PRIME II investigators. Breast-conserving surgery with or without irradiation in women aged 65 years or older with early breast cancer (PRIME II): a randomized controlled trial. Lancet Oncol. 2015 Mar;16(3):266-73. doi: 10.1016/S1470-2045(14)71221-5. Epub 2015 Jan 28. Erratum in: Lancet Oncol. 2015 Mar;16(3):e105. PubMed PMID: 25637340.
7. Early Breast Cancer Trialists' Collaborative Group (EBCTCG), Darby S, McGale P, Correa C, Taylor C, Arriagada R, Clarke M, Cutter D, Davies C, Ewertz M, Godwin J, Gray R, Pierce L, Whelan T, Wang Y, Peto R. Effect of radiotherapy after breast-conserving surgery on 10-year recurrence and 15-year breast cancer death: meta-analysis of individual patient data for 10,801 women in 17 randomised trials. Lancet. 2011 Nov 12;378(9804):1707-16. doi: 10.1016/S0140-6736(11)61629-2. Epub 2011 Oct 19. Review. PubMed PMID: 22019144; PubMed Central PMCID: PMC3254252.
8. START Trialists' Group, Bentzen SM, Agrawal RK, Aird EG, Barrett JM, Barrett-Lee PJ, Bliss JM, Brown J, Dewar JA, Dobbs HJ, Haviland JS, Hoskin PJ, Hopwood P, Lawton PA, Magee BJ, Mills J, Morgan DA, Owen JR, Simmons S, Sumo G, Sydenham MA, Venables K, Yarnold JR. The UK Standardisation of Breast Radiotherapy (START) Trial A of radiotherapy hypofractionation for treatment of early breast cancer: a randomised trial. Lancet Oncol. 2008a Apr;9(4):331-41. doi: 10.1016/S1470-2045(08)70077-9. Epub 2008 Mar 19. PubMed PMID: 18356109; PubMed Central PMCID: PMC2323709.
9. START Trialists' Group, Bentzen SM, Agrawal RK, Aird EG, Barrett JM, Barrett-Lee PJ, Bentzen SM, Bliss JM, Brown J, Dewar JA, Dobbs HJ, Haviland JS, Hoskin PJ, Hopwood P, Lawton PA, Magee BJ, Mills J, Morgan DA, Owen JR, Simmons S, Sumo G, Sydenham MA, Venables K, Yarnold JR. The UK Standardisation of Breast Radiotherapy (START) Trial B of radiotherapy hypofractionation for treatment of early breast cancer: a randomised trial. Lancet. 2008b Mar 29;371(9618):1098-107. doi: 10.1016/S0140-6736(08)60348-7. Epub 2008 Mar 19. PubMed PMID: 18355913; PubMed Central PMCID: PMC2277488.
10. Whelan TJ, Pignol JP, Levine MN, Julian JA, MacKenzie R, Parpia S, Shelley W, Grimard L, Bowen J, Lukka H, Perera F, Fyles A, Schneider K, Gulavita S, Freeman C. Long-term results of hypofractionated radiation therapy for breast cancer. N Engl J Med. 2010 Feb 11;362(6):513-20. doi: 10.1056/NEJMoa0906260. PubMed PMID: 20147717.
11. Smith BD, Bellon JR, Blitzblau R, Freedman G, Haffty B, Hahn C, Halberg F, Hoffman K, Horst K, Moran J, Patton C, Perlmutter J, Warren L, Whelan T, Wright JL, Jagsi R. Radiation therapy for the whole breast: Executive summary of an American Society for Radiation Oncology (ASTRO) evidence-based guideline. Pract Radiat Oncol. 2018 May - Jun;8(3):145-152. doi: 10.1016/j.prro.2018.01.012. Epub 2018 Mar 12. PubMed PMID: 29545124.

STU2019-1183, Rahimi, FormA-Protocol, Mod_15, 11-01-23 (7)

12. Fisher ER, Anderson S, Redmond C, Fisher B. Ipsilateral breast tumor recurrence and survival following lumpectomy and irradiation: pathological findings from NSABP protocol B-06. Semin Surg Oncol. 1992 May-Jun;8(3):161-6. PubMed PMID: 1496227.
13. Vicini FA, Kestin LL, Goldstein NS. Defining the clinical target volume for patients with early-stage breast cancer treated with lumpectomy and accelerated partial breast irradiation: a pathologic analysis. Int J Radiat Oncol Biol Phys. 2004 Nov 1;60(3):722-30. PubMed PMID: 15465188.
14. Strnad V, Ott OJ, Hildebrandt G, Kauer-Dorner D, Knauerhase H, Major T, Lyczek J, Guinot JL, Dunst J, Gutierrez Miguelez C, Slampa P, Allgäuer M, Lössl K, Polat B, Kovács G, Fischedick AR, Wendt TG, Fietkau R, Hindemith M, Resch A, Kulik A, Arribas L, Niehoff P, Guedea F, Schlamann A, Pötter R, Gall C, Malzer M, Uter W, Polgár C; Groupe Européen de Curiethérapie of European Society for Radiotherapy and Oncology (GEC-ESTRO). 5-year results of accelerated partial breast irradiation using sole interstitial multicatheter brachytherapy versus whole-breast irradiation with boost after breast-conserving surgery for low-risk invasive and in-situ carcinoma of the female breast: a randomised, phase 3, non-inferiority trial. Lancet. 2016 Jan 16;387(10015):229-38. doi: 10.1016/S0140-6736(15)00471-7. Epub 2015 Oct 19. PubMed PMID: 26494415.
15. Polgár C, Fodor J, Major T, Németh G, Lövey K, Orosz Z, Sulyok Z, Takácsi-Nagy Z, Kásler M. Breast-conserving treatment with partial or whole breast irradiation for low-risk invasive breast carcinoma--5-year results of a randomized trial. Int J Radiat Oncol Biol Phys. 2007 Nov 1;69(3):694-702. Epub 2007 May 25. PubMed PMID: 17531400.
16. Yashar C, Attai D, Butler E, Einck J, Finkelstein S, Han B, Hong R, Komarnicky L, Lyden M, Mantz C, Morcovescu S, Nigh S, Perry K, Pollock J, Reiff J, Scanderbeg D, Snyder M, Kuske R. Strut-based accelerated partial breast irradiation: Report of treatment results for 250 consecutive patients at 5 years from a multicenter retrospective study. Brachytherapy. 2016 Nov - Dec;15(6):780-787. doi: 10.1016/j.brachy.2016.07.002. Epub 2016 Aug 12. PubMed PMID: 27528591.
17. Vargo JA, Verma V, Kim H, Kalash R, Heron DE, Johnson R, Beriwal S. Extended (5-year) outcomes of accelerated partial breast irradiation using MammoSite balloon brachytherapy: patterns of failure, patient selection, and dosimetric correlates for late toxicity. Int J Radiat Oncol Biol Phys. 2014 Feb 1;88(2):285-91. doi: 10.1016/j.ijrobp.2013.05.039. Epub 2013 Nov 21. PubMed PMID: 24268787.
18. Olivotto IA, Whelan TJ, Parpia S, Kim DH, Berrang T, Truong PT, Kong I, Cochrane B, Nichol A, Roy I, Germain I, Akra M, Reed M, Fyles A, Trotter T, Perera F, Beckham W, Levine MN, Julian JA. Interim cosmetic and toxicity results from RAPID: a randomized trial of accelerated partial breast irradiation using three-dimensional conformal external beam radiation therapy. J Clin Oncol. 2013 Nov 10;31(32):4038-45. doi: 10.1200/JCO.2013.50.5511. Epub 2013 Jul 8. PubMed PMID: 23835717.
19. Whelan T, Julian J, Levine M, Berrang T, Kim D-H, Gu CS, Germain I, Nichol A, Akra M, Lavertu S, Germain F, Fyles A, Trotter T, Perera F, Balkwill S, Chafe S, McGowan T, Muanza T, Beckham W, Chua B, Olivotto I. RAPID: A randomized trial of accelerated partial breast irradiation using 3-dimensional conformal radiotherapy (3D-CRT). Oral presentation at SABCS Symposium 2018. Abstract GS4-03.
20. Coles CE, Griffin CL, Kirby AM, Titley J, Agrawal RK, Alhasso A, Bhattacharya IS, Brunt AM, Ciurlionis L, Chan C, Donovan EM, Emson MA, Harnett AN, Haviland JS, Hopwood P, Jefford ML, Kaggwa R, Sawyer EJ, Syndikus I, Tsang YM, Wheatley DA, Wilcox M, Yarnold JR, Bliss JM; IMPORT Trialists. Partial-breast radiotherapy after breast conservation surgery for patients with early breast cancer (UK IMPORT LOW trial): 5-year results from a multicentre, randomised, controlled, phase 3, non-inferiority trial. Lancet. 2017 Sep 9;390(10099):1048-1060. doi: 10.1016/S0140-6736(17)31145-5. Epub 2017 Aug 2. PubMed PMID: 28779963; PubMed Central PMCID: PMC5594247.
21. Livi L, Meattini I, Marrazzo L, Simontacchi G, Pallotta S, Saieva C, Paiar F, Scotti V, De Luca Cardillo C, Bastiani P, Orzalesi L, Casella D, Sanchez L, Nori J, Fambrini M, Bianchi S. Accelerated partial breast irradiation using intensity-modulated radiotherapy versus whole breast irradiation: 5-year survival analysis of a phase 3 randomised controlled trial. Eur J Cancer. 2015 Mar;51(4):451-63. doi: 10.1016/j.ejca.2014.12.013. Epub 2015 Jan 17. PubMed PMID: 25605582.

22. Vicini FA, Cecchini RS, White JR, Julian TB, Arthur DW, Rabinovitch RA, Kuske RR, Parda DS, Ganz PA, Scheier MF, Winter KA, Paik S, Kuerer HM, Vallow LA, Pierce LJ, Mamounas EP, Costantino JP, Bear HD, Germaine I, Gustafson G, Grossheim L, Petersen IA, Hudes RS, Curran, Jr. WJ, Wolmark N. Primary results of NSABP B-39/RTOG 0413 (NRG Oncology): A randomized phase III study of conventional whole breast irradiation (WBI) versus partial breast irradiation (PBI) for women with stage 0, I, or II breast cancer. Oral presentation at SABCS Symposium 2018. Abstract GS4-04.
23. Veronesi U, Orecchia R, Maisonneuve P, Viale G, Rotmensz N, Sangalli C, Luini A, Veronesi P, Galimberti V, Zurrida S, Leonardi MC, Lazzari R, Cattani F, Gentilini O, Intra M, Caldarella P, Ballardini B. Intraoperative radiotherapy versus external radiotherapy for early breast cancer (ELIOT): a randomized controlled equivalence trial. Lancet Oncol. 2013 Dec;14(13):1269-77. doi: 10.1016/S1470-2045(13)70497-2. Epub 2013 Nov 11. PubMed PMID: 24225155.
24. Vaidya JS, Wenz F, Bulsara M, Tobias JS, Joseph DJ, Keshtgar M, Flyger HL, Massarut S, Alvarado M, Saunders C, Eiermann W, Metaxas M, Sperk E, Sütterlin M, Brown D, Esserman L, Roncadin M, Thompson A, Dewar JA, Holtveg HM, Pigorsch S, Falzon M, Harris E, Matthews A, Brew-Graves C, Potyka I, Corica T, Williams NR, Baum M; TARGIT trialists' group. Risk-adapted targeted intraoperative radiotherapy versus whole-breast radiotherapy for breast cancer: 5-year results for local control and overall survival from the TARGIT-A randomised trial. Lancet. 2014 Feb 15;383(9917):603-13. doi: 10.1016/S0140-6736(13)61950-9. Epub 2013 Nov 11. Erratum in: Lancet. 2014 Feb 15;383(9917):602. PubMed PMID: 24224997.
25. Vermeulen S, Cotrutz C, Morris A, Meier R, Buchanan C, Dawson P, Porter B. Accelerated Partial Breast Irradiation: Using the CyberKnife as the Radiation Delivery Platform in the Treatment of Early Breast Cancer. Front Oncol. 2011 Nov 21;1:43. doi: 10.3389/fonc.2011.00043. eCollection 2011. PubMed PMID: 22649764; PubMed Central PMCID: PMC3355980.
26. Vermeulen S, Haas JA. Cyberknife stereotactic body radiotherapy and cyberknife accelerated partial breast irradiation for the treatment of breast cancer. Transl Cancer Res 3(4):295-302, 2014. doi: 10.3978/j.issn.2218-676X.2014.07.06
27. Obayomi-Davies O, Kole TP, Oppong B, Rudra S, Makariou EV, Campbell LD, Anjum HM, Collins SP, Unger K, Willey S, Tousimis E, Collins BT. Stereotactic Accelerated Partial Breast Irradiation for Early-Stage Breast Cancer: Rationale, Feasibility, and Early Experience Using the CyberKnife Radiosurgery Delivery Platform. Front Oncol. 2016 May 23;6:129. doi: 10.3389/fonc.2016.00129. eCollection 2016. PubMed PMID: 27242967; PubMed Central PMCID: PMC4876543.
28. Rahimi A, Thomas K, Spangler A, Rao R, Leitch M, Wooldridge R, Rivers A, Seiler S, Albuquerque K, Stevenson S, Goudreau S, Garwood D, Haley B, Euhus D, Heinzerling J, Ding C, Gao A, Ahn C, Timmerman R. Preliminary Results of a Phase 1 Dose-Escalation Trial for Early-Stage Breast Cancer Using 5-Fraction Stereotactic Body Radiation Therapy for Partial-Breast Irradiation. Int J Radiat Oncol Biol Phys. 2017 May 1;98(1):196-205.e2. doi: 10.1016/j.ijrobp.2017.01.020. Epub 2017 Jan 12. PubMed PMID: 28586960.
29. Pezner RD, Tan MC, Clancy SL, Chen YJ, Joseph T, Vora NL. Radiation therapy for breast cancer patients who undergo oncoplastic surgery: localization of the tumor bed for the local boost. Am J Clin Oncol. 2013 Dec;36(6):535-9. doi: 10.1097/COC.0b013e318256efba. PubMed PMID: 22781391.
30. Jonczyk MM, Jean J, Graham R, Chatterjee A. Surgical trends in breast cancer: a rise in novel operative treatment options over a 12 year analysis. Breast Cancer Res Treat. 2019 Jan;173(2):267-274. doi: 10.1007/s10549-018-5018-1. Epub 2018 Oct 25. Review. PubMed PMID: 30361873.
31. Chen JY, Huang YJ, Zhang LL, Yang CQ, Wang K. Comparison of Oncoplastic Breast-Conserving Surgery and Breast-Conserving Surgery Alone: A Meta-Analysis. J Breast Cancer. 2018 Sep;21(3):321-329. doi: 10.4048/jbc.2018.21.e36. Epub 2018 Aug 28. PubMed PMID: 30275861; PubMed Central PMCID: PMC6158154.
32. De La Cruz L, Blankenship SA, Chatterjee A, Geha R, Nocera N, Czerniecki BJ, Tchou J, Fisher CS. Outcomes After Oncoplastic Breast-Conserving Surgery in Breast Cancer Patients: A Systematic Literature Review. Ann Surg Oncol. 2016 Oct;23(10):3247-58. doi: 10.1245/s10434-016-5313-1. Epub 2016 Jun 29. Review. PubMed PMID: 27357177.

33. Nichols E, Kesmodel SB, Bellavance E, Drogula C, Tkaczuk K, Cohen RJ, Citron W, Morgan M, Staats P, Feigenberg S, Regine WF. Preoperative Accelerated Partial Breast Irradiation for Early-Stage Breast Cancer: Preliminary Results of a Prospective, Phase 2 Trial. Int J Radiat Oncol Biol Phys. 2017 Mar 15;97(4):747-753. doi: 10.1016/j.ijrobp.2016.11.030. Epub 2016 Nov 27. PubMed PMID: 28244410.
34. van der Leij F, Bosma SC, van de Vijver MJ, Wesseling J, Vreeswijk S, Rivera S, Bourgier C, Garbay JR, Foukakis T, Lekberg T, van den Bongard DH, van Vliet-Vroegindeweij C, Bartelink H, Rutgers EJ, Elkhuizen PH. First results of the preoperative accelerated partial breast irradiation (PAPBI) trial. Radiother Oncol. 2015 Mar;114(3):322-7. doi: 10.1016/j.radonc.2015.02.002. Epub 2015 Feb 17. PubMed PMID: 25701298.
35. Nichols EM, Dhople AA, Mohiuddin MM, Flannery TW, Yu CX, Regine WF. Comparative analysis of the post-lumpectomy target volume versus the use of pre-lumpectomy tumor volume for early-stage breast cancer: implications for the future. Int J Radiat Oncol Biol Phys. 2010 May 1;77(1):197-202. doi: 10.1016/j.ijrobp.2009.04.063. PubMed PMID: 20394853.
36. Bondiau PY, Bahadoran P, Lallement M, Birtwisle-Peyrottes I, Chapellier C, Chamorey E, Courdi A, Quielle-Roussel C, Thariat J, Ferrero JM. Robotic stereotactic radioablation concomitant with neo-adjuvant chemotherapy for breast tumors. Int J Radiat Oncol Biol Phys. 2009 Nov 15;75(4):1041-7. doi: 10.1016/j.ijrobp.2008.12.037. Epub 2009 Apr 20. PubMed PMID: 19386428.
37. Bondiau PY, Courdi A, Bahadoran P, Chamorey E, Queille-Roussel C, Lallement M, Birtwisle-Peyrottes I, Chapellier C, Pacquelet-Cheli S, Ferrero JM. Phase 1 clinical trial of stereotactic body radiation therapy concomitant with neoadjuvant chemotherapy for breast cancer. Int J Radiat Oncol Biol Phys. 2013 Apr 1;85(5):1193-9. doi: 10.1016/j.ijrobp.2012.10.034. Epub 2013 Jan 16. PubMed PMID: 23332384.
38. Palta M, Yoo S, Adamson J. Preoperative single fraction partial breast radiotherapy for early-stage breast cancer. Int J Radiat Oncol Biol 82(1): 37-42, 2012
39. Blitzblau RC, Arya R, Yoo S, Baker JA, Chang Z, Palta M, Duffy E, Horton JK. A phase 1 trial of preoperative partial breast radiation therapy: Patient selection, target delineation, and dose delivery. Pract Radiat Oncol. 2015 Sep-Oct;5(5):e513-e520. doi: 10.1016/j.prro.2015.02.002. Epub 2015 Mar 31. PubMed PMID: 25834942; PubMed Central PMCID: PMC4568137.
40. Horton JK, Blitzblau RC, Yoo S, Geradts J, Chang Z, Baker JA, Georgiade GS, Chen W, Siamakpour-Reihani S, Wang C, Broadwater G, Groth J, Palta M, Dewhirst M, Barry WT, Duffy EA, Chi JT, Hwang ES. Preoperative Single-Fraction Partial Breast Radiation Therapy: A Novel Phase 1, Dose-Escalation Protocol With Radiation Response Biomarkers. Int J Radiat Oncol Biol Phys. 2015 Jul 15;92(4):846-55. doi: 10.1016/j.ijrobp.2015.03.007. Epub 2015 Mar 14. PubMed PMID: 26104938; PubMed Central PMCID: PMC4481883.
41. Vasmel J, Charaghvandi R, Houweling A, et al. Response after MR-guided single dose ablative preoperative partial breast irradiation. ESTRO 2019
42. Park C, Papiez L, Zhang S, Story M, Timmerman RD. Universal survival curve and single fraction equivalent dose: useful tools in understanding potency of ablative radiotherapy. Int J Radiat Oncol Biol Phys. 2008 Mar 1;70(3):847-52. doi: 10.1016/j.ijrobp.2007.10.059. PubMed PMID: 18262098.
43. Yu CX, Shao X, Zhang J, Regine W, Zheng M, Yu YS, Deng J, Duan Z. GammaPod-a new device dedicated for stereotactic radiotherapy of breast cancer. Med Phys. 2013 May;40(5):051703. doi: 10.1118/1.4798961. PubMed PMID: 23635251; PubMed Central PMCID: PMC3637326.
44. Yu Cx, Regine W, Zheng M, Zhang J, Feigenberg SJ. Stereotactic radiosurgery for early-stage breast cancer: a new paradigm. JCO 29(27_suppl): 120-120. 2011.

45. Huang SY, Franc BL, Harnish RJ, et al. Exploration of PET and MRI radiomic features for decoding breast cancer phenotypes and prognosis. *NPJ Breast Cancer.* 2018;4:24.
46. Saha A, Yu X, Sahoo D, Mazurowski MA. Effects of MRI scanner parameters on breast cancer radiomics. *Expert Syst Appl.* 2017;87:384-391.
47. Drukker K, Li H, Antropova N, Edwards A, Papaioannou J, Giger ML. Most-enhancing tumor volume by MRI radiomics predicts recurrence-free survival "early on" in neoadjuvant treatment of breast cancer. *Cancer imaging : the official publication of the International Cancer Imaging Society.* 2018;18(1):12.

STU2019-1183, Rahimi, FormA-Protocol, Mod_15, 11-01-23 (7)

48. Saha A, Harowicz MR, Mazurowski MA. Breast cancer MRI radiomics: An overview of algorithmic features and impact of inter-reader variability in annotating tumors. *Med Phys.* 2018;45(7):3076-3085.
49. Li H, Zhu Y, Burnside ES, et al. MR Imaging Radiomics Signatures for Predicting the Risk of Breast Cancer Recurrence as Given by Research Versions of MammaPrint, Oncotype DX, and PAM50 Gene Assays. *Radiology.* 2016;281(2):382-391.
50. Fan M, Wu G, Cheng H, Zhang J, Shao G, Li L. Radiomic analysis of DCE-MRI for prediction of response to neoadjuvant chemotherapy in breast cancer patients. *Eur J Radiol.* 2017;94:140-147.
51. Zhou Z, Folkert M, Iyengar P, et al. Multi-objective radiomics model for predicting distant failure in lung SBRT. *Phys Med Biol.* 2017;62(11):4460-4478.
52. Hao H, Zhou Z, Li S, et al. Shell feature: a new radiomics descriptor for predicting distant failure after radiotherapy in non-small cell lung cancer and cervix cancer [published online ahead of print 2018/04/05]. *Phys Med Biol.* 2018;63(9):095007.

13.0 APPENDICES

APPENDIX I

ZUBROD PERFORMANCE SCALE

0	*Fully active, able to carry on all predisease activities without restriction (Karnofsky 90-100).*
1	*Restricted in physically strenuous activity but ambulatory and able to carry work of a light or sedentary nature. For example, light housework, office work (Karnofsky 70-80).*
2	*Ambulatory and capable of all self-care but unable to carry out any work activities. Up and about more than 50% of waking hours (Karnofsky 50-60).*
3	*Capable of only limited self-care, confined to bed or chair 50% or more of waking hours (Karnofsky 30-40).*
4	*Completely disabled. Cannot carry on self-care. Totally confined to bed or (Karnofsky 10-20).*
5	*Death (Karnofsky 0).*

KARNOFSKY PERFORMANCE SCALE

100	*Normal; no complaints; no evidence of disease*
90	*Able to carry on normal activity; minor signs or symptoms of disease*
80	*Normal activity with effort; some sign or symptoms of disease*
70	*Cares for self; unable to carry on normal activity or do active work*
60	*Requires occasional assistance, but is able to care for most personal needs*
50	*Requires considerable assistance and frequent medical care*
40	*Disabled; requires special care and assistance*
30	*Severely disabled; hospitalization is indicated, although death not imminent*
20	*Very sick; hospitalization necessary; active support treatment is necessary*
10	*Moribund; fatal processes progressing rapidly*
0	*Dead*

APPENDIX II

AJCC STAGING SYSTEM
BREAST, 8th Edition

DEFINITION OF TNM

Primary Tumor, Clinical (T) and Pathological
Definitions for classifying the primary tumor (T) are the same for clinical and for pathologic classification. If the measurement is made by physical examination, the examiner will use the major headings (T1, T2, or T3). If other measurements, such as mammographic or pathologic measurements, are used, the subsets of T1 can be used. Tumors should be measured to the nearest 0.1 cm increment.

TX Primary tumor cannot be assessed
T0 No evidence of primary tumor
Tis Carcinoma in situ
Tis (DCIS) Ductal carcinoma in situ
Tis (LCIS) Lobular carcinoma in situ
Tis (Paget's) Paget's disease of the nipple with no tumor

Note: Paget's disease associated with a tumor is classified according to the size of the tumor.

T1 Tumor 2 cm or less in greatest dimension
T1mic Microinvasion 0.1 cm or less in greatest dimension
T1a Tumor more than 0.1 but not more than 0.5 cm in greatest dimension
T1b Tumor more than 0.5 cm but not more than 1 cm in greatest dimension
T1c Tumor more than 1 cm but not more than 2 cm in greatest dimension
T2 Tumor more than 2 cm but not more than 5 cm in greatest dimension
T3 Tumor more than 5 cm in greatest dimension
T4 Tumor of any size with direct extension to (a) chest wall or (b) skin, only as described below.
T4a Extension to chest wall, not including pectoralis muscle
T4b Edema (including peau d' orange) or ulceration of the skin of the breast, or satellite skin nodules confined to the same breast
T4c Both T4a and T4b
T4d Inflammatory carcinoma

Regional Lymph Nodes (N)

Clinical
NX Regional lymph nodes cannot be assessed (e.g., previously removed)
N0 No regional lymph node metastasis
N1 Metastasis to movable ipsilateral axillary lymph node(s) level I, II
N2 Metastasis in ipsilateral axillary lymph nodes fixed or matted, or in clinically apparent* ipsilateral internal mammary nodes in the *absence* of clinically evident axillary lymph node metastasis
N2a Metastasis in ipsilateral axillary lymph nodes fixed to one another (matted) or to other structures
N2b Metastasis only in clinically apparent* ipsilateral internal mammary nodes and in the *absence* of clinically evident axillary lymph node metastasis
N3 Metastasis in ipsilateral infraclavicular lymph node(s) with or without axillary lymph node involvement, or in clinically apparent* ipsilateral internal mammary lymph node(s) and in the *presence* of clinically evident axillary lymph node metastasis; or metastasis in ipsilateral supraclavicular lymph node(s) with or without axillary or internal mammary lymph node involvement
N3a Metastasis in ipsilateral infraclavicular lymph node(s)

APPENDIX II (continued)

AJCC STAGING SYSTEM
BREAST, 8th Edition

N3b Metastasis in ipsilateral internal mammary lymph node(s) and axillary lymph node(s)
N3c Metastasis in ipsilateral supraclavicular lymph node(s)

* Clinically apparent is defined as detected by imaging studies (excluding lymphoscintigraphy) or by clinical examination or grossly visible pathologically.

Pathologic (pN)[a]

pNX Regional lymph nodes cannot be assessed (e.g., previously removed, or not removed for pathologic study)
pN0 No regional lymph node metastasis histologically, no additional examination for isolated tumor cells (ITC)

Note: Isolated tumor cells (ITC) are defined as single tumor cells or small cell clusters not greater than 0.2 mm, usually detected only by immunohistochemical (IHC) or molecular methods but which may be verified on H&E stains. ITCs do not usually show evidenced of malignant activity e.g., proliferation or stromal reaction.

pNO(i-) No regional lymph node metastasis histologically, negative IHC
pNO(i+) No regional lymph node metastasis histologically, positive IHC, no IHC cluster greater than 0.2mm pNO(mol-) No regional lymph node metastasis histologically, negative molecular findings (RT-PCR)b
pNO(mol+) No regional lymph node metastasis histologically, positive molecular findings (RT-PCR)b

[a]Classification is based on axillary lymph node dissection with or without sentinel lymph node dissection. Classification based solely on sentinel lymph node dissection without subsequent axillary lymph node dissection is designated (sn) for “sentinel node,” e.g., pNO(i+) (sn).
[b]RT-PCR:reverse transcriptase/polymerase chain reaction.

pN1 Metastasis in 1 to 3 axillary lymph nodes, and/or in internal mammary nodes with microscopic disease detected by sentinel lymph node dissection but not clinically apparent**
pN1mi Micrometastasis (greater than 0.2 mm, none greater than 2.0 mm)
pN1a Metastasis in 1 to 3 axillary lymph nodes
pN1b Metastasis in internal mammary nodes with microscopic disease detected by sentinel lymph node dissection but not clinically apparent**
pN1c Metastasis in 1 to 3 axillary lymph nodes and in internal mammary lymph nodes with microscopic disease detected by sentinel lymph node dissection but not clinically apparent.** (If associated with greater than 3 positive axillary lymph nodes, the internal mammary nodes are classified as pN3b to reflect increased tumor burden).
pN2 Metastasis in 4 to 9 axillary lymph nodes, or in clinically apparent* internal mammary lymph nodes in the *absence* of axillary lymph node metastasis
pN2a Metastasis in 4 to 9 axillary lymph nodes (at least one tumor deposit greater than 2.0 mm)
pN2b Metastasis in clinically apparent* internal mammary lymph nodes in the *absence* of axillary lymph node metastasis
pN3 Metastasis in 10 or more axillary lymph nodes, or in infraclavicular lymph nodes, or in clinically apparent* ipsilateral internal mammary lymph nodes in the *presence* of 1 or more positive axillary lymph nodes; or in more than 3 axillary lymph nodes with clinically negative microscopic metastasis in internal mammary lymph nodes; or in ipsilateral supraclavicular lymph nodes

APPENDIX II (continued)

AJCC STAGING SYSTEM
BREAST, 8th Edition

pN3a Metastasis in 10 or more axillary lymph nodes (at least one tumor deposit greater than 2.0 mm), or metastasis to the infraclavicular lymph nodes

pN3b Metastasis in clinically apparent* ipsilateral internal mammary lymph nodes in the *presence* of 1 or more positive axillary lymph nodes; or in more than 3 axillary lymph nodes and in internal mammary lymph nodes with microscopic disease detected by sentinel lymph node dissection but not clinically apparent**

pN3c Metastasis in ipsilateral supraclavicular lymph nodes

**Clinically apparent* is defined as detected by imaging studies (excluding lymphoscintigraphy) or by clinical examination.
***Not clinically apparent* is defined as not detected by imaging studies (excluding lymphoscintigraphy) or by clinical examination.

Distant Metastasis (M)*

MX	Presence of distant metastasis cannot be assessed (not evaluated by any modality)
M0	No distant metastasis
M1	Distant metastasis

Appendix III

Patient Cosmesis Evaluation

Patient Name: _____________ Study ID: ______________ Date: ________________

Patient describes cosmesis as:

1	**EXCELLENT:** when compared to the untreated breast or the original appearance of the breast, there is minimal or no difference in the size or shape of the treated breast. The way the breast feels (its texture) is the same or slightly different. There may be thickening, scar tissue or fluid accumulation within the breast, but not enough to change the appearance.
2	**GOOD:** there is slight difference in the size or shape of the treated breast as compared to the opposite breast or the original appearance of the treated breast. There may be some mild reddening or darkening of the breast. The thickening or scar tissue within the breast causes only a mild change in the shape or size.
3	**FAIR:** obvious differences in the size and shape of the treated breast. This change a quarter or less of the breast. There can be moderate thickening or scar tissue of the skin and the breast, and there may be obvious color changes.
4	**POOR:** marked change in the appearance of the treated breast involving more than a quarter of the breast tissue. The skin changes may obvious and detract from the appearance of the breast. Severe scarring and thickening of the breast, which clearly alters the appearance of the breast, may be found.

__________________________________ ________________________
Study coordinator Date

Appendix IV

Physician Cosmesis Evaluation

Patient Name: ____________ Study ID: ______________ Date: ________________

Please assess breast cosmesis at this time:

1	**EXCELLENT:** when compared to the untreated breast or the original appearance of the breast, there is minimal or no difference in the size or shape of the treated breast. The way the breast feels (its texture) is the same or slightly different. There may be thickening, scar tissue or fluid accumulation within the breast, but not enough to change the appearance.
2	**GOOD:** there is slight difference in the size or shape of the treated breast as compared to the opposite breast or the original appearance of the treated breast. There may be some mild reddening or darkening of the breast. The thickening or scar tissue within the breast causes only a mild change in the shape or size.
3	**FAIR:** obvious differences in the size and shape of the treated breast. This change a quarter or less of the breast. There can be moderate thickening or scar tissue of the skin and the breast, and there may be obvious color changes.
4	**POOR:** marked change in the appearance of the treated breast involving more than a quarter of the breast tissue. The skin changes may obvious and detract from the appearance of the breast. Severe scarring and thickening of the breast, which clearly alters the appearance of the breast, may be found.

	None	Yes, present but does not affect cosmesis	Yes, present and affects cosmesis
Skin telangiectasia	0	1	2
Skin atrophy	0	1	2
Scarring	0	1	2
Pigment change	0	1	2
Erythema	0	1	2
Fat necrosis	0	1	2
Fibrosis	0	1	2
Retraction or contour defect	0	1	2
Volume loss	0	1	2
Other significant treatment effects	0	1	2

Specify:

___________________________________ ____________________________

Signature Date
