# Supplementary material for: Ablative Preoperative Single-Fraction Radiation Dose Escalation Among Patients With Breast Cancer: A Phase 1 Nonrandomized Clinical Trial
Source: JAMA Netw Open. 2025 Nov 14;8(11):e2543689. doi: 10.1001/jamanetworkopen.2025.43689 (PMC12619099; doi:10.1001/jamanetworkopen.2025.43689)
Supplement: Supplement 2. — eAppendix 1. Dosimetric Constraints for Single-Fraction SABR eAppendix 2. Imaging Assessment of Response eAppendix 3. Ki-67 of Biopsy Specimens at Diagnosis and After Preoperative Radiation and Delayed Surgery eAppendix 4. Univariate and Multiple Logistic Regression Results Evaluating the Association of Time to Surgery and Tumor Size With Dichotomized Miller Payne Scores eAppendix 5. Receiver Operating Characteristic (ROC) Curves for Miller Payne Scores eAppendix 6. ROC Analysis and Accuracy for pCR eAppendix 7. Patient Cosmesis eAppendix 8. Physician Cosmesis eAppendix 9. Patient and Physician Cosmesis Results [file jamanetwopen-e2543689-s002.pdf]

Supplemental Online Content

Rahimi A, Leitch M, Dogan B, et al. Ablative preoperative single-fraction radiation dose escalation among patients with breast cancer: a phase 1 nonrandomized clinical trial. *JAMA Netw Open*. 2025;8(11):e2543689. doi:10.1001/jamanetworkopen.2025.43689

eAppendix 1. Dosimetric Constraints for Single-Fraction SABR

eAppendix 2. Imaging Assessment of Response

eAppendix 3. Ki-67 of Biopsy Specimens at Diagnosis and After Preoperative Radiation and Delayed Surgery

eAppendix 4. Univariate and Multiple Logistic Regression Results Evaluating the Association of Time to Surgery and Tumor Size With Dichotomized Miller Payne Scores

eAppendix 5. Receiver Operating Characteristic (ROC) Curves for Miller Payne Scores

eAppendix 6. ROC Analysis and Accuracy for pCR

eAppendix 7. Patient Cosmesis

eAppendix 8. Physician Cosmesis

eAppendix 9. Patient and Physician Cosmesis Results

This supplemental material has been provided by the authors to give readers additional information about their work.

© 2025 Rahimi A et al. *JAMA Network Open*.

eAppendix 1. Dosimetric Constraints for Single-Fraction SABR

Serial Tissue	Volume	Volume Max (Gy)	Max Point Dose (Gy)**	Endpoint (≥Grade 3)
Spinal Cord and medulla	<0.35 cc	10 Gy	14 Gy	myelitis
Brachial Plexus	<3 cc	13.6 Gy	16.4 Gy	neuropathy
Heart	<15 cc	16 Gy	22 Gy	pericarditis
Trachea and Large Bronchus* (Ck only)	<4 cc	17.4 Gy	20.2 Gy	stenosis/fistula
Bronchus-smaller airways (CK only)	<0.5 cc	12.4 Gy	13.3 Gy	stenosis with atelectasis
Rib	<5 cc	28 Gy	33 Gy	Pain or fracture
Skin	<10 cc	25.5 Gy	27.5 Gy	ulceration
**Parallel Tissue**	Critical Volume (cc)	**Critical Volume Dose Max (Gy)**		**Endpoint (≥Grade 3)**
Thyroid gland (CK only)	5 cc	15 Gy		Hypothyroid
Ipsilateral Breast	<40% of whole breast reference volume		>/= 50% prescribed dose	Cosmesis
Ipsilateral Breast	<20% of whole breast reference volume		>/= 100% prescribed dose	Cosmesis
Mean Heart dose				Please record value
Lung (Right & Left) CK only	1500 cc	7 Gy		Basic Lung Function
Lung (Right & Left) CK only<br>Chest Wall	1000 cc<br><30cc	7.6 Gy<br>30 Gy	V-8Gy <37%	Pneumonitis<br>Chest wall pain

***Avoid circumferential irradiation**
**** "point" defined as 0.035cc or less**

Dosimetry Compliance
Exceeding dosimetric limits by more than 2.5% constitutes a minor protocol violation. Exceeding dosimetric limits by more than 5% constitutes a major protocol violation.

Radiation Planning and Target Goals for Single Fraction breast SABR:

- 95% of the PTV should receive a minimum of 27 Gy and 99% of the target volume (GTV) receives a minimum of 93% of the prescription dose. Minor

© 2025 Rahimi A et al. *JAMA Network Open*.

deviation if 90% of PTV receives 27 Gy. All efforts should be made to meet the skin constraint even if PTV coverage will be compromised. In this situation, would prioritize skin, brachial plexus, and spinal cord constraint over PTV coverage and this is acceptable and not a deviation, and will not be deemed a protocol violation if PTV coverage not met in order to meet these OAR constraints. Efforts should be made to keep the PTV volume coverage to a minimum dose of 27 Gy to 95% of volume. In the situation when the GTV is close to skin, prioritize the skin constraint over GTV coverage.

- Max hot spot < 130% of the prescription dose is recommended
- Prescription will be either 30 Gy, 34 Gy, or 38 Gy to the GTV/CTV per appropriate dose cohort.

<u>Critical Organ Dose-Volume Limits</u>

Priority 1: Spinal cord, brachial plexus, skin, trachea, large and small bronchus
Priority 2: Cover PTV
Priority 3: Heart, rib, thyroid, lung

© 2025 Rahimi A et al. *JAMA Network Open*.

eAppendix 2. Imaging Assessment of Response

Imaging assessment of response

All patients underwent mammography and breast ultrasound (US) at baseline and if clinically indicated, contrast enhanced dynamic (DCE) breast MRI. Post SPBI imaging follow up with mammography and US was performed at 4-6 months and pre-operative MRI 6-9 months. All breast MRIs were performed in a 1.5T unit (Magnetom Sola Siemens, Germany) using a dedicated 8 or 18 channel coil (Invivo Sentinelle, or Siemens 18, Siemens, Germany) with patients lying prone. A single pre contrast and four serial bilateral axial dynamic image sets were obtained before and immediately after rapid intravenous bolus infusion of 0.1 mmol/kg gadobutrol (Gadavist; Bayer Healthcare Pharmaceuticals) at a rate of 3 ml/sec with a power injector (Spectris Solaris MR injector; Medrad), with an average dynamic temporal resolution of 90 sec/phase. Delayed high spatial resolution post contrast sagittal T1 weighted sequence and a fluid bright series were obtained, along with diffusion weighted series. Absence of enhancement on pre-surgery MRI exam (early and delayed sequences) was considered complete imaging response.

© 2025 Rahimi A et al. *JAMA Network Open*.

eAppendix 3. Ki-67 of Biopsy Specimens at Diagnosis and After Preoperative Radiation and Delayed Surgery

Comparison of Ki-67 in biopsy specimens at diagnosis and after pre-operative radiation and delayed surgery

**Radiation dose**	**Ki67 at diagnosis**<br>**Mean (±± SD)**<br>**Median (IQR)**	**Ki67 on evaluable residual disease**<br>**Mean (±±SD)**<br>**Mean (IQR)**	***P* value**<br>†ϯ
**30 Gy**	11.9%<br>±6.6%<br>10% (9%, 15%)	1.4%<br>±0.5%<br>1% (1%, 2%)	0.008*
**34 Gy**	11.5%<br>±6.4%<br>12% (6.5%, 16.5%)	2.4%<br>±3.2%<br>1% (1%, 2.25%)	0.005*
**38 GY**	10.4%<br>±6.7%<br>8% (6%, 14.5%)	1.8%<br>±1%<br>1.5% (1%, 2.25%)	0.111
**30+34+38 Gy**	11.3%<br>±6.4%<br>10% (7.5%, 15%)	1.9%<br>±2%<br>1% (1%, 2%)	<0.001*

†ϯ
: *P* values were calculated using paired t-test or Wilcoxon signed-rank test based on paired data.

© 2025 Rahimi A et al. *JAMA Network Open*.

eAppendix 4. Univariate and Multiple Logistic Regression Results Evaluating the Association of Time to Surgery and Tumor Size With Dichotomized Miller Payne Scores

Univariate and multiple logistic regression results evaluating the effect of time to surgery and tumor size on dichotomized Miller Payne scores (≥4 cutoff)				
	**Univariate logistic regression**		**Multiple logistic regression**	
**Variable**	**OR (95% CI)**	***p* value**	**OR (95% CI)**	***p* value**
Time to surgery (day)	1.015 (1.004, 1.027)	0.011*	1.019 (1.005, 1.033)	0.009*
Tumor size (mm)	1.010 (0.883, 1.156)	0.881	0.910 (0.771, 1.073)	0.261

Univariate and multiple logistic regression results evaluating the effect of time to surgery and tumor size on dichotomized Miller Payne scores (≥5 cutoff)				
	**Univariate logistic regression**		**Multiple logistic regression**	
**Variable**	**OR (95% CI)**	***p* value**	**OR (95% CI)**	***p* value**
Time to surgery (day)	1.015 (1.005, 1.025)	0.002*	1.017 (1.006, 1.028)	0.002*
Tumor size (mm)	1.020 (0.925, 1.123)	0.696	0.943 (0.839, 1.060)	0.328

© 2025 Rahimi A et al. *JAMA Network Open*.

eAppendix 5. Receiver Operating Characteristic (ROC) Curves for Miller Payne Scores

The left panel illustrates the ROC curve for differentiating Miller-Payne score ≥4 versus <4, while the right panel depicts the ROC curve for Miller-Payne score ≥5 versus <5. The performance of time to surgery (red), tumor size (blue), and their combination (green) are shown with respective area under the curve (AUC) values and optimal cutoff points.

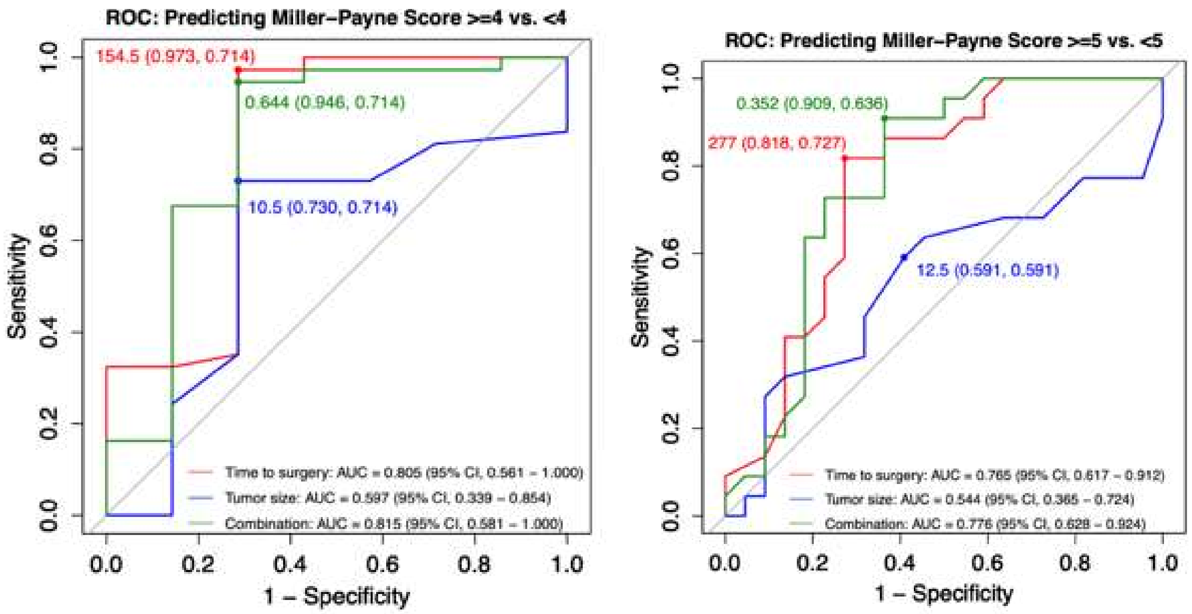

© 2025 Rahimi A et al. *JAMA Network Open*.

eAppendix 6. ROC Analysis and Accuracy for pCR

ROC analysis and diagnostic accuracy results for predicting Miller-Payne scores ≥4 vs. <4						
**Biomarkers**	**Threshold**	**AUC (95% CI)**	**Sensitivity**	**Specificity**	**PPV**	**NPV**
**Single Factor**						
Time to surgery (day)	154.5	0.805 (0.561 – 1.000)	0.973	0.714	0.947	0.833
Tumor size (mm)	10.5	0.597 (0.339 – 0.854)	0.730	0.714	0.931	0.333
**Combined Factors**						
Time to surgery + tumor size	0.644 (in probability)	0.815 (0.581 – 1.000)	0.946	0.714	0.946	0.714

ROC analysis and diagnostic accuracy results for predicting Miller-Payne scores ≥5 vs. <5						
**Biomarkers**	**Threshold**	**AUC (95% CI)**	**Sensitivity**	**Specificity**	**PPV**	**NPV**
**Single Factors**						
Time to surgery (day)	277	0.765 (0.617 – 0.912)	0.818	0.727	0.750	0.800
Tumor size (mm)	12.5	0.544 (0.365 – 0.724)	0.591	0.591	0.591	0.591
	16.5	0.544 (0.365 – 0.724)	0.318	0.864	0.700	0.558
**Combined Factors**						
Time to surgery + tumor size	0.352 (in probability)	0.776 (0.628 – 0.924)	0.909	0.636	0.714	0.875

© 2025 Rahimi A et al. *JAMA Network Open*.

eAppendix 7. Patient Cosmesis

Patient Cosmesis Evaluation

Patient Name: _____________ Study ID: ______________ Date: ________________

Patient describes cosmesis as:

1	**EXCELLENT:** when compared to the untreated breast or the original appearance of the breast, there is minimal or no difference in the size or shape of the treated breast. The way the breast feels (its texture) is the same or slightly different. There may be thickening, scar tissue or fluid accumulation within the breast, but not enough to change the appearance.
2	**GOOD:** there is slight difference in the size or shape of the treated breast as compared to the opposite breast or the original appearance of the treated breast. There may be some mild reddening or darkening of the breast. The thickening or scar tissue within the breast causes only a mild change in the shape or size.
3	**FAIR:** obvious differences in the size and shape of the treated breast. This change a quarter or less of the breast. There can be moderate thickening or scar tissue of the skin and the breast, and there may be obvious color changes.
4	**POOR:** marked change in the appearance of the treated breast involving more than a quarter of the breast tissue. The skin changes may obvious and detract from the appearance of the breast. Severe scarring and thickening of the breast, which clearly alters the appearance of the breast, may be found.

___________________________________ ________________________

Study coordinator Date

© 2025 Rahimi A et al. *JAMA Network Open*.

eAppendix 8. Physician Cosmesis

Physician Cosmesis Evaluation

Patient Name: ___________ Study ID: _____________ Date: _______________

Please assess breast cosmesis at this time:

1	**EXCELLENT:** when compared to the untreated breast or the original appearance of the breast, there is minimal or no difference in the size or shape of the treated breast. The way the breast feels (its texture) is the same or slightly different. There may be thickening, scar tissue or fluid accumulation within the breast, but not enough to change the appearance.
2	**GOOD:** there is slight difference in the size or shape of the treated breast as compared to the opposite breast or the original appearance of the treated breast. There may be some mild reddening or darkening of the breast. The thickening or scar tissue within the breast causes only a mild change in the shape or size.
3	**FAIR:** obvious differences in the size and shape of the treated breast. This change a quarter or less of the breast. There can be moderate thickening or scar tissue of the skin and the breast, and there may be obvious color changes.
4	**POOR:** marked change in the appearance of the treated breast involving more than a quarter of the breast tissue. The skin changes may obvious and detract from the appearance of the breast. Severe scarring and thickening of the breast, which clearly alters the appearance of the breast, may be found.

	None	Yes, present but does not affect cosmesis	Yes, present and affects cosmesis
Skin telangiectasia……………………	0 ……………………	.1 ……………………	2
Skin atrophy…….……………………	0 ……………………	.1 ……………………	2
Scarring……………..…………………	0 ……………………	.1 ……………………	2
Pigment change…..……………………	0 ……………………	.1 ……………………	2
Erythema………...……………………	0 ……………………	.1 ……………………	2
Fat necrosis……...……………………	0 ……………………	.1 ……………………	2
Fibrosis……………..…………………	0 ……………………	.1 ……………………	2
Retraction or contour defect ………..	0 ……………………	.1 ……………………	2
Volume loss……..……………………	0 ……………………	.1 ……………………	2
Other significant treatment effects …	0 ……………………	.1 ……………………	2

Specify:

________________________________ ______________________________

Signature Date

© 2025 Rahimi A et al. *JAMA Network Open*.

eAppendix 9. Patient and Physician Cosmesis Results

Patient reported cosmesis at enrollment and 36 month follow-up

**Patient reported score**		**Patient reported score**			
**Cohort**	**Event Name**	**1 - Excellent**	**2 - Good**	**3 - Fair**	**Grand Total**
**30 Gy**	36 Month Follow Up	3	2	2	7
	Enrollment	5	7	2	14
**30 Gy Total**		**8**	**9**	**4**	**21**
	P value- McNemars test				**p>0.999**
**34 Gy**	36 Month Follow Up	3	2	3	8
	Enrollment	11	4		15
**34 Gy Total**		**14**	**6**	**3**	**23**
	P value- McNemars test				**p=0.248**

Physician reported cosmesis at enrollment and 36 month follow-up

**MD assessment score**		**MD assessment score**			
**Cohort**	**Event Name**	**1 - Excellent**	**2 - Good**	**3 - Fair**	**Grand Total**
**30 Gy**	36 Month Follow Up	2	4	1	7
	Enrollment	13		1	14
**30 Gy Total**		**15**	**4**	**2**	**21**
	P value-McNemars test				**p>0.999**
**34 Gy**	36 Month Follow Up	5	3		8
	Enrollment	13	2		15
**34 Gy Total**		**18**	**5**		**23**
	P value exact binomial test				**p>0.999**

© 2025 Rahimi A et al. *JAMA Network Open*.
