# Supplementary material for: Ablative Preoperative Single-Fraction Radiation Dose Escalation Among Patients With Breast Cancer: A Phase 1 Nonrandomized Clinical Trial
Source: JAMA Netw Open. 2025 Nov 14;8(11):e2543689. doi: 10.1001/jamanetworkopen.2025.43689 (PMC12619099; doi:10.1001/jamanetworkopen.2025.43689)
Supplement: Supplement 3. — Data Sharing Statement [file jamanetwopen-e2543689-s003.pdf]

Data Sharing Statement

Rahimi. Ablative Preoperative Single-Fraction Radiation Dose Escalation Among Patients With Breast Cancer. *JAMA Netw Open*. Published November 14, 2025.
doi:10.1001/jamanetworkopen.2025.43689

Data
Additional Information: Trial Registration: clinicaltrials.gov(NCT04040569)
https://clinicaltrials.gov/study/NCT04040569
Data available: No

Additional Information
Explanation for why data not available: This will occur on a case by case situation
